# Hydrophobic Mismatch Drives the Interaction of E5 with the Transmembrane Segment of PDGF Receptor

**DOI:** 10.1016/j.bpj.2015.07.022

**Published:** 2015-08-18

**Authors:** Dirk Windisch, Colin Ziegler, Stephan L. Grage, Jochen Bürck, Marcel Zeitler, Peter L. Gor’kov, Anne S. Ulrich

**Affiliations:** 1 Institute of Biological Interfaces (IBG-2), Karlsruhe Institute of Technology, Karlsruhe, Germany; 2 Institute of Organic Chemistry, Karlsruhe Institute of Technology, Karlsruhe, Germany; 3 National High Magnetic Field Laboratory, Tallahassee, Florida

## Abstract

The oncogenic E5 protein from bovine papillomavirus is a short (44 amino acids long) integral membrane protein that forms homodimers. It activates platelet-derived growth factor receptor (PDGFR) *β* in a ligand-independent manner by transmembrane helix-helix interactions. The nature of this recognition event remains elusive, as numerous mutations are tolerated in the E5 transmembrane segment, with the exception of one hydrogen-bonding residue. Here, we examined the conformation, stability, and alignment of the E5 protein in fluid lipid membranes of substantially varying bilayer thickness, in both the absence and presence of the PDGFR transmembrane segment. Quantitative synchrotron radiation circular dichroism analysis revealed a very long transmembrane helix for E5 of ∼26 amino acids. Oriented circular dichroism and solid-state ^15^N-NMR showed that the alignment and stability of this unusually long segment depend critically on the membrane thickness. When reconstituted alone in exceptionally thick DNPC lipid bilayers, the E5 helix was found to be inserted almost upright. In moderately thick bilayers (DErPC and DEiPC), it started to tilt and became slightly deformed, and finally it became aggregated in conventional DOPC, POPC, and DMPC membranes due to hydrophobic mismatch. On the other hand, when E5 was co-reconstituted with the transmembrane segment of PDGFR, it was able to tolerate even the most pronounced mismatch and was stabilized by binding to the receptor, which has the same hydrophobic length. As E5 is known to activate PDGFR within the thin membranes of the Golgi compartment, we suggest that the intrinsic hydrophobic mismatch of these two interaction partners drives them together. They seem to recognize each other by forming a closely packed bundle of mutually aligned transmembrane helices, which is further stabilized by a specific pair of hydrogen-bonding residues.

## Introduction

Hydrophobic mismatch between lipid membranes and integral proteins can be an important regulator of protein function (1–3). A single-span (bitopic) transmembrane protein with a given hydrophobic length is expected to respond to the local membrane thickness. It has to avoid unfavorable exposure of hydrophobic regions to the hydrophilic environment by minimizing the energy of the hydrophobic mismatch. When a transmembrane domain (TMD) is very long, this can result in tilting of the helix, changes in the backbone conformation, or oligomerization (1,4). In the case of oligomers or compact polytopic membrane proteins, the entire bundle is obviously less sensitive to bilayer thickness.

Here, we investigated the mechanism of signal transduction by platelet-derived growth factor receptor (PDGFR) *β*, a single-span transmembrane protein. PDGFR belongs to the family of receptor tyrosine kinases (RTKs) that are involved in development, central nervous system formation, and angiogenesis (5). It is activated when a growth factor binds simultaneously to two receptor monomers. Dimerization leads to a rearrangement of the TMDs that brings the cytosolic kinase domains into close contact, enabling them to undergo transautophosphorylation and trigger signal transduction (6,7). The dimer contact encompasses the extramembranous domains, but the TMDs are also actively involved in this allosteric activation process (8). Many RTKs, including PDGFR, are known to be assembled as preformed dimers (8–11) in which the transmembrane segments can rotate from an inactive to an activated state upon ligand binding. Our recent structural investigations of the PDGFR-TMD suggested that the helix tilt angle and the stability of the preformed dimer are controlled by hydrophobic matching to the lipid bilayer thickness (12).

PDGFR can also be activated in a ligand-independent manner by the oncogenic E5 protein from bovine papillomavirus (13–20). With only 44 amino acids, this is one of the shortest known integral membrane proteins. E5 activates the receptor through highly specific interactions between the transmembrane segments, the nature of which is not yet fully understood (21–23). Complex formation is known to slow down receptor internalization, and the sustained signaling can lead to cancer. To be able to interact with PDGFR, E5 itself needs to be present as a dimer, which is maintained by two disulfide bridges within a short extramembranous stretch at the C-terminus. Interestingly, E5 mutants lacking these cysteines or even the entire C-terminal region are still able to form dimers (24–28), suggesting that dimerization is also driven by specific interhelical contacts between the TMDs. In infected cells, the E5 protein is found in the plasma membrane (29) but is located predominantly in the Golgi compartment, where it is able to activate PDGFR (30).

Structural investigations using infrared and circular dichroism (CD) spectroscopy have shown that E5 adopts a predominantly *α*-helical conformation when reconstituted in DMPC lipid bilayers and detergent micelles (22,24,25,31), but to date no high-resolution structure is available for E5 alone or in a complex with PDGFR. In general, it is very challenging to handle E5 because this highly hydrophobic protein has an intrinsic high tendency to aggregate, and the native sequence forms higher-mass oligomers due to nonspecific disulfide cross-linking. We recently demonstrated that we could resolve the latter problem by removing the cysteines by C-terminal truncation (28). The resulting truncated ΔE5 variant (containing the first 34 amino acids of the native sequence) is much more manageable and at the same time retains the same secondary structure, the same orientation in the membrane, and the same ability to self-associate as the wild-type protein. Thus, ΔE5 may be regarded as a representative model for the E5 TMD, and we used it here to conduct a detailed structure analysis in lipid bilayers.

Solid-state NMR and CD spectroscopy are ideally suited for investigating the structures, protein-lipid interactions, and protein-protein recognition of E5 and PDGFR. These methods can be routinely used to characterize the alignment of transmembrane helices in macroscopically oriented membrane samples. One-dimensional (1D) ^15^N-NMR spectra provide a good estimate of the helix tilt angle and will readily reveal nonspecific protein aggregation (12). Two-dimensional (2D) separated-local-field ^15^N-NMR experiments (32–37) display distinctive signal patterns, called polarity index slant angle (PISA) wheels, that can provide a direct measure of both the helix tilt angle and the azimuthal rotation angle (33,35,38). Such PISA wheels can be fitted to extract the structures of membrane proteins with high accuracy, as was done for the Vpu protein from HIV (39), the M2 protein channel of the influenza virus (40), and the M13 coat protein of bacteriophages (35).

Here, we examined the membrane alignment and stability of the E5 protein in lipid bilayers of varying thickness using a combination of ^15^N-NMR PISA wheel analysis, synchrotron radiation CD (SRCD), and oriented CD (OCD). We thoroughly characterized the behavior of E5 as a function of hydrophobic mismatch, both on its own and in the presence of PDGFR, which has the same hydrophobic length.

## Materials and Methods

### Cloning and expression of ΔE5 and PDGFR-TMD

The truncated mutant ΔE5 and the PDGFR-TMD were cloned and expressed as Trp-ΔLE-fusion proteins as previously described (12,28,31). After cleavage to remove the tags, all constructs retained an extra Gly at the N-terminus of the native sequence. Protein expression was carried out in *E. coli* BL21 (DE3) (Novagen, Darmstadt, Germany) at 37°C in LB medium for unlabeled proteins or in M9 minimal medium supplemented with 1 g L^−1^ of (^15^NH_4_)_2_SO_4_ for uniformly ^15^N-labeled proteins.

### SRCD and OCD sample preparation and measurements

**Figure 1:**
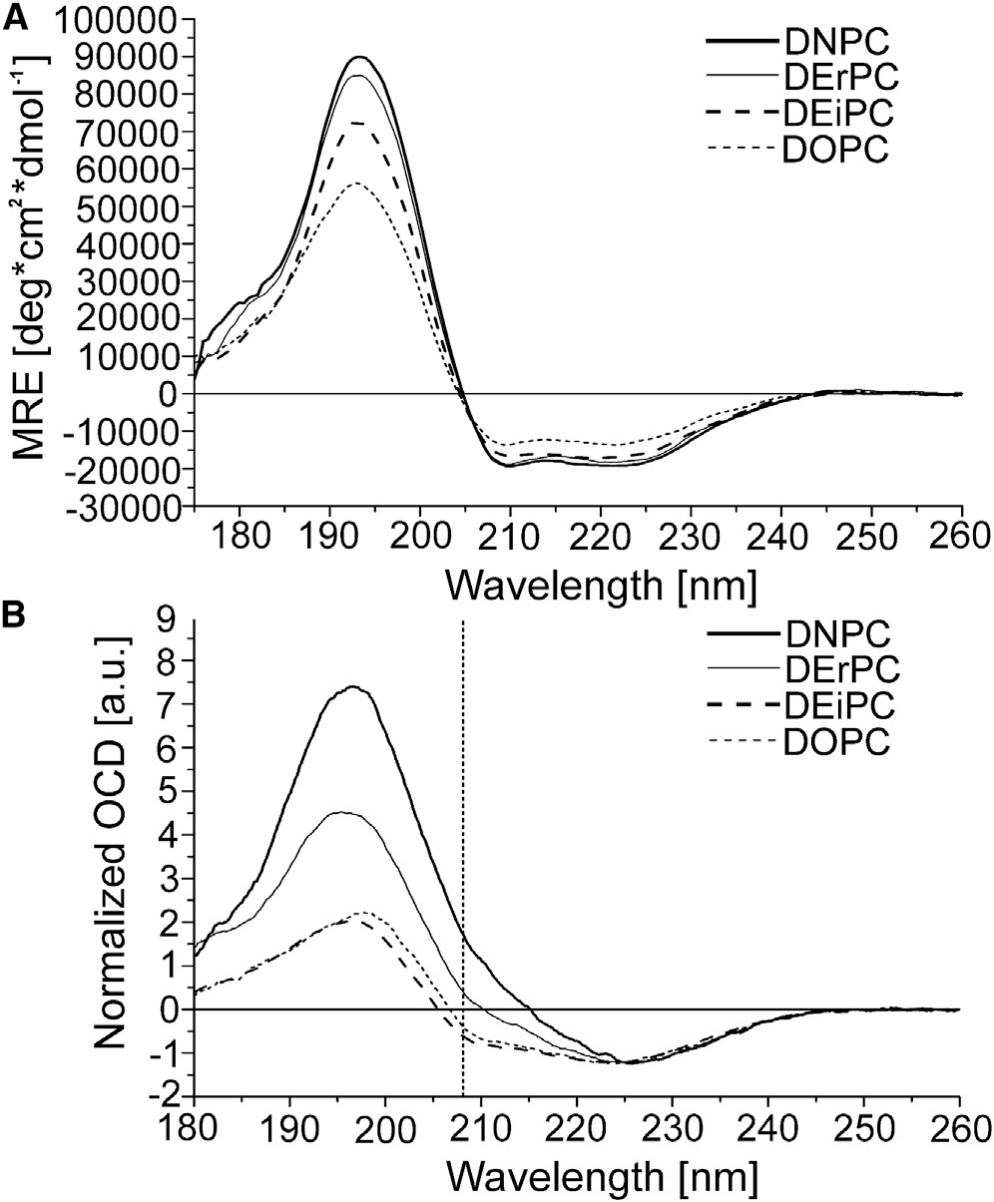
(*A*) SRCD spectra of ΔE5 reconstituted in different lipid vesicles (in isotropic suspension), showing a predominantly *α*-helical secondary structure irrespective of the bilayer thickness. The content of helical aggregates, however, is found to increase with decreasing bilayer thickness, as indicated by the reduction in signal intensities at shorter wavelengths due to absorption flattening and light scattering. (*B*) OCD spectra of ΔE5 reconstituted in macroscopically aligned lipid bilayers of different thickness. Spectra are normalized to the same intensity at 225 nm to illustrate the differences in the characteristic orientation-sensitive band at 208 nm (*dashed line*). ΔE5 has an upright orientation in thick DNPC membranes, as reflected by the positive ellipticity at 208 nm, whereas with decreasing bilayer thickness the ellipticity gradually becomes negative, indicating a more tilted alignment of the helix. A reduction in the overall signal intensity of the positive band is observed from DNPC to DOPC as a result of increased protein aggregation in thinner bilayers.

SRCD and OCD sample preparation, measurements, and spectrum deconvolution were performed as described previously (28,31,41) and are described in detail in the Supporting Materials and Methods. Briefly, ΔE5 was reconstituted in small unilamellar vesicles (SUVs) made of long-chain DNPC (1,2-dinervonoyl-*sn*-glycero-3-phosphocholine; di-C24:1), DErPC (1,2-dierucoyl-*sn*-glycero-3-phosphocholine; di-C22:1), and DEiPC (1,2-dieicosenoyl-*sn*-glycero-3-phosphocholine; di-C20:1), with a protein/lipid ratio of 1:50 (mol/mol), in addition to the more common lipids DOPC (1,2-dioleoyl-*sn*-glycero-3-phosphocholine; di-C18:1), POPC (1-palmitoyl-2-oleoyl-*sn*-glycero-3-phosphocholine; C16:0/C18:1), and DMPC (1,2-dimyristoyl-*sn*-glycero-3-phosphocholine; di-C14:0). All lipids were purchased from Avanti Polar Lipids (Alabaster, AL). SRCD spectra were collected on the UV-CD12 beamline (formerly called the CD12 beamline at Daresbury Laboratory, Warrington, UK) at the ANKA storage ring (Karlsruhe Institute of Technology, Karlsruhe, Germany). The beamline components and its experimental end-station have previously been described in detail (42,43). SRCD spectra were recorded at 20°C for DOPC and DEiPC, at 30°C for DErPC, and at 35°C for DNPC to stay above the lipid phase transition temperature, using a cell holder that was thermostatted by Peltier elements. Secondary structure analysis was performed using the DichroWeb server (44–47). The SRCD spectra shown in Fig. 1 A have been deposited in the Protein Circular Dichroism Data Bank (PCDDB) (48) (http://pcddb.cryst.bbk.ac.uk/home.php) and are publically available under PCDDBID codes CD0004264000, CD0004265000, CD0004266000, and CD0004267000.

For OCD measurements, macroscopically aligned CD samples were prepared from the E5 vesicle suspensions by depositing an aliquot of the lipid suspension onto a quartz glass plate. OCD measurements were performed on a desktop Jasco instrument (Groß-Umstadt, Germany). To reduce possible spectral artifacts caused by a variable quality of protein reconstitution, at least three independent samples were prepared, measured, and finally averaged to get the final OCD spectrum.

### Solid-state NMR sample preparation and measurements

Solid-state NMR sample preparation and measurements were performed as described previously (12,28) and are described in detail in the Supporting Materials and Methods. Briefly, uniformly ^15^N-labeled ΔE5 and PDGFR-TMD were reconstituted together or alone in macroscopically aligned lipid bilayers made of DNPC, DErPC, DEiPC, and DOPC (protein/lipid ratio of 1:50 (mol/mol)). POPC and DMPC were also used, but due to severe protein aggregation, only limited data are shown in the Supporting Material. ^15^N-NMR measurements were carried out on a Bruker Avance 600 MHz spectrometer (Bruker-Biospin, Karlsruhe, Germany) using a custom-built Low-E ^1^HX probe equipped with cross-coil resonators of rectangular cross section (Karlsruhe Institute of Technology Karlsruhe, Germany, and National High Magnetic Field Laboratory, Tallahassee, FL) (49). For the NMR measurements, the temperature was set to 20°C for DOPC and DEiPC, 30°C for DErPC, and 35°C for DNPC samples. The quality of the lipid alignment was checked by measuring ^31^P-NMR spectra using a Hahn echo sequence. For ΔE5, three individual samples for each lipid (derived from three different protein batches) were analyzed.

### Solid-state NMR evaluation of the helix tilt

To determine the helix tilt angle (defined between the membrane normal and the helix axis) and the molecular order parameter *S*_*mol*_ (a qualitative measure of mobility with 0 ≤ |*S*_*mol*_| ≤ 1.0), the lineshapes of the 1D NMR spectra of ΔE5 and PDGFR-TMD were deconvoluted into different fractions as previously described (12). Briefly, each sample was assumed to contain three different protein contributions corresponding to 1) protein in well-oriented bilayers, 2) protein in misaligned bilayers, and 3) protein that was not properly reconstituted and is therefore referred to as aggregated. The ratio of oriented to nonoriented bilayers, as well as the mosaic spread describing the quality of alignment of the oriented bilayer fraction, was obtained independently from the ^31^P-NMR spectrum of each sample. We fitted the experimental ^15^N-NMR spectra with simulated lineshapes that we calculated using these parameters from the ^31^P-NMR data. We performed the simulations by systematically varying the helix tilt angle, the value of *S*_*mol*_, and the fraction of aggregated protein (*f*_*agg*_). The best fit between the experimental spectra and the calculated lineshapes was judged on the basis of the minimal root mean-square deviation (RMSD) over the intensities. The principal values of the ^15^N-CSA tensor were determined from 1D measurements of the ΔE5 protein powder (Fig. S1 *A*) and set to 53.6 ppm, 77.1 ppm, and 218.5 ppm. The truncated ΔE5 peptide was modeled as an ideal *α*-helix for 28 core residues (Trp-5 to Trp-32) plus six randomly structured terminal residues. The detailed results of each analysis are shown in Table S1 and the averaged results are summarized in Table 2. To determine the helix tilt angle of ΔE5 from 2D NMR SAMMY measurements, we fitted the observed spectra with simulated PISA wheels of ideal *α*-helices by varying the tilt angle and *S*_*mol*_ value as previously described (50). An *α*-helical conformation with uniform dihedral angles *Ф* = −61° and *Ψ* = −45° was used for all residues, and the angle between the ^15^N chemical-shift tensor (principal axis corresponding to 218.5 ppm) and the NH vector was 18.5°. A maximum ^1^H-^15^N dipolar coupling corresponding to the peak-to-peak position (half splitting) of 8.8 kHz was determined from a SAMMY spectrum of the ΔE5 protein powder, using the same experimental parameters employed for the reconstituted protein (Fig. S1 *B*).

## Results

### SRCD: conformation of ΔE5 in lipid membranes and influence of bilayer thickness

The common phospholipids DMPC and POPC are widely used as model membranes and are among the most favored lipids for general biophysical analyses. However, all of our initial attempts to incorporate the E5 protein into these standard lipid bilayers failed badly, as they led to severe protein aggregation. In an earlier study, we were able to reconstitute the wild-type and different analogs of E5 into DMPC, but only with the help of lyso-lipid that was added to soften the membrane and make it more tolerant to defects (31). Fortunately, we eventually succeeded in reconstituting E5 in lipid membranes made of much thicker DErPC in the liquid crystalline state (28). It is now clear that the fundamental problem with conventional lipids can be (retrospectively) attributed to a pronounced hydrophobic mismatch between the helix length and the bilayer thickness. Here, we demonstrate that one can routinely reconstitute E5 by using phospholipids with very long acyl chains, resulting in stable samples with well-aligned helices and with negligible aggregation.

To examine the influence of membrane thickness on the secondary structure and aggregation behavior of E5, we selected a series of phospholipids with long unsaturated acyl chains, whose phase transition temperatures are amenable to the experimental conditions of CD and NMR (saturated lipids would have required extremely high temperatures): DNPC (di-C24:1), DErPC (di-C22:1), and DEiPC (di-C20:1). In addition, we used the more common DOPC (di-C18:1), POPC (C16:0/C18:1), and DMPC (di-C14:0). Due to the number of methylene units in the fatty acyl chains, the hydrophobic thickness of the corresponding model membrane increases from 25.6 Å in comparatively thin DMPC bilayers to 26.8 Å in conventional DOPC, 30.6 Å in thicker DEiPC, 34.4 Å in DErPC, and 38.2 Å in very thick DNPC bilayers (calculated according to Marsh (51)).

Generally, when unsaturated lipids are used to prepare SUVs for a CD analysis of membrane-bound proteins, it is virtually impossible to collect CD spectra with a good signal/noise ratio at wavelengths below 200 nm. This is due to the high background absorption of the lipid double bonds as well as to the intrinsic light scattering of the vesicles. SRCD, on the other hand, is ideally suited to tackle this challenge, because the 1000-fold higher photon flux makes background absorption less critical, and hence the spectral quality is greatly enhanced compared with conventional CD. We thus managed to collect high-quality SRCD spectra of the truncated ΔE5 analog reconstituted in SUVs made of several different long-chain lipids. As expected, ΔE5 showed a predominantly helical secondary structure in the tested environments (Fig. 1 *A*). Interestingly, the intensity of the CD bands, and especially that of the 194 nm band, decreased from very thick DNPC to the thinner DOPC bilayers. This apparent change in helix content does not represent a real change in the secondary structure of E5. Instead, it can be explained by an increasing contribution of absorption flattening, and by enhanced light-scattering artifacts, due to the formation of helical protein oligomers and/or aggregates, as previously described for the E5 wild-type protein (28,31). Protein oligomerization and aggregation are known to lead to an inhomogeneous distribution of the chromophores in a sample, which results in less protein signal being detected (52,53).

**Figure 2:**
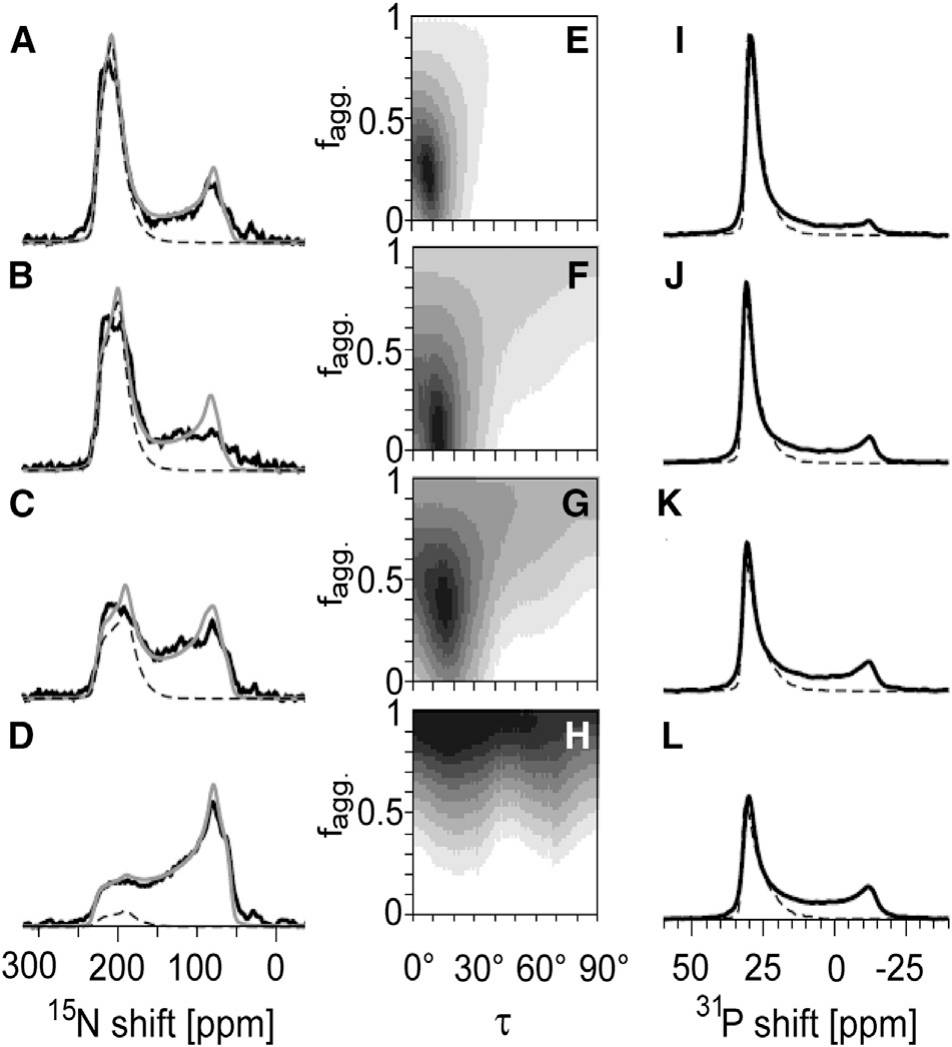
1D solid-state ^15^N- and ^31^P-NMR analyses were used to assess the membrane alignment and aggregation tendency of ΔE5 (protein batch 1 is shown here; see Figs. S3 and S4 for batches 2 and 3). (*A–L*) The uniformly ^15^N-labeled protein was reconstituted in bilayers of different thickness, namely, DNPC (*A*, *E*, and *I*), DErPC (*B*, *F*, and *J*), DEiPC (*C*, *G*, and *K*), and DOPC (*D*, *H*, and *L*) (see Fig. S5 for POPC and DMPC). (*A–D*) To deconvolute the experimental ^15^N-NMR spectra (*solid black lines*), they were fitted with simulated lineshapes representing three fractions: well-oriented peptide, properly reconstituted peptide in misaligned membrane regions, and aggregated peptide. In this way, the helix tilt angle of the well-oriented peptide population (*dashed lines*) could be estimated; the sum of all three contributions is also shown (*solid gray lines*). The agreement between the calculated and experimental spectra as a function of tilt angle and aggregated fraction was judged from RMSD plots (*E–H*; *black* indicates the lowest RMSD value). (*I–L*) In parallel, solid-state ^31^P-NMR of the phospholipids (*solid black lines*) was used to assess the quality of alignment of the lipid matrix. For each sample, the proportion of well-oriented lipid (*dashed gray line*) compared with misaligned membranes was obtained and used to fit the corresponding ^15^N spectra. In this iterative analysis, an increasing tilt angle of the E5 helix (away from the membrane normal) and an increasing amount of aggregated peptide were found with decreasing bilayer thickness upon going from DNPC to DOPC (see also Table 2).

Fig. 1 *A* shows that E5 is well reconstituted in very thick DNPC lipid bilayers, and hardly any aggregation is observed. On the other hand, with decreasing bilayer thickness, more and more protein is seen to aggregate, resulting in a loss of signal intensity as described above. Notably, despite the increasing occurrence of aggregation, the helical lineshapes of the CD spectra indicate that the protein does not unfold during aggregation, but remains mostly helical. Additional signs of protein aggregation were obtained from NMR measurements (see Figs. 2 and 4), and from the increased degree of light scattering in the corresponding UV spectra (Fig. S2). Therefore, the secondary-structure deconvolution of the SRCD data is reliable only for DNPC and DErPC, and not for DEiPC or DOPC. When we analyzed it using the CONTIN algorithm, we found that ΔE5 has a total helix content of ∼91% in DNPC and ∼89% in DErPC, plus minor fractions of *β*-strand, turn, and random coil (Table 1). Notably, ∼73% (corresponding to 26 amino acids) was found to be in an ideal *α*-helical conformation (*α*_*R*_ content) in DNPC. Outside of the membrane, the E5 helix is less regular, as reflected by the *α*_*D*_ content (six amino acids in DNPC), and is further flanked by unstructured C- and N-termini (corresponding to only one or two amino acids at either end of ΔE5). In the thinner membranes of DEiPC and DOPC, the E5 transmembrane segment is too long to match the hydrophobic bilayer core, which results in an increased extent of protein aggregation and/or nonspecific oligomerization.

### OCD: influence of the bilayer thickness on the orientation of the E5 transmembrane helix

For PDGFR, it was recently shown that the membrane thickness affects not only the quality of the reconstituted protein sample but also the actual orientation of the transmembrane helix in the lipid bilayer (12). Since E5 activates PDGFR via helix-helix interactions, it is of fundamental interest now to find out how the E5 helix responds to the bilayer thickness, and to compare this with PDGFR. To address the alignment of ΔE5, we reconstituted it in macroscopically oriented model membranes with varying bilayer thickness. OCD of such samples provides a fast and sensitive way to estimate the tilt angle of a straight *α*-helix in a lipid bilayer (12,28,31,41,54–64). A helix that is aligned parallel to the membrane surface gives rise to a dominant-negative 208 nm band that has a stronger negative intensity than the 222 nm band (65,66). For a tilted helix, on the other hand, the negative 208 nm fingerprint band loses its intensity, and it will reach even positive values for a fully upright transmembrane orientation. When ΔE5 was reconstituted in very thick DNPC, a positive ellipticity at 208 nm was observed, indicating an upright insertion of ΔE5 (Fig. 1 *B*). In moderately thick DErPC bilayers, the band was reduced but still positive, indicating that the protein was slightly tilted. Finally, in thinner DEiPC and DOPC membranes, the 208 nm band became negative, which is a sign that the protein had tilted even more in these membranes. At the same time, a strong reduction in the overall signal intensities was observed, especially at short wavelengths. These artifacts of absorption flattening and light scattering can be attributed to an increasing degree of protein aggregation in these bilayers, as was observed by conventional CD (see above). Clearly, with decreasing bilayer thickness, the E5 protein tries to adapt by tilting, but at the same time an increasing amount of the protein becomes aggregated due to hydrophobic mismatch.

### 1D-NMR: estimation of the membrane alignment and stability of E5

After completing the qualitative OCD assessment, we performed 1D solid-state NMR measurements of uniformly ^15^N-labeled protein samples to determine the orientation of the protein helix more accurately and to quantify the fraction of aggregated material. For this purpose, we reconstituted uniformly ^15^N-labeled ΔE5 in macroscopically aligned lipid bilayers made of the same lipids used in the above SRCD and OCD experiments. For each lipid, we analyzed three individual samples to assess the reproducibility of the effects. Generally, in the solid-state ^15^N-NMR spectra of transmembrane helices, two regions can be distinguished along the chemical shift axis: signals in the downfield region at ∼200 ppm originate from well-aligned transmembrane segments, and the upfield region contains the broader peak of a powder pattern at ∼75 ppm. These upfield signals represent not only the aggregated protein but also any well-reconstituted E5 that happens to be located in a misaligned part of the (nonideal) oriented sample. Within the series of NMR spectra, we see that the peak in the downfield region at 200 ppm shifts to lower ppm values with decreasing membrane thickness and shows a slight increase in linewidth (Figs. 2, S3, and S4). This observation suggests that the protein becomes more tilted within the series from DNPC to DErPC to DEiPC, in full support of the OCD data described above. At the same time, the signal intensity is found to decrease considerably in the downfield region, but increases in the upfield region at ∼75 ppm. In the last lipid of the series, DOPC, essentially a pure powder spectrum is observed, corresponding to complete protein aggregation, as was also the case in POPC and DMPC lipid bilayers (Fig. S5). A similar behavior is seen in two further series of NMR experiments with freshly prepared samples using different batches of protein (Figs. S3 and S4), confirming that the observed effects are reproducible. These data clearly show that the unusually long E5 helix is most stable in very thick DNPC, and it responds to hydrophobic mismatch by adjusting its tilt angle to some extent in moderately thick bilayers. Given the dramatic increase in protein aggregation when approaching standard DOPC, POPC, and DMPC membranes, E5 is obviously unable to compensate for strong mismatch and instead becomes aggregated.

Analogous changes are seen in the corresponding ^31^P-NMR spectra of phospholipids in the same samples. These spectra tend to show two components: well-aligned bilayers giving rise to a narrow signal in the downfield region at ∼30 ppm, and misaligned bilayers producing a powder spectrum with a peak in the upfield region at ∼−15 ppm. To assess their relative contributions, we deconvoluted the two fractions of well-aligned and misaligned membrane domains by lineshape simulations of the ^31^P-NMR spectra. We then used this information to analyze the lineshapes of the more complex ^15^N-NMR spectra. These spectra consist of contributions from 1) well-oriented protein in well-aligned membranes, 2) well-oriented protein in misaligned membrane regions, and 3) aggregated protein that was not properly reconstituted, as explained in more detail in Muhle-Goll et al. (12).

We fitted the ^15^N-NMR data using a variable helix tilt angle *τ* (defined as the angle of the helix axis with respect to the membrane normal), a variable helix order parameter *S*_*mol*_, and a variable degree of protein aggregation, in a manner similar to that described in our previous NMR study of PDGFR-TMD (12). For each different lipid sample, we could thus obtain the tilt angle of ΔE5 and quantify the *f*_*agg*_. This deconvolution showed that the helix tilt angle increased slightly, from ∼12° ± 3° in thick DNPC bilayers to 14° ± 2° in DErPC, to 17° ± 1° in DEiPC, and finally to 21° ± 5° in thin DOPC (Tables 2 and S1; nominal errors were obtained from the averages of the three batches of protein). At the same time, the amount of aggregated protein increased dramatically, from roughly 13% ± 7% in DNPC and 10% ± 3% in DErPC to 35% ± 5% in DEiPC and up to severe aggregation (77% ± 8%) in DOPC. The order parameter *S*_*mol*_ was found to be close to 1.0 in all lipid systems, indicating that the helix did not undergo any wobbling motion.

**Figure 3:**
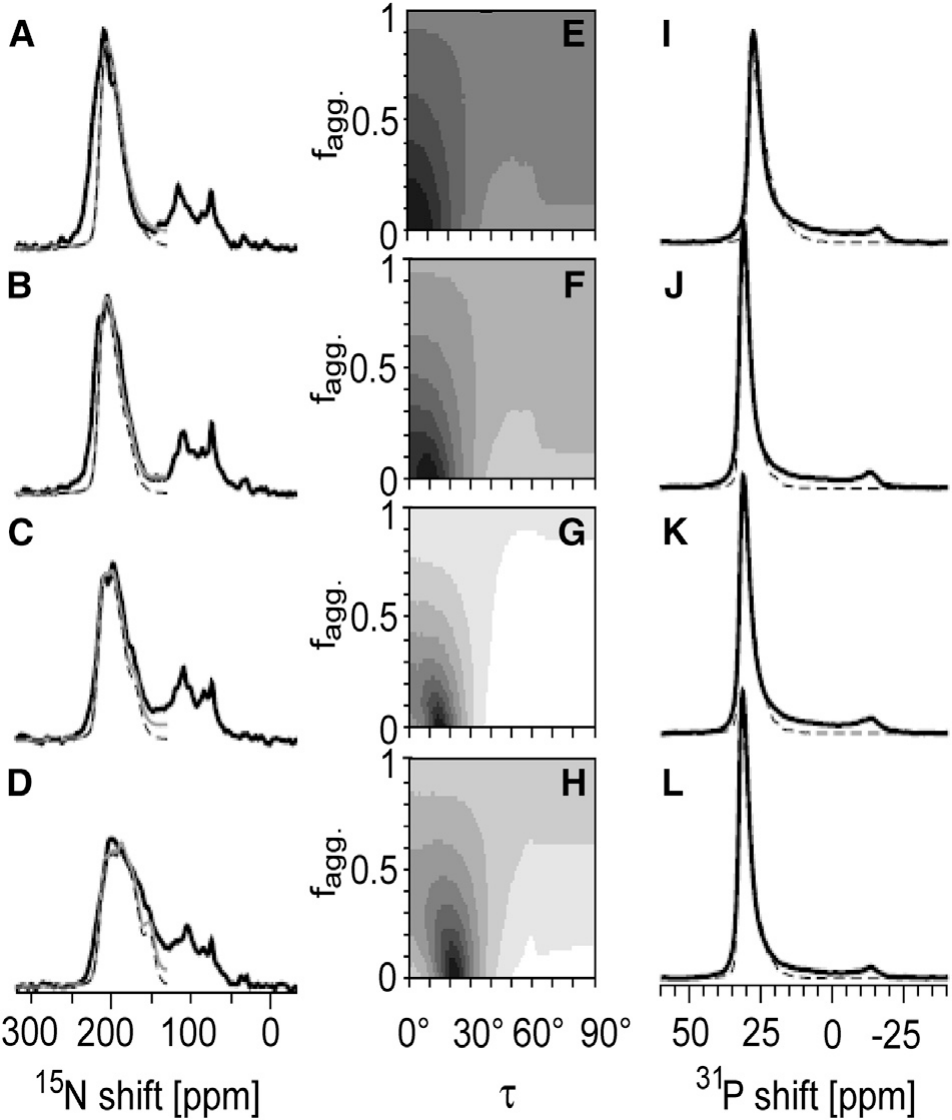
1D solid-state ^15^N- and ^31^P-NMR analyses of the membrane alignment and aggregation tendency of PDGFR-TMD, in a lipid series analogous to that used for ΔE5 (see Fig. 2). (*A–L*) ^15^N-NMR (*A*–*D*) and ^31^P-NMR (*I*–*L*) lineshape analyses were carried out in DNPC (*A*, *E*, and *I*), DErPC (*B*, *F*, and *J*), DEiPC (*C*, *G*, and *K*), and DOPC (*D*, *H*, and *L*). The tilt angle of PDGFR-TMD was found to behave very similarly to that of E5 in all membranes studied here, although the protein had a less pronounced tendency to aggregate (see Table 2).

In a previous study (12), we observed a similar dependence of helix behavior on bilayer thickness in PDGFR-TMD; however, we used a somewhat shorter set of lipids in that study. Here, to compare the tilting behavior of E5 directly with the membrane alignment of PDGFR, we repeated the analysis of its TMD in the same lipid bilayers used for ΔE5, extending our previously published series to longer acyl chains. We found that the corresponding 1D^15^N-NMR spectra of PDGFR-TMD had the same type of signals at ∼200 ppm originating from the well-aligned protein fraction (Fig. 3). The contributions of misaligned and aggregated PDGFR-TMD in the upfield region of the spectrum at ∼75 ppm, however, were obscured by signals of the isotropically averaged termini and by side-chain nitrogens, which are much more abundant in PDGFR (one Arg, three Lys, and one Gln) than in E5 (one Gln). Therefore, the upfield part of the NMR spectra could not be included in this lineshape deconvolution. The tilt angle of PDGFR-TMD determined by this fitting procedure was ∼3° in DNPC, 10° in DErPC, 14° in DEiPC, and 22° in DOPC. These values and especially their trend are remarkably similar to the behavior seen for ΔE5 (Table 2). Compared with ΔE5, however, the PDGFR-TMD suffers less from aggregation, as the upfield ^15^N-NMR signals (Fig. 3) are far less intense than those of E5 (Fig. 2).

### 2D-NMR: determination of the E5 helix tilt angle in bilayers of different thickness

**Figure 4:**
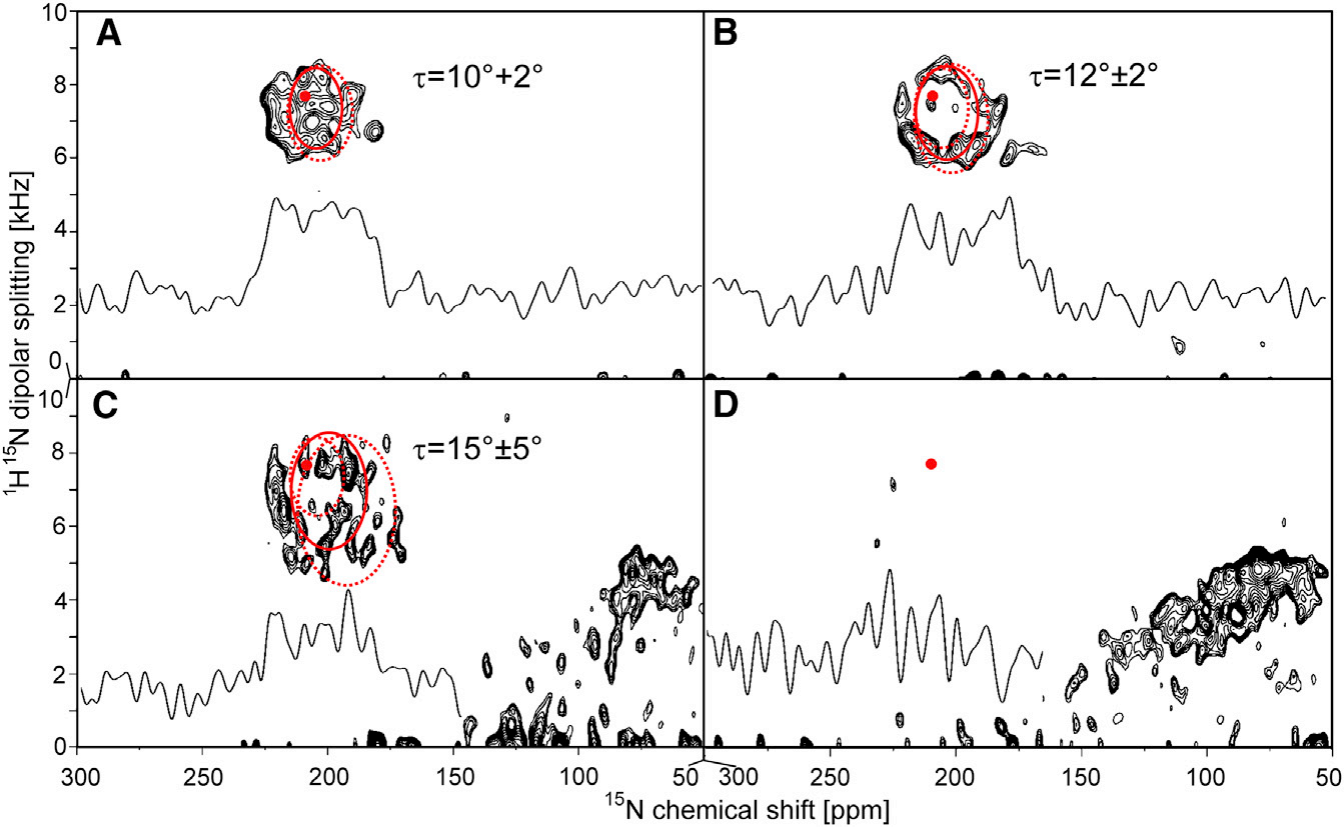
2D solid-state NMR analysis of uniformly ^15^N-labeled ΔE5 in macroscopically aligned membranes of different thickness. Experimental ^1^H-^15^N SAMMY spectra were fitted with simulated PISA wheels (*full circles*) using the experimentally determined ^15^N-CSA tensor, dipolar half splitting, and an order parameter *S*_*mol*_ = 0.97. In each spectrum, the 1D projection of the ^15^N chemical shift (extracted at 7.5 kHz in the ^1^H^15^N dipolar coupling dimension) is shown as a black line to illustrate the signal/noise ratio. (*A–C*) To estimate errors in the tilt-angle determination, PISA wheels that just fit to the observed signals were added (*dotted circles*). The position of the 0° PISA wheel is shown for orientation as a red spot. (*A*) In DNPC, the observed resonance pattern is well reproduced by simulated PISA wheels with tilt angles *τ* between 0° and 10° + 2°. (*B*) In DErPC, the experimental data are fitted best with a simulated PISA wheel of *τ* = 12° ± 2°. (*C*) In DEiPC, PISA wheels with a tilt angle of *τ* = 15° ± 5° cover the observed signals, suggesting a slight kink or a bent helix. (*D*) In DOPC, the protein could not be reconstituted and gave a broad powder distribution of signals.

2D solid-state NMR spectra can provide further resolution and additional spatial information regarding the secondary structure and orientation of membrane proteins. In separated local field experiments, helical proteins give rise to distinctive circular resonance patterns (so-called PISA wheels) that resemble their helical wheel projections (35–37). Most importantly, the position, size, and shape of a PISA wheel convey information about the orientation (tilt angle *τ* and azimuthal rotation angle *ρ*) of the aligned helix. Fig. 4 shows the ^1^H-^15^N SAMMY spectra of uniformly ^15^N-labeled ΔE5 (using the same samples employed for the 1D measurements in Fig. 2) reconstituted in macroscopically aligned membranes of different thickness. In the very thick DNPC bilayers, a group of resonances is seen at ∼180–230 ppm ^15^N-CSA and 6–8.5 kHz in the dipolar coupling dimension (Fig. 4 *A*). The small area in which all resonances overlap is indicative of an almost upright transmembrane orientation of the helix, in good agreement with the OCD and 1D-NMR data described above. In moderately thick DErPC bilayers, the resonances are spread into a proper wheel-like pattern at lower ppm and kHz values, revealing a more tilted orientation of the E5 helix (Fig. 4 *B*). In DEiPC, the coherence of the wheel is lost, and the signals are spread out over several overlapping wheel-like patterns to even lower ppm and kHz values. At the same time, a new group of resonances emerges at ∼50–150 ppm ^15^N-CSA and 2–5 kHz dipolar coupling, representing the increasing fraction of aggregated E5 (Fig. 4 *C*). The spectrum in DOPC consists essentially of a powder spectrum caused by severe protein aggregation due to pronounced hydrophobic mismatch in these conventional lipid bilayers (Fig. 4 *D*).

The tilt angle of ΔE5 in the different lipid systems can be deduced by fitting the experimental spectra with simulated PISA wheels (50,67). In Fig. 4, *A–C*, the best-fit PISA wheels (calculated for an ideal *α*-helix) are shown superimposed on the experimental data. For ΔE5 in DNPC, any PISA wheel with a tilt angle *τ* between 0° and 10° + 2° matches the observed resonances well (Table 2). Due to signal overlap, it is not possible to determine the tilt angle more exactly. For DErPC, a simulated PISA wheel with a tilt angle of 12° ± 2° fits the observed NMR data very well, in full agreement with the 1D-NMR data. In DEiPC, the assignment of a single, uniform PISA wheel is hampered by the broad distribution of resonances. However, PISA wheels with tilt angles covering a range from 10° to 20° match the observed signals well, which is indicative of some helix distortion such as bending or a kink. In DOPC, a PISA wheel analysis does not make sense due to the lack of properly reconstituted protein. The order parameters obtained from fitting the series of SAMMY spectra support those determined from the 1D-NMR measurements. Overall, the 2D-NMR measurements contain more information and are more accurate, and fully confirm the results of the 1D-NMR analyses. The structural responses of E5 are summarized in Fig. 6, which illustrates how E5 adapts to an increasing hydrophobic mismatch: upon going from DNPC to DOPC, the helix adjusts its tilt angle, then suffers from some distortion, and finally loses its membrane alignment completely and forms helical aggregates.

### 1D-NMR: orientation of PDGFR and E5 in a heterooligomeric complex

**Figure 5:**
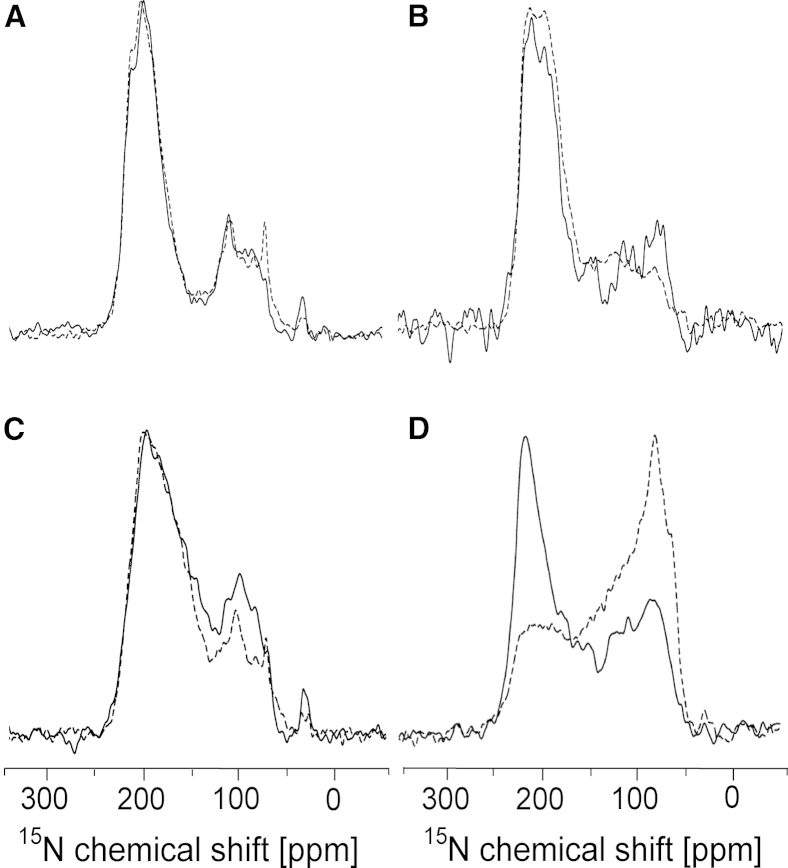
(*A–D*) 1D solid-state ^15^N-NMR analysis of the membrane alignment of PDGFR-TMD and ΔE5 under the influence of each other in thick DErPC (*A* and *B*) and thin DOPC (*C* and *D*) lipid bilayers. The spectra of the hetero mixtures (*solid lines*) are superimposed with the corresponding spectra of the pure ^15^N-labeled ΔE5 and PDGFR-TMD (*dashed lines*), taken from Figs. 2 and 3, respectively. (*A* and *C*) For the ^15^N-PDGFR-TMD/^14^N-ΔE5 complex, no changes compared with the 1D spectra of the pure ^15^N-PDGFR-TMD were observed, indicating that the alignment of the protein is not affected by the presence of ΔE5. (*B* and *D*) For the ^15^N-ΔE5/^14^N-PDGFR-TMD complex, no changes were observed in DErPC; however, in DOPC, the presence of PDGFR-TMD promoted the reconstitution and induced a transmembrane orientation of ΔE5.

Next, we determined the alignment of both E5 and PDGFR in a biologically relevant heterooligomeric complex to examine their influence on each other. We reconstituted both proteins together in lipid membranes using an equimolar ratio of labeled ^15^N-PDGFR-TMD and unlabeled ^14^N-ΔE5, and, in a reverse experiment, some labeled ^15^N-ΔE5 and unlabeled ^14^N-PDGFR-TMD. These ^15^N/^14^N mixtures were reconstituted in thick DErPC and thin DOPC bilayers, and the orientation of either protein was determined by solid-state 1D-NMR. When ^15^N-PDGFR-TMD and ^14^N-ΔE5 were reconstituted together in thick DErPC bilayers, the observed 1D-NMR spectrum perfectly matched the spectrum of pure PDGFR-TMD (Figs. 5 *A* and S6), indicating that the orientation of PDGFR does not change due to the presence of E5. Accordingly, spectral deconvolution yielded a tilt angle of 12° ± 2°, which is similar to the tilt found for PDGFR-TMD alone (Tables 3 and S2). In the reverse experiment, when ^15^N-ΔE5 was reconstituted together with ^14^N-PDGFR-TMD in DErPC, the lineshape was the same as that observed for the single protein alone (Figs. 5 *B* and S7), indicating that the transmembrane orientation of ΔE5 in thick membranes is not affected by the presence of PDGFR. Here, a tilt angle of 14° ± 0° was found, which is close to the tilt measured for the pure protein in the same lipid bilayer. When ^15^N-PDGFR-TMD and ^14^N-ΔE5 were reconstituted in thin DOPC membranes, the slightly tilted orientation of the protein was not affected by the presence of ΔE5 either (Figs. 5 *C* and S8), as the tilt angle of 23° ± 1° is the same as that observed for the PDGFR-TMD alone. Most interestingly, however, the 1D spectrum of the ^15^N-ΔE5/^14^N-PDGFR-TMD complex in DOPC reveals a complete change in the lineshape compared with the spectrum of pure ΔE5 (Figs. 5 *D* and S9). Now, a signal originating from well-reconstituted E5 in a proper transmembrane alignment is seen in the downfield part of the spectrum, whereas E5 alone in DOPC had been mostly aggregated (Fig. 2 *D*). Obviously, the instability of E5 due to hydrophobic mismatch in these comparatively thin bilayers was reversed by the presence of PDGFR. The receptor clearly promoted the successful reconstitution of E5 and stabilized its proper transmembrane alignment with a tilt angle of 7° ± 1°. These data suggest that PDGFR and E5 actively assemble into a heterooligomeric complex in DOPC membranes, allowing ΔE5 to overcome the pronounced hydrophobic mismatch by binding to PDGFR and adopting an upright orientation.

## Discussion

The E5 oncoprotein, with a length of only 44 amino acids, is an integral membrane protein with remarkable features. It is mostly hydrophobic, but is highly specific in that it can activate only PDGFR *β*, and no other closely related RTKs (17,18,68). This point is even more astonishing in the light of numerous reports that the specific sequence is not critical for the biological activity of E5, as most of the hydrophobic amino acids can be conservatively substituted without any loss of function (69–72). Hence, features other than the actual sequence must play a decisive role in the recognition of PDGFR, such as the presence and position of two polar key residues (Gln-17 in the TMD and Asp-33 in the juxtamembrane region), the hydrophobic dimer interface on the E5 helix that drives its self-assembly, and the actual length of the TMD and/or its orientation in the lipid membrane. The lipid bilayer itself may also have an influence on the E5 protein and its ability to interact with itself and with PDGFR. Here, we found that the conformation, orientation, and stability of the E5 protein are indeed affected by the lipid bilayer and by the presence of PDGFR.

Using solid-state NMR, SRCD, and OCD, we found that ΔE5 can be stably incorporated into very thick lipid bilayers (e.g., DNPC), but in conventional membranes (e.g., DOPC) it aggregates severely due to hydrophobic mismatch. When inserted into thick planar lipid membranes, the protein adopts the same *α*-helical conformation that has previously been observed in detergent micelles and DMPC bilayers (22,24,25,28). For ΔE5, the total helix content of 91% in DNPC and 89% in DErPC is slightly higher than that in detergent micelles (82%) (28), indicating that an appropriate lipid environment stabilizes the transmembrane helix by roughly one additional turn. Notably, ∼73% of ΔE5 was found to be in an ideal helix conformation (*α*_*R*_ content) in DNPC. This percentage is equivalent to a stretch of ∼26 amino acids, which fits remarkably well with the length of the hydrophobic sequence flanked by Trp-5 and Trp-32. Tryptophan and to a lesser extent other aromatic side chains are typically located in the amphiphilic interface regions of transmembrane segments, where they serve as anchors to hold the membrane proteins at a well-defined position within the lipid bilayer (67,73–77). In E5, there are additional anchoring residues (Phe-6 and Tyr-31) right next to the two prominent tryptophans at either end of the TMD, which contribute to a strong anchoring effect on each face of the bilayer. The E5 helix also extends beyond the membrane, though with a less regular conformation, as reflected by the distorted helix content *α*D, and the C- and N-termini are unstructured.

**Figure 6:**
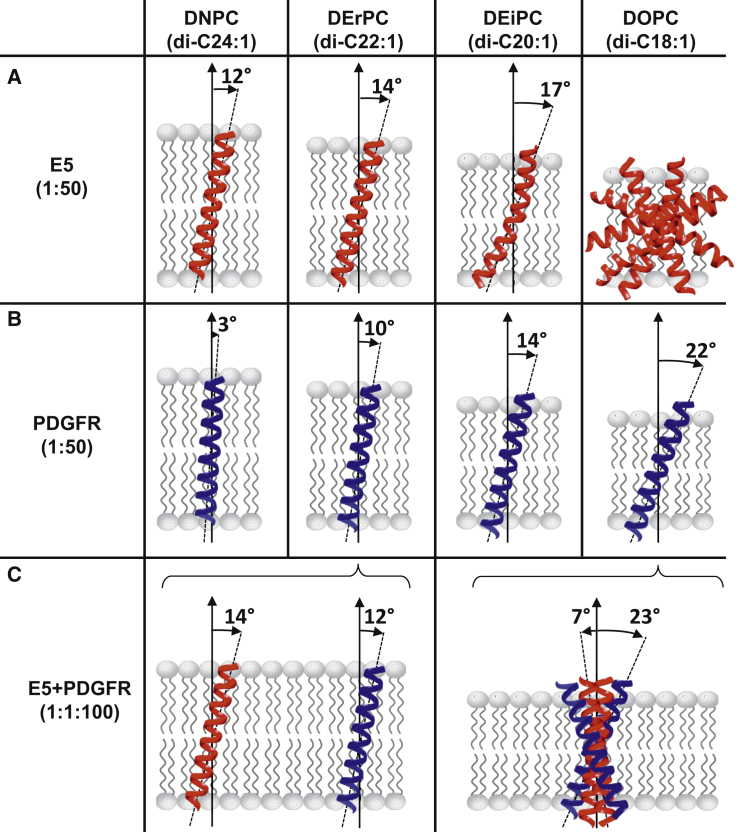
(*A–C*) Model of the orientational behavior of the E5 protein (*A*), the TMD of PDGFR (*B*), and the PDGFR/E5 heterooligomeric complex (*C*) in lipid bilayers of different thickness, reconstituted with peptide/lipid ratios as indicated. (*A*) Upon going from very thick DNPC membranes to common bilayers (such as DOPC, POPC, and DMPC), the unusually long E5 helix has to adapt to the increasing hydrophobic mismatch. While being oriented essentially upright (in DNPC), it first adjusts its tilt angle (in DErPC), then suffers from helix distortion (in DEiPC), and finally loses its membrane alignment and forms helical aggregates (in DOPC). (*B*) The TMD of PDGFR adjusts its tilt angle in response to the bilayer thickness. (*C*) The E5 protein can compensate for the hydrophobic mismatch in thin DOPC bilayers (such as the membranes of the Golgi compartment) by binding to the TMD of the monomeric receptor, thereby clustering the receptor in its active conformation. In thick DErPC bilayers (such as in the plasma membrane), both proteins remain largely on their own. Here, the hydrophobic mismatch is less critical, and thus E5 can reside stably in the bilayer without binding to the receptor. Molecular graphics were performed with the UCSF Chimera package (89).

Clearly, the TMD in E5 with 28 residues (24 amino acids + 4 anchoring ones) is much longer than the typical stretch of ∼20 hydrophobic amino acids that is commonly expected for single-span integral membrane proteins (78). PDGFR also has a remarkably long membrane-spanning domain of 27 residues (25 amino acids + 2 anchoring ones) (12), and our ^15^N-NMR analyses yielded comparable helix tilt angles for both ΔE5 and the TMD of the receptor. Thus, it is tempting to speculate that E5 has evolved into a 3D structure that is perfectly matched to the length and orientation of the PDGFR transmembrane segment. A recent study showed that randomized TMDs can also activate PDGFR, although they do not contain any sequences derived from the E5 protein (79). Interestingly, the common feature that these peptides share with the E5 protein is an unusually long hydrophobic stretch of 25 amino acids, indicating that indeed the extreme length of the E5 TMD is critical for interaction with PDGFR. In this way, the key residue in the middle of the E5 helix, Gln-17, will always be positioned at the right depth to interact with the central threonine on PDGFR. The second key residue on E5, Asp-33, lies in the region where the helix starts to unwind; therefore, it is not necessarily located on the opposite face of the helix relative to Gln-17, as was previously proposed for the *cis* model of the E5/PDGFR complex (22). Here, we have demonstrated that the orientation of E5 is sensitive to the bilayer thickness, as was previously observed for PDGFR. In very thick DNPC membranes, both proteins are inserted almost upright, as the hydrophobic length of the transmembrane segments matches the hydrophobic thickness of the bilayer (Fig. 6). However, in thinner membranes, E5 is under considerable conformational strain when it tries to adapt to the reduced bilayer thickness. Helix tilting in DErPC is seen from the shift of the PISA wheel in the SAMMY spectrum. Some additional helix bending or kink formation is observed in DEiPC, and in DOPC the PISA wheel has totally disintegrated. PISA wheel measurements are very sensitive to variations of the backbone *Ф*, *Ψ* torsion angles, and even minor changes have a strong impact on the pattern of the PISA wheel. For example, previous spectral simulations of an 18-residue helical peptide showed a distinct PISA wheel when standard torsion angles were used, but the pattern essentially disappeared when the torsion angles were varied (67). Interestingly, the helix tilt angles observed for ΔE5 in DErPC, DEiPC, and DOPC are significantly smaller than one would expect based on geometrical arguments. To completely immerse the E5 TMD within the membrane, one would expect to find a tilt angle of 28° in DErPC (instead of the observed 14°), 38° in DEiPC (observed 17°), and 47° in DOPC (observed 21°). Thus, E5 can tilt to a certain extent, but then the protein aggregates because it is unable to tilt any more. Therefore, the observed moderate tilting of E5 does not fully compensate for the hydrophobic mismatch experienced by the protein: there remains a nominal mismatch for the actual length of the TMD of 3.4 Å in DErPC, 6.8 Å in DEiPC, and 9.6 Å in DOPC. Interestingly, in a considerably thinner DMPC bilayer, a maximum helix tilt of only ∼20° was previously reported by infrared measurements (22). On the other hand, an ideal helix of 26 amino acids for the TMD of E5 corresponds to a length of ∼39 Å, which is well matched to the hydrophobic thickness of 38.2 Å of DNPC. Accordingly, the observed tilt angle of 12° in DNPC fits the expected value.

The finding that ΔE5 is not able to compensate for hydrophobic mismatch by tilting is a strong argument for the presence of homodimers (and higher oligomers), despite the fact that the truncated ΔE5 does not contain any cysteines. A single transmembrane segment would be much more sensitive to the bilayer thickness than a dimer or an oligomeric bundle, as has been documented for various monomeric transmembrane helices (39,80–83). Interestingly, even when we diluted the protein in the membrane to promote disassembly of the noncovalent dimer, we observed no changes in the 1D-NMR spectra for either thick or thin membranes (Fig. S10). We may thus conclude that helix-helix interactions lock ΔE5 together as a homodimer, thereby enforcing a relative upright orientation of the bundle. A similar orientational behavior was previously reported for the tetrameric M2 proton channel (84) and for the diacylglycerol kinase, a three-TM-helix membrane protein (67), in which specific electrostatic interactions lock the protein into a more rigid conformation so that it does not respond to changes in the bilayer thickness. Given that the E5 TMD is known to self-assemble per se in lipid membranes (28,85), the corresponding helix-helix interactions must be quite strong and specific to hold the dimer together and force the protein into a more upright orientation. Besides the hydrophobic interface, in the full-length wild-type the two cysteine residues would contribute by forming a covalent link. The biological function of the E5 protein obviously relies on dimerization to maintain a specific conformation in the membrane, enabling it to interact with PDGFR and other cellular targets.

The solid-state ^15^N-NMR analyses presented here clearly demonstrate that the structural behavior of E5 in membranes is sensitive not only to the bilayer thickness but also to the presence of PDGFR. When both proteins were reconstituted by themselves in comparatively thin DOPC bilayers, PDGFR-TMD was well reconstituted, whereas ΔE5 showed severe protein aggregation due to hydrophobic mismatch and its inability to tilt. On the other hand, when the two proteins were reconstituted together, the presence of PDGFR greatly stabilized ΔE5, resulting in a well-oriented alignment of the protein. A recent study found that PDGFR-TMD is already present as preformed dimers in thick lipid membranes, but in thinner membranes, where the protein has to be more tilted, these dimers tend to disassemble (12). We thus conclude that in thin bilayers, E5 binds to the tilted and monomeric TMD of PDGFR to counteract the hydrophobic mismatch (Fig. 6). The need to avoid hydrophobic mismatch may thus be the underlying driving force for the long-range recognition and binding of E5 to the receptor. Once the proteins have approached each other, specific polar and hydrogen (H)-bonded interactions will determine their precise docking in the short range.

In living cells, E5 is known to be localized in the thin membranes of the endoplasmic reticulum (ER) and *cis*-Golgi compartment, where it interacts with the immature and mature forms of PDGFR, whereas only low amounts are detected in the plasma membrane (29,30,86,87). Our data can now explain why and how the intimate interaction of E5 with PDGFR can lead to retention of the complex in the thinner membranes of the ER and Golgi, as the pronounced hydrophobic mismatch becomes less unfavorable for the heterooligomeric bundle compared with the single proteins per se. In this way, it is likely that the subunits of the receptor are clustered by E5 into a functional signal transduction complex in the thin membranes of the ER and Golgi, but when PDGFR is on its own, premature receptor dimerization and activation are prevented by the pronounced hydrophobic mismatch. On the other hand, in thick bilayers (such as in the plasma membrane), no changes were observed in the NMR spectra of the complex compared with the pure proteins, indicating that both proteins remained largely on their own. Here, the hydrophobic mismatch is less critical, and E5 can thus reside stably in the bilayer without binding to the receptor.

In the current mechanistic models for signaling, RTKs are activated upon ligand binding, which results in receptor dimerization followed by activation of the tyrosine kinase domains. Over the last decade, however, this simple model has been challenged by reports that the TMDs of RTKs play a critical role in regulating dimerization. Many RTKs were found to be present as preformed dimers even before ligand binding, such as in the case of PDGFR (12), the EGF receptor (8), the Neu receptor (11), and the Erythropoietin receptor (9,10). However, these preformed dimers are supposed to represent an inactive state in which the subunits are oriented nonfunctionally with respect to one another (see previous studies (6,9,88) for a model of receptor activation). Ligand binding is then supposed to induce a rotation of the subunits toward their active conformation, which brings the catalytic domains into close proximity to allow transphosphorylation and receptor activation. A rotational repositioning of the receptor subunits has also been shown for PDGFR, suggesting that the receptor is active only when the monomers face each other in a specific conformation (11). Together, these findings highlight the role of the TMD as a molecular switch in the activation of PDGFR. It is tempting to speculate that the E5 protein provides a favorable scaffold within the otherwise unfavorable membrane environment of the Golgi compartment, which is beneficial for receptor clustering and for rotational repositioning of the transmembrane segments into their active dimer conformation. In this way, PDGFR can become activated already in the Golgi compartment and engage in signaling without ever reaching the cell surface or encountering a growth factor.

## Author Contributions

D.W. coordinated the project, performed SRCD/OCD measurements, and wrote the manuscript. C.Z. and M.Z. performed NMR measurements. S.L.G. analyzed the NMR data. J.B. analyzed the SRCD/OCD data. P.L.G. designed and developed the NMR equipment used in this study. A.S.U. served as the scientific coordinator and corrected the manuscript.

## Figures and Tables

**Table 1:** Secondary Structure of ΔE5 in Lipid Bilayers

Lipid	*α*_*R*_ (%)	*α*_*D*_ (%)	Sum *α*-Helix (%)	*β*-Strand (%)	*β*-Turn (%)	Unstructured (%)	NRMSD
DNPC	73 (26)	18 (6)	91 (32)	5 (2)	3 (1)	1 (0)	0.044
DErPC	70 (24)	19 (7)	89 (31)	5 (2)	3 (1)	4 (1)	0.043

Deconvolution results of ΔE5 SRCD spectra using the CONTIN algorithm. The secondary-structure contents are given in % and the corresponding numbers of amino acids are shown in brackets. *α*_R,_ regular helix; *α*_D_, distorted helix; NRMSD, normalized RMSD.

**Table 2:** Orientation of ΔE5 and PDGFR-TMD in Lipid Bilayers

Protein	Measurement	Parameter	DNPC	DErPC	DEiPC	DOPC
ΔE5	1D	*τ*_*obs*_ (°)	12 ± 3	14 ± 2	17 ± 1	21 ± 5
		*f*_*agg*_ (%)	13 ± 7	10 ± 3	35 ± 5	77 ± 8
		*S*_*mol*_	0.95 ± 0.04	0.97 ± 0.02	0.95 ± 0.03	0.96 ± 0.04
	2D	*τ*_*obs*_ (°)	0–10 + 2	12 ± 2	15 ± 5	n.d.
		*S*_*mol*_	0.97	0.97	0.97	n.d.
PDGFR-TMD	1D	*τ*_*obs*_ (°)	3	10	14	22
		*f*_*agg*_ (%)	2^a^	0^a^	0^a^	0^a^
		*S*_*mol*_	1	1	1	1

The helix tilt angle (*τ*_*obs*_, defined with respect to the membrane normal), the fraction of aggregated protein (*f*_*agg*_), and the order parameter (*S*_*mol*_) were estimated from 1D- and 2D-NMR spectra. For ΔE5, the averaged results of three individual samples per lipid are shown (see Table S1 for detailed results). n.d., not determined.

a For PDGFR-TMD, the *f*_*agg*_ is less reliable (see text and Fig. 3).

**Table 3:** Orientation of ΔE5 and PDGFR-TMD in the Heterooligomeric Complex

Mixture	Parameter	DErPC	DOPC
^15^N-ΔE5/^14^N-PDGFR-TMD (1:1)	*τ*_*obs*_ (°)	14 ± 0	7 ± 1
	*f*_*agg*_ (%)	3 ± 3	3 ± 5
	*S*_*mol*_	0.91 ± 0.01	0.97 ± 0.03
^15^N-PDGFR-TMD/^14^N-ΔE5 (1:1)	*τ*_*obs*_ (°)	12 ± 2	23 ± 1
	*f*_*agg*_ (%)	0^a^	0^a^
	*S*_*mol*_	1	1

Averaged results per lipid are shown (see Tables 2 and S2 for details).

a For PDGFR-TMD, the *f*_*agg*_ is less reliable (see text).

## Supporting Citations

References (90–94) appear in the Supporting Material.

## Supporting Material

Document S1. Supporting Materials and Methods, ten figures, and two tables

Document S2. Article plus Supporting Material
