# Supplementary material for: Hydrophobic Mismatch Drives the Interaction of E5 with the Transmembrane Segment of PDGF Receptor
Source: Biophys J. 2015 Aug 18;109(4):737–49. doi: 10.1016/j.bpj.2015.07.022 (PMC4547410; doi:10.1016/j.bpj.2015.07.022)
Supplement: Document S1. Supporting Materials and Methods, ten figures, and two tables [file mmc1.pdf]

SUPPORTING MATERIAL

Hydrophobic mismatch drives the interaction of E5 with the transmembrane segment of PDGF receptor

Dirk Windisch,[1] Colin Ziegler,[2] Stephan L. Grage,[1] Jochen Bürck,[1] Marcel Zeitler,[2] Peter L. Gor'kov,[3] and Anne S. Ulrich[1,2,]*

[1]Institute of Biological Interfaces (IBG-2) and [2]Institute of Organic Chemistry, Karlsruhe Institute of Technology, Karlsruhe, Germany; and [3]National High Magnetic Field Laboratory, Tallahassee, Florida

* correspondence to: anne.ulrich@kit.edu

SUPPORTING MATERIAL AND METHODS

SRCD and OCD sample preparation and measurements

To reconstitute the truncated ΔE5 in liposomes for circular dichroism analysis, the lyophilized protein powder and the lipids were each dissolved in dichloromethane/methanol (50/50, v/v), and insoluble parts were removed by centrifugation. An aliquot of the lipid stock solution was mixed with an aliquot of the ΔE5 stock solution to reach the desired concentrations for a nominal protein-to-lipid ratio of 1:50 (mol/mol). The solvents were evaporated by a stream of nitrogen, followed by subsequent lyophilization to remove residual organic solvent. The protein/lipid mixtures were rehydrated with water to obtain a protein concentration of ~1 mg ml, then thoroughly sonicated and additionally subjected to several freeze-thaw cycles. Afterwards, small unilamellar vesicles were formed by sonication of the lipidic suspension for 4 min in a strong ultrasonic bath. The sonication procedure was repeated 3 times. In between, the water of the ultrasonic bath was cooled down to room temperature with ice, to avoid overheating of the samples. Vesicles samples were always kept above the lipid phase transition before measurement. The synchrotron radiation circular dichroism (SRCD) spectra were collected on the UV-CD12 beamline at the ANKA storage ring (KIT, Germany). The beamline components and its experimental end-station have been previously described in detail (1,2).

For an SRCD measurement, a 3.5 µl aliquot (containing 1 mg ml^{-1} protein) of the sample was filled into a "Birkbeck-type" demountable CaF_2 cell (3). The optical path length of this cell was determined by interferometry to be 13.1 µm. Spectra between 260 and 175 nm were recorded with a scan-rate of 15 nm min^{-1} at 0.5 nm intervals, using a 1 nm spectral bandwidth, a 0.3 s lock-in time constant, a 1.5 s dwell time and three scans were averaged. For baseline correction the protein-free lipidic matrix was measured under the same conditions and subtracted from the protein spectrum to get the final spectrum. Secondary structure analysis was performed using the CONTIN algorithm provided by the DichroWeb server (4–7). Set 7 containing 48 reference spectra of proteins with well-established structures was used as a reference set for the secondary structure deconvolution (8). The quality of the fit between the experimental and back-calculated spectra corresponding to the derived secondary structure was assessed from the normalized root mean-square deviation (NRMSD), with a value of

<0.1 considered a good fit. To calculate the mean residue ellipticities that are used for secondary structure estimation, the concentration of the ΔE5 protein was determined based on the absorbance of the protein at 280 nm, using a molar extinction coefficient of 12490 L mol^{-1} cm^{-1} (9).

For oriented circular dichroism (OCD) measurements, macroscopically aligned membrane samples were prepared from the E5 vesicle suspensions above, by depositing an aliquot of the lipid suspension (containing 3 µg protein and 40-50 µg lipid) onto a quartz glass plate with a 20 mm diameter. The suspensions were allowed to dry, and samples were subsequently equilibrated under 96% relative humidity for 15 h above the lipid phase transition temperature (20°C for DOPC and DEiPC, 30°C for DErPC, 35°C for DNPC) in a sample cell for OCD measurements. This in-house built OCD cell can be integrated in a J-810 spectropolarimeter (Jasco, Groß-Umstadt, Germany) as an accessory. To reduce possible spectral artifacts caused by a variable quality of protein reconstitution, at least three independent samples were prepared, measured and finally averaged to produce the final OCD spectrum.

Solid-state NMR sample preparation and measurements:

For solid-state NMR measurements, uniformly ^{15}N-labeled ΔE5 and PDGFR-TMD were reconstituted in macroscopically aligned membranes made of DNPC, DErPC, DEiPC, DOPC, POPC and DMPC (protein-to-lipid ratio of 1:50 (mol/mol), about 2 mg of protein per sample). As for SRCD, the lyophilized protein powder and lipids were dissolved in dichloromethane/methanol (50/50, v/v) and insoluble parts were removed by centrifugation. The protein/lipid solution was spotted onto 20 glass plates (7.5 x 12 x 0.06 mm, Marienfeld GmbH & Co.KG, Germany), and the samples were allowed to dry followed by drying under vacuum overnight to remove residual organic solvent. The glass plates were then stacked and placed under a humid atmosphere (saturated K_2SO_4) for rehydration at 48°C overnight. Finally, the glass plate stacks were wrapped into parafilm and polyethylene foil to prevent drying during the NMR measurement. Solid-state ^{15}N-NMR measurements were carried out on a Bruker Avance 600 MHz spectrometer (Bruker-Biospin, Karlsruhe, Germany), using a custom-built Low-E ^{1}HX probe equipped with cross-coil resonators of rectangular cross section (KIT Karlsruhe, Germany and NHMFL Tallahassee, USA) (10). For the NMR measurements the temperature was set to 20°C for DOPC and DEiPC, 30°C for DErPC, and 35°C for DNPC samples in order to stay above the lipid phase transition temperature. The quality of the lipid alignment was checked by measuring ^{31}P-NMR spectra using a Hahn echo sequence. One-dimensional (1D) ^{15}N-NMR spectra were obtained using a CP-MOIST pulse sequence with an additional echo pulse (20 µs echo delay), with 10 ms acquisition time, 0.5 ms contact time and 50 kHz ^{1}H-decoupling (11). 25600 scans were acquired with a recycle delay time of 3 s.

For the two-dimensional separated local field measurements, the SAMMY experiment was used, with the optimized SAMPI4 pulse sequence and a 90° pulse of 4.9 µs in the SAMPI4 multi-pulse train, 10 ms acquisition time, 0.5 ms contact time, 50 kHz ^{1}H-decoupling and a recycle delay of 3 s (12,13). 1780 scans with 72 t_1 increments were acquired. A ^{1}H carrier frequency of 9 ppm and a ^{15}N carrier frequency of 180 ppm were used, which are optimal for transmembrane helices. The ^{15}N chemical shifts were referenced to an external sample of solid $(^{15}NH_4)_2SO_4$, which was set to 26.8 ppm corresponding to 0 ppm for liquid ammonia. The dipolar dimension was scaled as previously described (13).

SUPPORTING FIGURES

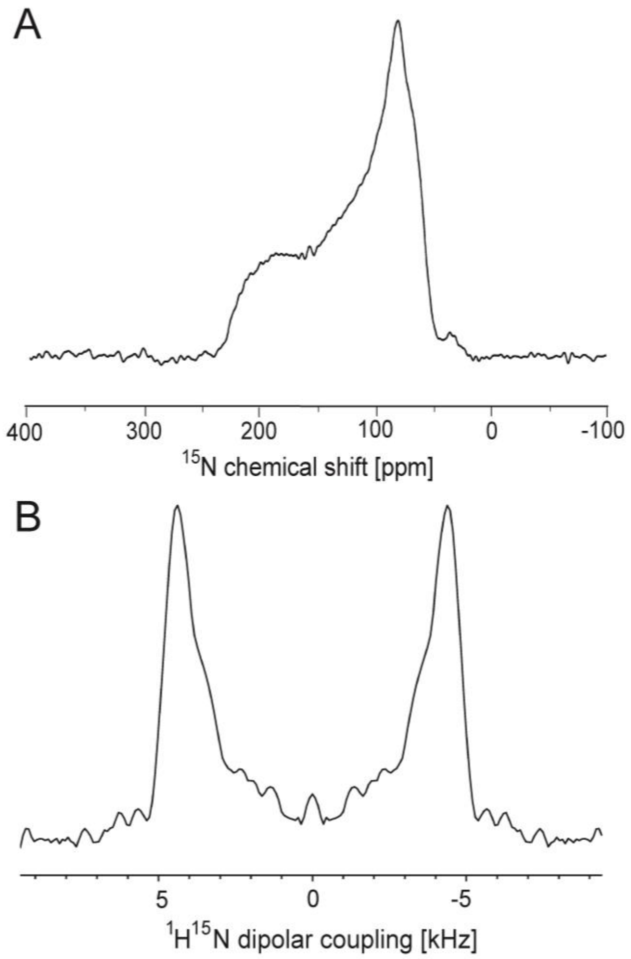

Figure S1: (*A*) A one-dimensional solid-state ^{15}N-NMR powder spectrum of ΔE5 was used to determine the principal values of the ^{15}N-CSA tensor. (*B*) A maximum ^{1}H-^{15}N dipolar coupling corresponding to a peak position (half splitting) of 8.8 kHz was determined from a SAMMY spectrum (only the projection of ^{1}H-^{15}N dipolar coupling dimension is shown) of the protein powder using the same experimental parameters as for the reconstituted protein.

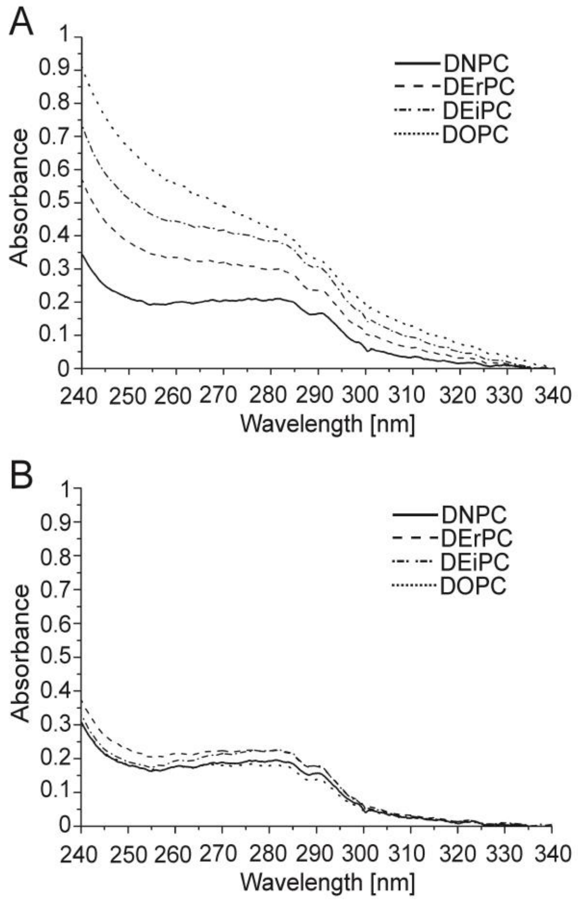

Figure S2: (*A*) UV spectra of SRCD samples of ΔE5 in different lipid vesicles showing increasing scattering contributions for decreasing bilayer thickness. (*B*) After dilution (1:20) in 10 mM SDS and ultrasound waterbath treatment (for 10 min) the aggregates are dissolved by the detergent, and now the absorption spectra match each other due to the absence of scattering contributions.

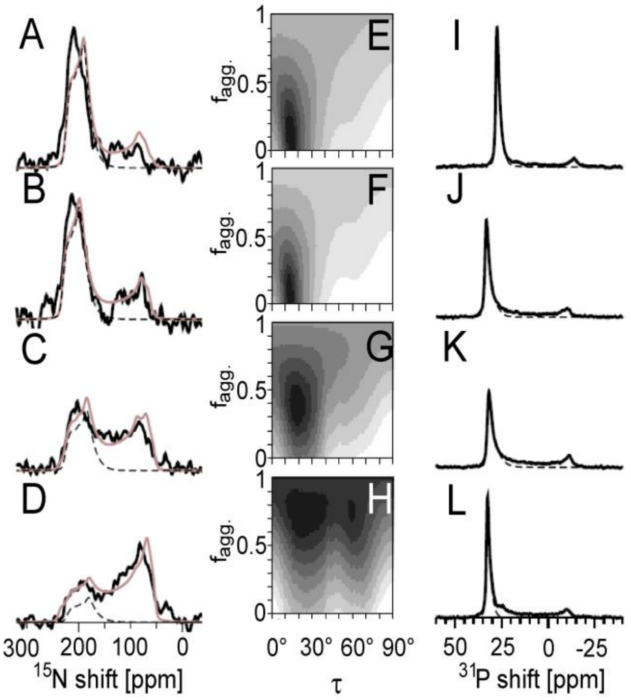

Figure S3: One-dimensional solid-state ^{15}N- and ^{31}P-NMR analysis was used to assess the membrane alignment and aggregation tendency of ΔE5 (here protein batch 2). The uniformly ^{15}N-labeled protein was reconstituted in bilayers of different thickness, namely in DNPC (*A*/*E*/*I*), DErPC (*B*/*F*/*J*), DEiPC (*C*/*G*/*K*) and DOPC (*D*/*H*/*L*). To deconvolute the experimental ^{15}N-NMR spectra (*A-D*, *solid black lines*), they were fitted with simulated lineshapes representing three fractions: well-oriented peptide, properly reconstituted peptide in mis-aligned membrane regions, and aggregated peptide. This way, the helix tilt angle of the well-oriented peptide population (*dashed lines*) could be estimated, and the sum of all three contributions is also shown (*solid grey lines*). The agreement between the calculated and experimental spectra as a function of tilt angle and aggregated fraction was judged from RMSD plots (*E-H*, black indicating the lowest RMSD value). In parallel, solid-state ^{31}P-NMR of the phospholipids (*I-L*) was used to assess the quality of alignment of the lipid matrix. For each samples the proportion of well-oriented lipid (*dashed grey line*) compared to mis-aligned membranes was obtained and used to fit the corresponding ^{15}N-spectra. In this iterative analysis, an increasing tilt angle of the E5 helix (away from the membrane normal) and an increasing amount of aggregated peptide was found with decreasing bilayer thickness upon going from DNPC to DOPC, see also Tables 2 and S1.

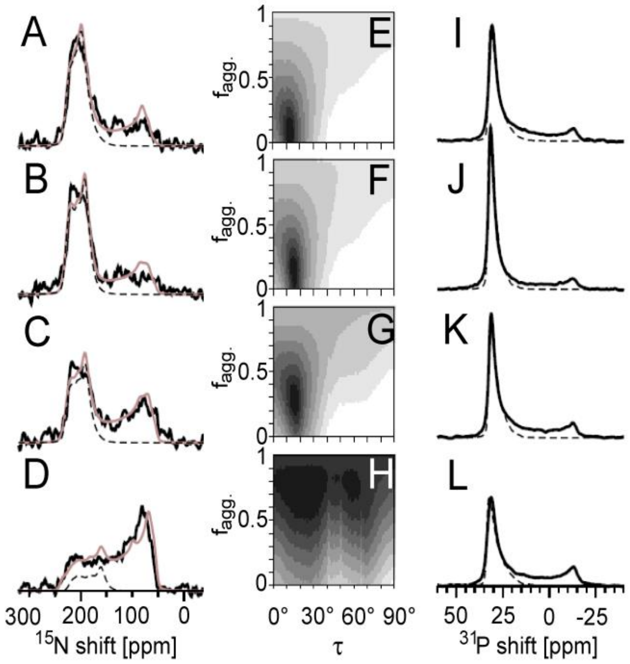

Figure S4: One-dimensional solid-state ^{15}N- and ^{31}P-NMR analysis was used to assess the membrane alignment and aggregation tendency of ΔE5 (here protein batch 3). See Figs. 2 and S3 for details.

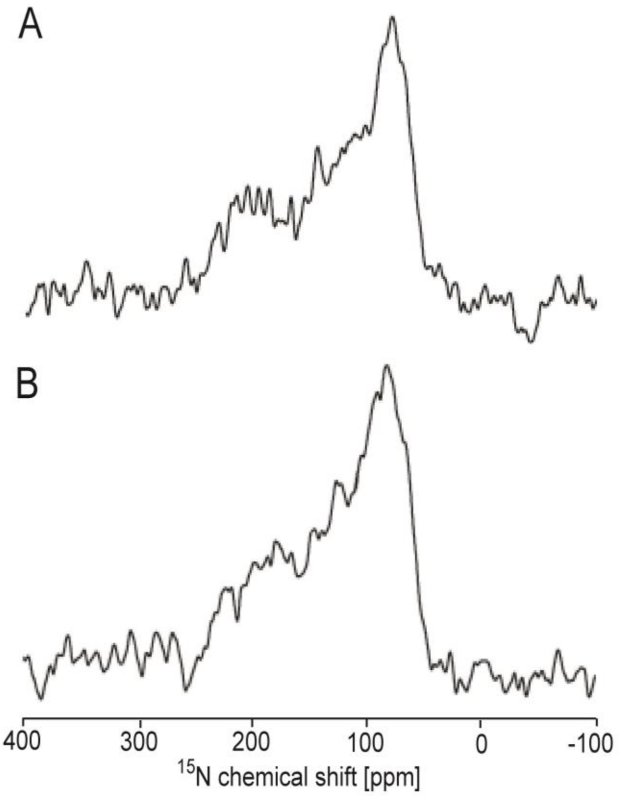

Figure S5: One-dimensional solid-state ^{15}N-NMR spectra of ΔE5 in macroscopically oriented samples of (*A*) DMPC and (*B*) POPC, acquired at 30°C and 20°C, respectively. Essentially pure powder spectra are observed due to protein aggregation in these conventional lipid bilayers.

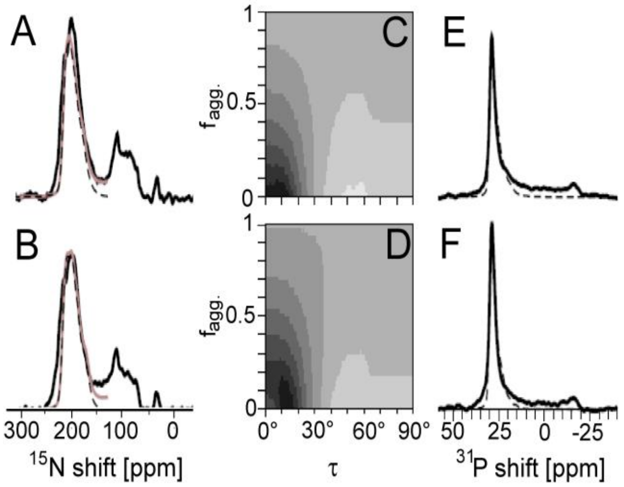

Figure S6: One-dimensional solid-state ^{15}N- and ^{31}P-NMR analysis was used to assess the membrane alignment and aggregation tendency of ^{15}N-PDGFR-TMD in presence of the ^{14}N-ΔE5 in DErPC. Two different samples were measured and analyzed. To deconvolute the experimental ^{15}N-NMR spectra (*A*/*B*), the same procedure as used for the pure PDGFR-TMD was applied. The ^{15}N-NMR spectrum shown in (*A*) corresponds to the spectrum shown in Fig. 5 *A*. The agreement between the calculated and experimental spectra as a function of tilt angle and aggregated fraction was judged from RMSD plots (*C*/*D*, black indicating the lowest RMSD value). In parallel, solid-state ^{31}P-NMR of the phospholipids (*E*/*F*) was used to assess the quality of alignment of the lipid matrix. No change of the lineshape was found compared to the spectrum of the pure protein in these bilayers (compare to Fig. 3 *B*), indicating that the tilt angle of the PDGFR-TMD helix has not changed due to the presence of the ΔE5. See Figs. 2 and S3 for details, and also Tables 3 and S2.

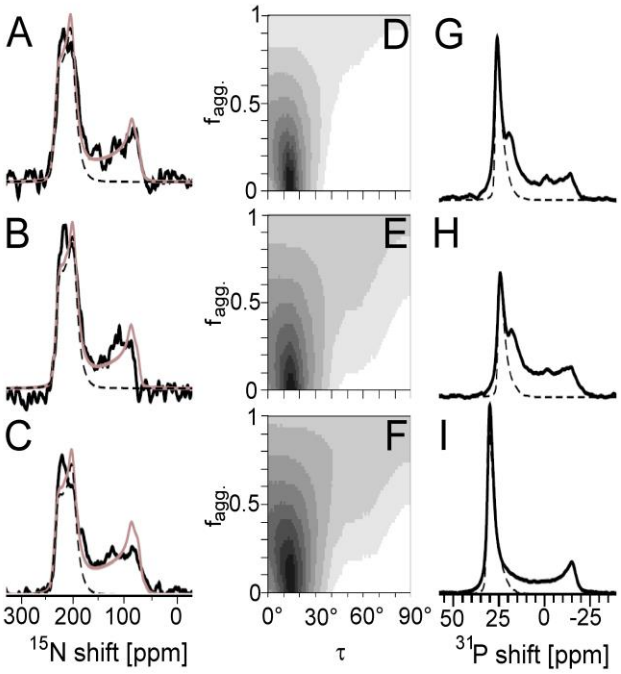

Figure S7: One-dimensional solid-state ^{15}N- and ^{31}P-NMR analysis was used to assess the membrane alignment and aggregation tendency of ^{15}N-ΔE5 in presence of the ^{14}N-PDGFR-TMD in DErPC. Three different samples were measured and analyzed. To deconvolute the experimental ^{15}N-NMR spectra (*A*-*C*), the same procedure as used for the pure ΔE5 protein was applied. The ^{15}N-NMR spectrum shown in (*A*) corresponds to the spectrum shown in Fig. 5 *B*. The agreement between the calculated and experimental spectra as a function of tilt angle and aggregated fraction was judged from RMSD plots (*D*-*F*, black indicating the lowest RMSD value). In parallel, solid-state ^{31}P-NMR of the phospholipids (*G*-*I*) was used to assess the quality of alignment of the lipid matrix. No change of the lineshape was found compared to the spectrum of the pure protein in these bilayers (compare to Fig. 2 *B*), indicating that the tilt angle of the E5 helix has not changed due to the presence of the PDGFR-TMD. See Figs. 2 and S3 for details, and also Tables 3 and S2.

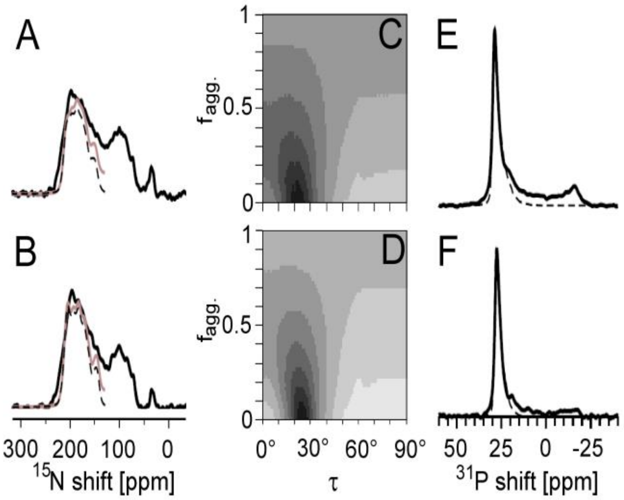

Figure S8: One-dimensional solid-state ^{15}N- and ^{31}P-NMR analysis was used to assess the membrane alignment and aggregation tendency of ^{15}N-PDGFR-TMD in presence of the ^{14}N-ΔE5 in DOPC. Two different samples were measured and analyzed. To deconvolute the experimental ^{15}N-NMR spectra (*A*/*B*), the same procedure as used for the pure PDGFR-TMD was applied. The ^{15}N-NMR spectrum shown in (*B*) corresponds to the spectrum shown in Fig. 5 *C*. The agreement between the calculated and experimental spectra as a function of tilt angle and aggregated fraction was judged from RMSD plots (*C*/*D*, black indicating the lowest RMSD value). In parallel, solid-state ^{31}P-NMR of the phospholipids (*E*/*F*) was used to assess the quality of alignment of the lipid matrix. No change of the lineshape was found compared to the spectrum of the pure protein in these bilayers (compare to Fig. 3 *D*), indicating that the tilt angle of the PDGFR-TMD helix has not changed due to the presence of the ΔE5. See Figs. 2 and S3 for details, and also Tables 3 and S2.

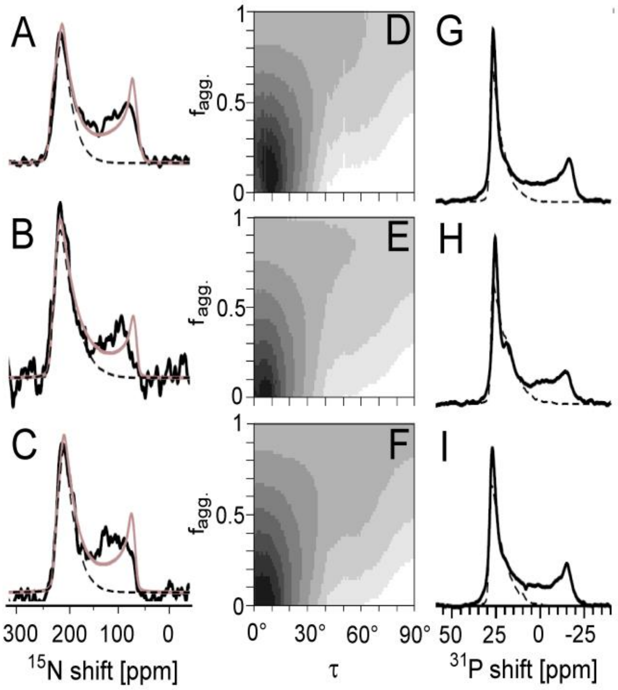

Figure S9: One-dimensional solid-state ^{15}N- and ^{31}P-NMR analysis was used to assess the membrane alignment and aggregation tendency of ^{15}N-ΔE5 in presence of the ^{14}N-PDGFR-TMD in DOPC. Three different samples were measured and analyzed. To deconvolute the experimental ^{15}N-NMR spectra (*A*-*C*), the same procedure as used for the pure ΔE5 protein was applied. The ^{15}N-NMR spectrum shown in (*A*) corresponds to the spectrum shown in Fig. 5 *D*. The agreement between the calculated and experimental spectra as a function of tilt angle and aggregated fraction was judged from RMSD plots (*D*-*F*, black indicating the lowest RMSD value). In parallel, solid-state ^{31}P-NMR of the phospholipids (*G*-*I*) was used to assess the quality of alignment of the lipid matrix. A large change of the lineshape was found compared to the spectrum of the pure protein in these bilayers (compare to Fig. 2 *D*), indicating a well-reconstituted and transmembrane aligned E5 helix caused by the presence of the PDGFR-TMD. See Figs. 2 and S3 for details, and also Tables 3 and S2.

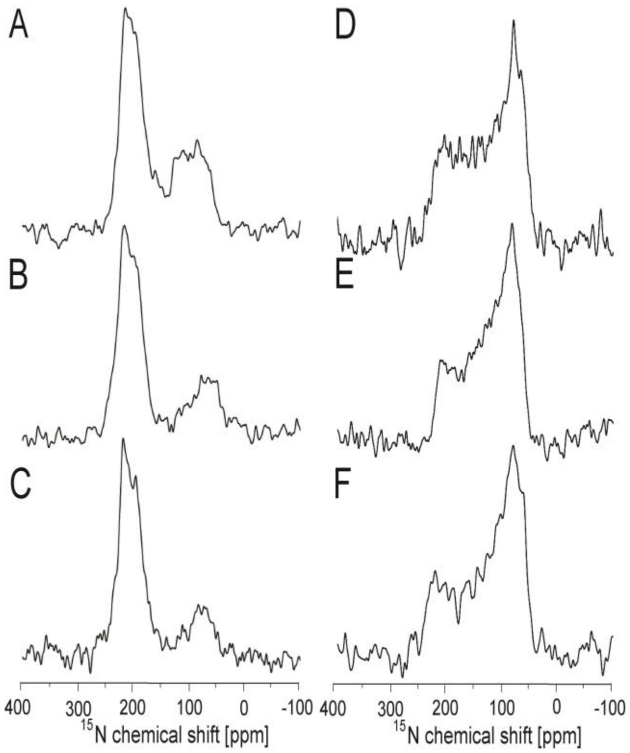

Figure S10: One-dimensional solid-state ^{15}N-NMR spectra of ΔE5 in macroscopically oriented lipid bilayers of DErPC (*A*-*C*) and DOPC (*D*-*F*) at different protein-to-lipid ratios of (*A*/*D*) 1:200, (*B*/*E*) 1:100 and (*C*/*F*) 1:50 (mol/mol). In DErPC, no changes of the transmembrane orientation were observed, indicating a protein and lipid concentration independent alignment of ΔE5. In DOPC, a powder lineshape was found at all protein-to-lipid ratios indicating that protein aggregation cannot be prevent by an increase of the lipid concentration.

SUPPORTING TABLES

Table S1: Detailed results of 1D solid-state NMR analysis of ΔE5 and PDGFR-TMD in lipid bilayers with different thickness.

ΔE5 protein batch 1 (Fig. 2)

Lipid	τ	S_{mol}	$f_{agg.}$	RMSD
**DNPC**	8°	1	22%	0.00098
**DErPC**	12°	1	10%	0.00127
**DEiPC**	16°	1	38%	0.00095
**DOPC**	16°	1	88%	0.00062

ΔE5 protein batch 2 (Fig. S3)

Lipid	τ	S_{mol}	$f_{agg.}$	RMSD
**DNPC**	15°	0.90	10%	0.00222
**DErPC**	13°	0.96	6%	0.00176
**DEiPC**	18°	0.92	38%	0.00152
**DOPC**	19°	0.90	72%	0.00158

ΔE5 protein batch 3 (Fig. S4)

Lipid	τ	S_{mol}	$f_{agg.}$	RMSD
**DNPC**	13°	0.94	6%	0.00122
**DErPC**	16°	0.94	14%	0.00132
**DEiPC**	16°	0.94	28%	0.00110
**DOPC**	28°	0.98	72%	0.00147

PDGFR-TMD (Fig. 3)

Lipid	τ	S_{mol}	$f_{agg.}$	RMSD
**DNPC**	3°	1	2%*	0.00398
**DErPC**	10°	1	0%*	0.00278
**DEiPC**	14°	1	0%*	0.00150
**DOPC**	22°	1	0%*	0.00166

1D solid-state ^{15}N-NMR analysis of the ΔE5 protein and PDGFR-TMD concerning their membrane orientation (helix tilt angle τ with respect to the bilayer normal), their molecular order parameter S_{mol} and their aggregation tendency (percentage $f_{agg.}$) in different lipid membranes. The uniformly ^{15}N-labeled ΔE5 and PDGFR-TMD were reconstituted in lipid bilayers of DNPC, DErPC, DEiPC and DOPC. For ΔE5 three individual samples (derived from three different protein batches) per lipid were analysed as described in the text. *For PDGFR-TMD the fraction of aggregated protein is less reliable, as the up-field part of the 1D-NMR spectrum (<130 ppm) was not included in the simulations (see text and Fig. 3). The agreement of the calculated and experimental spectra as a function of tilt angle and aggregated fraction was judged by the root of mean-square deviation (RMSD).

Table S2: Detailed results of 1D solid-state NMR analysis of the ΔE5/PDGFR-TMD mixtures in lipid bilayers with different thickness.

Mixture	Lipid	τ	S_{mol}	$f_{agg.}$	RMSD
**^{15}N-PDGFR-TMD/^{14}N-ΔE5 (1:1)**	**DErPC**	13°	1	0%*	0.00051
		10°	1	0%*	0.00258
	**DOPC**	24°	1	0%*	0.00037
		22°	1	0%*	0.00221
**^{15}N-ΔE5/^{14}N-PDGFR-TMD (1:1)**	**DErPC**	14°	0.9	0%	0.00030
		13°	0.92	2%	0.00108
		14°	0.92	8%	0.00025
	**DOPC**	9°	1	10%	0.00115
		6°	0.98	0	0.00031
		7°	0.92	0	0.00030

1D solid-state ^{15}N-NMR analysis of the ΔE5 protein and PDGFR-TMD concerning their membrane orientation (helix tilt angle τ with respect to the bilayer normal), their molecular order parameter S_{mol} and their aggregation tendency ($f_{agg.}$) in different lipid membranes, each protein in presence of the respective other one. For ΔE5 three individual samples per lipid and for the PDGFR-TMD two samples were analysed. *For PDGFR-TMD the fraction of aggregated protein is less reliable, as the up-field part of the 1D-NMR spectrum (<130 ppm) was not included in the simulations (see text and Fig. 3). The agreement of the calculated and experimental spectra as a function of tilt angle and aggregated fraction was judged by the root of mean-square deviation (RMSD).

SUPPORTING REFERENCES

1. Clarke, D. T., and G. Jones. 2004. CD12: a new high-flux beamline for ultraviolet and vacuum-ultraviolet circular dichroism on the SRS, Daresbury. *J Synchrotron Radiat,* 11:142–149.
2. Bürck, J., S. Roth, D. Windisch, P. Wadhwani, D. Moss, and A. S. Ulrich. 2015. UV-CD12: synchrotron radiation circular dichroism beamline at ANKA. *J Synchrotron Radiat,* 22:844–852.
3. Wien, F., and B. A. Wallace. 2005. Calcium fluoride micro cells for synchrotron radiation circular dichroism spectroscopy. *Appl Spectrosc,* 59:1109–1113.
4. Sreerama, N., and R. W. Woody. 2000. Estimation of protein secondary structure from circular dichroism spectra: comparison of CONTIN, SELCON, and CDSSTR methods with an expanded reference set. *Anal. Biochem.,* 287:252–260.
5. van Stokkum, I. H., H. J. Spoelder, M. Bloemendal, R. van Grondelle, and F. C. Groen. 1990. Estimation of protein secondary structure and error analysis from circular dichroism spectra. *Anal. Biochem.,* 191:110–118.
6. Whitmore, L., and B. A. Wallace. 2004. DICHROWEB, an online server for protein secondary structure analyses from circular dichroism spectroscopic data. *Nucleic Acids Res.,* 32:W668-73.
7. Lobley, A., L. Whitmore, and B. A. Wallace. 2002. DICHROWEB: an interactive website for the analysis of protein secondary structure from circular dichroism spectra. *Bioinformatics,* 18:211–212.
8. Wallace, B. A., and R. W. Janes. 2009. Modern techniques for circular dichroism and synchrotron radiation circular dichroism spectroscopy. *In* Volume 1 Advances in Biomedical Spectroscopy, B. A. Wallace and R. W. Jones, editors, IOS Press, Amsterdam.
9. Pace, C. N., F. Vajdos, L. Fee, G. Grimsley, and T. Gray. 1995. How to measure and predict the molar absorption coefficient of a protein. *Protein Sci.,* 4:2411–2423.
10. Gor'kov, P. L., E. Y. Chekmenev, C. Li, M. Cotten, J. J. Buffy, N. J. Traaseth, G. Veglia, and W. W. Brey. 2007. Using low-E resonators to reduce RF heating in biological samples for static solid-state NMR up to 900 MHz. *J. Magn. Reson.,* 185:77–93.
11. M.H. Levitt, D. S. a. R. E. 1986. Spin dynamics and thermodynamics in solid-state NMR cross polarization. *J. Chem. Phys.,* 1986:4243–4255.
12. Nevzorov, A. A., and S. J. Opella. 2003. A "magic sandwich" pulse sequence with reduced offset dependence for high-resolution separated local field spectroscopy. *J. Magn. Reson.,* 164:182–186.
13. Nevzorov, A. A., and S. J. Opella. 2007. Selective averaging for high-resolution solid-state NMR spectroscopy of aligned samples. *J. Magn. Reson.,* 185:59–70.
