# Supplementary material for: Hydrophobic Mismatch Drives the Interaction of E5 with the Transmembrane Segment of PDGF Receptor
Source: Biophys J. 2015 Aug 18;109(4):737–49. doi: 10.1016/j.bpj.2015.07.022 (PMC4547410; doi:10.1016/j.bpj.2015.07.022)
Supplement: Document S2. Article plus Supporting Material [file mmc2.pdf]

Biophysical Journal Volume 109 August 2015 737–749

Article

Hydrophobic Mismatch Drives the Interaction of E5 with the Transmembrane Segment of PDGF Receptor

Dirk Windisch,[1] Colin Ziegler,[2] Stephan L. Grage,[1] Jochen Bürck,[1] Marcel Zeitler,[2] Peter L. Gor'kov,[3] and Anne S. Ulrich[1,2,*]
[1]Institute of Biological Interfaces (IBG-2) and [2]Institute of Organic Chemistry, Karlsruhe Institute of Technology, Karlsruhe, Germany; and [3]National High Magnetic Field Laboratory, Tallahassee, Florida

ABSTRACT The oncogenic E5 protein from bovine papillomavirus is a short (44 amino acids long) integral membrane protein that forms homodimers. It activates platelet-derived growth factor receptor (PDGFR) β in a ligand-independent manner by transmembrane helix-helix interactions. The nature of this recognition event remains elusive, as numerous mutations are tolerated in the E5 transmembrane segment, with the exception of one hydrogen-bonding residue. Here, we examined the conformation, stability, and alignment of the E5 protein in fluid lipid membranes of substantially varying bilayer thickness, in both the absence and presence of the PDGFR transmembrane segment. Quantitative synchrotron radiation circular dichroism analysis revealed a very long transmembrane helix for E5 of ~26 amino acids. Oriented circular dichroism and solid-state ^{15}N-NMR showed that the alignment and stability of this unusually long segment depend critically on the membrane thickness. When reconstituted alone in exceptionally thick DNPC lipid bilayers, the E5 helix was found to be inserted almost upright. In moderately thick bilayers (DErPC and DEiPC), it started to tilt and became slightly deformed, and finally it became aggregated in conventional DOPC, POPC, and DMPC membranes due to hydrophobic mismatch. On the other hand, when E5 was co-reconstituted with the transmembrane segment of PDGFR, it was able to tolerate even the most pronounced mismatch and was stabilized by binding to the receptor, which has the same hydrophobic length. As E5 is known to activate PDGFR within the thin membranes of the Golgi compartment, we suggest that the intrinsic hydrophobic mismatch of these two interaction partners drives them together. They seem to recognize each other by forming a closely packed bundle of mutually aligned transmembrane helices, which is further stabilized by a specific pair of hydrogen-bonding residues.

INTRODUCTION

Hydrophobic mismatch between lipid membranes and integral proteins can be an important regulator of protein function (1–3). A single-span (bitopic) transmembrane protein with a given hydrophobic length is expected to respond to the local membrane thickness. It has to avoid unfavorable exposure of hydrophobic regions to the hydrophilic environment by minimizing the energy of the hydrophobic mismatch. When a transmembrane domain (TMD) is very long, this can result in tilting of the helix, changes in the backbone conformation, or oligomerization (1,4). In the case of oligomers or compact polytopic membrane proteins, the entire bundle is obviously less sensitive to bilayer thickness.

Here, we investigated the mechanism of signal transduction by platelet-derived growth factor receptor (PDGFR) β, a single-span transmembrane protein. PDGFR belongs to the family of receptor tyrosine kinases (RTKs) that are involved in development, central nervous system formation, and angiogenesis (5). It is activated when a growth factor binds simultaneously to two receptor monomers. Dimerization leads to a rearrangement of the TMDs that brings the cytosolic kinase domains into close contact, enabling them to undergo transautophosphorylation and trigger signal transduction (6,7). The dimer contact encompasses the extramembranous domains, but the TMDs are also actively involved in this allosteric activation process (8). Many RTKs, including PDGFR, are known to be assembled as preformed dimers (8–11) in which the transmembrane segments can rotate from an inactive to an activated state upon ligand binding. Our recent structural investigations of the PDGFR-TMD suggested that the helix tilt angle and the stability of the preformed dimer are controlled by hydrophobic matching to the lipid bilayer thickness (12).

PDGFR can also be activated in a ligand-independent manner by the oncogenic E5 protein from bovine papillomavirus (13–20). With only 44 amino acids, this is one of the shortest known integral membrane proteins. E5 activates the receptor through highly specific interactions between the transmembrane segments, the nature of which is not yet fully understood (21–23). Complex formation is known to slow down receptor internalization, and the sustained signaling can lead to cancer. To be able to interact with PDGFR, E5 itself needs to be present as a dimer, which is

Submitted January 14, 2015, and accepted for publication July 14, 2015.
*Correspondence: anne.ulrich@kit.edu
This is an open access article under the CC BY license (http://creativecommons.org/licenses/by/4.0/).
Editor: Francesca Marassi.
© 2015 The Authors
0006-3495/15/08/0737/13
CrossMark
http://dx.doi.org/10.1016/j.bpj.2015.07.022

maintained by two disulfide bridges within a short extramembranous stretch at the C-terminus. Interestingly, E5 mutants lacking these cysteines or even the entire C-terminal region are still able to form dimers (24–28), suggesting that dimerization is also driven by specific interhelical contacts between the TMDs. In infected cells, the E5 protein is found in the plasma membrane (29) but is located predominantly in the Golgi compartment, where it is able to activate PDGFR (30).

Structural investigations using infrared and circular dichroism (CD) spectroscopy have shown that E5 adopts a predominantly α-helical conformation when reconstituted in DMPC lipid bilayers and detergent micelles (22,24,25,31), but to date no high-resolution structure is available for E5 alone or in a complex with PDGFR. In general, it is very challenging to handle E5 because this highly hydrophobic protein has an intrinsic high tendency to aggregate, and the native sequence forms higher-mass oligomers due to nonspecific disulfide cross-linking. We recently demonstrated that we could resolve the latter problem by removing the cysteines by C-terminal truncation (28). The resulting truncated ΔE5 variant (containing the first 34 amino acids of the native sequence) is much more manageable and at the same time retains the same secondary structure, the same orientation in the membrane, and the same ability to self-associate as the wild-type protein. Thus, ΔE5 may be regarded as a representative model for the E5 TMD, and we used it here to conduct a detailed structure analysis in lipid bilayers.

Solid-state NMR and CD spectroscopy are ideally suited for investigating the structures, protein-lipid interactions, and protein-protein recognition of E5 and PDGFR. These methods can be routinely used to characterize the alignment of transmembrane helices in macroscopically oriented membrane samples. One-dimensional (1D) ^{15}N-NMR spectra provide a good estimate of the helix tilt angle and will readily reveal nonspecific protein aggregation (12). Two-dimensional (2D) separated-local-field ^{15}N-NMR experiments (32–37) display distinctive signal patterns, called polarity index slant angle (PISA) wheels, that can provide a direct measure of both the helix tilt angle and the azimuthal rotation angle (33,35,38). Such PISA wheels can be fitted to extract the structures of membrane proteins with high accuracy, as was done for the Vpu protein from HIV (39), the M2 protein channel of the influenza virus (40), and the M13 coat protein of bacteriophages (35).

Here, we examined the membrane alignment and stability of the E5 protein in lipid bilayers of varying thickness using a combination of ^{15}N-NMR PISA wheel analysis, synchrotron radiation CD (SRCD), and oriented CD (OCD). We thoroughly characterized the behavior of E5 as a function of hydrophobic mismatch, both on its own and in the presence of PDGFR, which has the same hydrophobic length.

MATERIALS AND METHODS

Cloning and expression of ΔE5 and PDGFR-TMD

The truncated mutant ΔE5 and the PDGFR-TMD were cloned and expressed as Trp-ΔLE-fusion proteins as previously described (12,28,31). After cleavage to remove the tags, all constructs retained an extra Gly at the N-terminus of the native sequence. Protein expression was carried out in *E. coli* BL21 (DE3) (Novagen, Darmstadt, Germany) at 37°C in LB medium for unlabeled proteins or in M9 minimal medium supplemented with 1 g L^{-1} of $(^{15}NH_4)_2SO_4$ for uniformly ^{15}N-labeled proteins.

SRCD and OCD sample preparation and measurements

SRCD and OCD sample preparation, measurements, and spectrum deconvolution were performed as described previously (28,31,41) and are described in detail in the Supporting Materials and Methods. Briefly, ΔE5 was reconstituted in small unilamellar vesicles (SUVs) made of long-chain DNPC (1,2-dinervonoyl-*sn*-glycero-3-phosphocholine; di-C24:1), DErPC (1,2-dierucoyl-*sn*-glycero-3-phosphocholine; di-C22:1), and DEiPC (1,2-dieicosenoyl-*sn*-glycero-3-phosphocholine; di-C20:1), with a protein/lipid ratio of 1:50 (mol/mol), in addition to the more common lipids DOPC (1,2-dioleoyl-*sn*-glycero-3-phosphocholine; di-C18:1), POPC (1-palmitoyl-2-oleoyl-*sn*-glycero-3-phosphocholine; C16:0/C18:1), and DMPC (1,2-dimyristoyl-*sn*-glycero-3-phosphocholine; di-C14:0). All lipids were purchased from Avanti Polar Lipids (Alabaster, AL). SRCD spectra were collected on the UV-CD12 beamline (formerly called the CD12 beamline at Daresbury Laboratory, Warrington, UK) at the ANKA storage ring (Karlsruhe Institute of Technology, Karlsruhe, Germany). The beamline components and its experimental end-station have previously been described in detail (42,43). SRCD spectra were recorded at 20°C for DOPC and DEiPC, at 30°C for DErPC, and at 35°C for DNPC to stay above the lipid phase transition temperature, using a cell holder that was thermostatted by Peltier elements. Secondary structure analysis was performed using the DichroWeb server (44–47). The SRCD spectra shown in Fig. 1 A have been deposited in the Protein Circular Dichroism Data Bank (PCDDB) (48) (http://pcddb.cryst.bbk.ac.uk/home.php) and are publically available under PCDDBID codes CD0004264000, CD0004265000, CD0004266000, and CD0004267000.

For OCD measurements, macroscopically aligned CD samples were prepared from the E5 vesicle suspensions by depositing an aliquot of the lipid suspension onto a quartz glass plate. OCD measurements were performed on a desktop Jasco instrument (Groß-Umstadt, Germany). To reduce possible spectral artifacts caused by a variable quality of protein reconstitution, at least three independent samples were prepared, measured, and finally averaged to get the final OCD spectrum.

Solid-state NMR sample preparation and measurements

Solid-state NMR sample preparation and measurements were performed as described previously (12,28) and are described in detail in the Supporting Materials and Methods. Briefly, uniformly ^{15}N-labeled ΔE5 and PDGFR-TMD were reconstituted together or alone in macroscopically aligned lipid bilayers made of DNPC, DErPC, DEiPC, and DOPC (protein/lipid ratio of 1:50 (mol/mol)). POPC and DMPC were also used, but due to severe protein aggregation, only limited data are shown in the Supporting Material. ^{15}N-NMR measurements were carried out on a Bruker Avance 600 MHz spectrometer (Bruker-Biospin, Karlsruhe, Germany) using a custom-built Low-E ^{1}HX probe equipped with cross-coil resonators of rectangular cross section (Karlsruhe Institute of Technology Karlsruhe, Germany, and National High Magnetic Field Laboratory, Tallahassee, FL) (49). For the NMR measurements, the temperature was set to 20°C for DOPC and

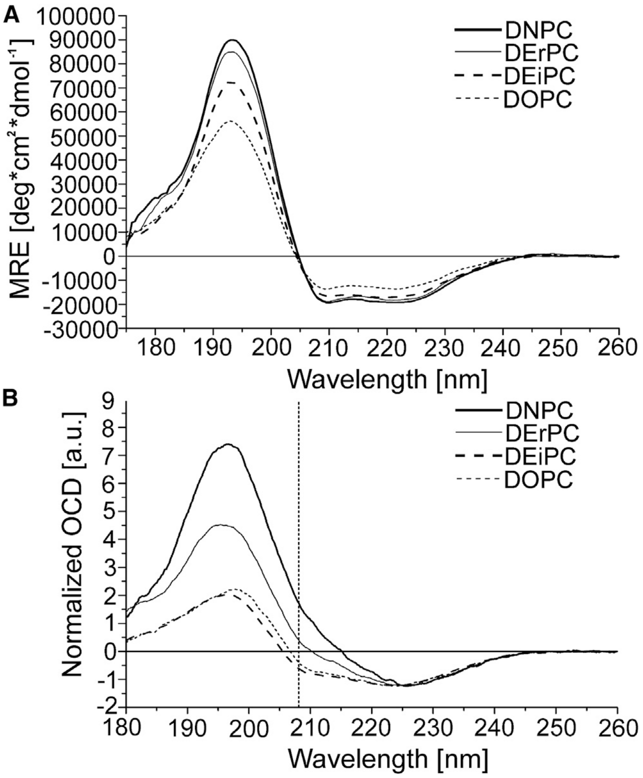

FIGURE 1 (*A*) SRCD spectra of ΔE5 reconstituted in different lipid vesicles (in isotropic suspension), showing a predominantly α-helical secondary structure irrespective of the bilayer thickness. The content of helical aggregates, however, is found to increase with decreasing bilayer thickness, as indicated by the reduction in signal intensities at shorter wavelengths due to absorption flattening and light scattering. (*B*) OCD spectra of ΔE5 reconstituted in macroscopically aligned lipid bilayers of different thickness. Spectra are normalized to the same intensity at 225 nm to illustrate the differences in the characteristic orientation-sensitive band at 208 nm (*dashed line*). ΔE5 has an upright orientation in thick DNPC membranes, as reflected by the positive ellipticity at 208 nm, whereas with decreasing bilayer thickness the ellipticity gradually becomes negative, indicating a more tilted alignment of the helix. A reduction in the overall signal intensity of the positive band is observed from DNPC to DOPC as a result of increased protein aggregation in thinner bilayers.

DEiPC, 30°C for DErPC, and 35°C for DNPC samples. The quality of the lipid alignment was checked by measuring ^{31}P-NMR spectra using a Hahn echo sequence. For ΔE5, three individual samples for each lipid (derived from three different protein batches) were analyzed.

Solid-state NMR evaluation of the helix tilt

To determine the helix tilt angle (defined between the membrane normal and the helix axis) and the molecular order parameter S_{mol} (a qualitative measure of mobility with $0 \leq |S_{mol}| \leq 1.0$), the lineshapes of the 1D NMR spectra of ΔE5 and PDGFR-TMD were deconvoluted into different fractions as previously described (12). Briefly, each sample was assumed to contain three different protein contributions corresponding to 1) protein in well-oriented bilayers, 2) protein in misaligned bilayers, and 3) protein that was not properly reconstituted and is therefore referred to as aggregated. The ratio of oriented to nonoriented bilayers, as well as the mosaic spread describing the quality of alignment of the oriented bilayer fraction, was obtained independently from the ^{31}P-NMR spectrum of each sample. We fitted the experimental ^{15}N-NMR spectra with simulated lineshapes that we calculated using these parameters from the ^{31}P-NMR data. We performed the simulations by systematically varying the helix tilt angle, the value of S_{mol}, and the fraction of aggregated protein (f_{agg}). The best fit between the experimental spectra and the calculated lineshapes was judged on the basis of the minimal root mean-square deviation (RMSD) over the intensities. The principal values of the ^{15}N-CSA tensor were determined from 1D measurements of the ΔE5 protein powder (Fig. S1 *A*) and set to 53.6 ppm, 77.1 ppm, and 218.5 ppm. The truncated ΔE5 peptide was modeled as an ideal α-helix for 28 core residues (Trp-5 to Trp-32) plus six randomly structured terminal residues. The detailed results of each analysis are shown in Table S1 and the averaged results are summarized in Table 2. To determine the helix tilt angle of ΔE5 from 2D NMR SAMMY measurements, we fitted the observed spectra with simulated PISA wheels of ideal α-helices by varying the tilt angle and S_{mol} value as previously described (50). An α-helical conformation with uniform dihedral angles $\Phi = -61°$ and $\Psi = -45°$ was used for all residues, and the angle between the ^{15}N chemical-shift tensor (principal axis corresponding to 218.5 ppm) and the NH vector was 18.5°. A maximum ^{1}H-^{15}N dipolar coupling corresponding to the peak-to-peak position (half splitting) of 8.8 kHz was determined from a SAMMY spectrum of the ΔE5 protein powder, using the same experimental parameters employed for the reconstituted protein (Fig. S1 *B*).

RESULTS

SRCD: conformation of ΔE5 in lipid membranes and influence of bilayer thickness

The common phospholipids DMPC and POPC are widely used as model membranes and are among the most favored lipids for general biophysical analyses. However, all of our initial attempts to incorporate the E5 protein into these standard lipid bilayers failed badly, as they led to severe protein aggregation. In an earlier study, we were able to reconstitute the wild-type and different analogs of E5 into DMPC, but only with the help of lyso-lipid that was added to soften the membrane and make it more tolerant to defects (31). Fortunately, we eventually succeeded in reconstituting E5 in lipid membranes made of much thicker DErPC in the liquid crystalline state (28). It is now clear that the fundamental problem with conventional lipids can be (retrospectively) attributed to a pronounced hydrophobic mismatch between the helix length and the bilayer thickness. Here, we demonstrate that one can routinely reconstitute E5 by using phospholipids with very long acyl chains, resulting in stable samples with well-aligned helices and with negligible aggregation.

To examine the influence of membrane thickness on the secondary structure and aggregation behavior of E5, we selected a series of phospholipids with long unsaturated acyl chains, whose phase transition temperatures are amenable to the experimental conditions of CD and NMR (saturated lipids would have required extremely high temperatures): DNPC (di-C24:1), DErPC (di-C22:1), and DEiPC (di-C20:1). In addition, we used the more common DOPC (di-C18:1), POPC (C16:0/C18:1), and DMPC (di-C14:0). Due to the number of methylene units in the fatty acyl chains, the hydrophobic thickness of the corresponding

model membrane increases from 25.6 Å in comparatively thin DMPC bilayers to 26.8 Å in conventional DOPC, 30.6 Å in thicker DEiPC, 34.4 Å in DErPC, and 38.2 Å in very thick DNPC bilayers (calculated according to Marsh (51)).

Generally, when unsaturated lipids are used to prepare SUVs for a CD analysis of membrane-bound proteins, it is virtually impossible to collect CD spectra with a good signal/noise ratio at wavelengths below 200 nm. This is due to the high background absorption of the lipid double bonds as well as to the intrinsic light scattering of the vesicles. SRCD, on the other hand, is ideally suited to tackle this challenge, because the 1000-fold higher photon flux makes background absorption less critical, and hence the spectral quality is greatly enhanced compared with conventional CD. We thus managed to collect high-quality SRCD spectra of the truncated ΔE5 analog reconstituted in SUVs made of several different long-chain lipids. As expected, ΔE5 showed a predominantly helical secondary structure in the tested environments (Fig. 1 *A*). Interestingly, the intensity of the CD bands, and especially that of the 194 nm band, decreased from very thick DNPC to the thinner DOPC bilayers. This apparent change in helix content does not represent a real change in the secondary structure of E5. Instead, it can be explained by an increasing contribution of absorption flattening, and by enhanced light-scattering artifacts, due to the formation of helical protein oligomers and/or aggregates, as previously described for the E5 wild-type protein (28,31). Protein oligomerization and aggregation are known to lead to an inhomogeneous distribution of the chromophores in a sample, which results in less protein signal being detected (52,53).

Fig. 1 *A* shows that E5 is well reconstituted in very thick DNPC lipid bilayers, and hardly any aggregation is observed. On the other hand, with decreasing bilayer thickness, more and more protein is seen to aggregate, resulting in a loss of signal intensity as described above. Notably, despite the increasing occurrence of aggregation, the helical lineshapes of the CD spectra indicate that the protein does not unfold during aggregation, but remains mostly helical. Additional signs of protein aggregation were obtained from NMR measurements (see Figs. 2 and 4), and from the increased degree of light scattering in the corresponding UV spectra (Fig. S2). Therefore, the secondary-structure deconvolution of the SRCD data is reliable only for DNPC and DErPC, and not for DEiPC or DOPC. When we analyzed it using the CONTIN algorithm, we found that ΔE5 has a total helix content of ~91% in DNPC and ~89% in DErPC, plus minor fractions of β-strand, turn, and random coil (Table 1). Notably, ~73% (corresponding to 26 amino acids) was found to be in an ideal α-helical conformation (α_R content) in DNPC. Outside of the membrane, the E5 helix is less regular, as reflected by the α_D content (six amino acids in DNPC), and is further flanked by unstructured C- and N-termini (corresponding to only one or two amino acids at either end of ΔE5). In the thinner membranes of DEiPC and DOPC, the E5 transmembrane segment is too long to match the hydrophobic bilayer core, which results in an increased extent of protein aggregation and/or nonspecific oligomerization.

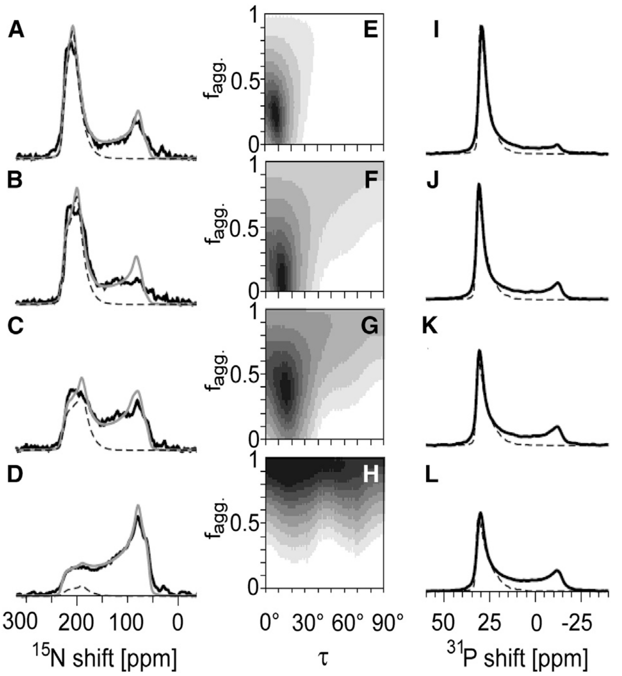

FIGURE 2 1D solid-state ^{15}N- and ^{31}P-NMR analyses were used to assess the membrane alignment and aggregation tendency of ΔE5 (protein batch 1 is shown here; see Figs. S3 and S4 for batches 2 and 3). (*A–L*) The uniformly ^{15}N-labeled protein was reconstituted in bilayers of different thickness, namely, DNPC (*A*, *E*, and *I*), DErPC (*B*, *F*, and *J*), DEiPC (*C*, *G*, and *K*), and DOPC (*D*, *H*, and *L*) (see Fig. S5 for POPC and DMPC). (*A–D*) To deconvolute the experimental ^{15}N-NMR spectra (*solid black lines*), they were fitted with simulated lineshapes representing three fractions: well-oriented peptide, properly reconstituted peptide in misaligned membrane regions, and aggregated peptide. In this way, the helix tilt angle of the well-oriented peptide population (*dashed lines*) could be estimated; the sum of all three contributions is also shown (*solid gray lines*). The agreement between the calculated and experimental spectra as a function of tilt angle and aggregated fraction was judged from RMSD plots (*E–H*; *black* indicates the lowest RMSD value). (*I–L*) In parallel, solid-state ^{31}P-NMR of the phospholipids (*solid black lines*) was used to assess the quality of alignment of the lipid matrix. For each sample, the proportion of well-oriented lipid (*dashed gray line*) compared with misaligned membranes was obtained and used to fit the corresponding ^{15}N spectra. In this iterative analysis, an increasing tilt angle of the E5 helix (away from the membrane normal) and an increasing amount of aggregated peptide were found with decreasing bilayer thickness upon going from DNPC to DOPC (see also Table 2).

OCD: influence of the bilayer thickness on the orientation of the E5 transmembrane helix

For PDGFR, it was recently shown that the membrane thickness affects not only the quality of the reconstituted protein sample but also the actual orientation of the transmembrane

TABLE 1 Secondary Structure of ΔE5 in Lipid Bilayers

Lipid	α_R (%)	α_D (%)	Sum α-Helix (%)	β-Strand (%)	β-Turn (%)	Unstructured (%)	NRMSD
DNPC	73 (26)	18 (6)	91 (32)	5 (2)	3 (1)	1 (0)	0.044
DErPC	70 (24)	19 (7)	89 (31)	5 (2)	3 (1)	4 (1)	0.043

Deconvolution results of ΔE5 SRCD spectra using the CONTIN algorithm. The secondary-structure contents are given in % and the corresponding numbers of amino acids are shown in brackets. $\alpha_{R,}$ regular helix; α_D, distorted helix; NRMSD, normalized RMSD.

helix in the lipid bilayer (12). Since E5 activates PDGFR via helix-helix interactions, it is of fundamental interest now to find out how the E5 helix responds to the bilayer thickness, and to compare this with PDGFR. To address the alignment of ΔE5, we reconstituted it in macroscopically oriented model membranes with varying bilayer thickness. OCD of such samples provides a fast and sensitive way to estimate the tilt angle of a straight α-helix in a lipid bilayer (12,28,31,41,54–64). A helix that is aligned parallel to the membrane surface gives rise to a dominant-negative 208 nm band that has a stronger negative intensity than the 222 nm band (65,66). For a tilted helix, on the other hand, the negative 208 nm fingerprint band loses its intensity, and it will reach even positive values for a fully upright transmembrane orientation. When ΔE5 was reconstituted in very thick DNPC, a positive ellipticity at 208 nm was observed, indicating an upright insertion of ΔE5 (Fig. 1 *B*). In moderately thick DErPC bilayers, the band was reduced but still positive, indicating that the protein was slightly tilted. Finally, in thinner DEiPC and DOPC membranes, the 208 nm band became negative, which is a sign that the protein had tilted even more in these membranes. At the same time, a strong reduction in the overall signal intensities was observed, especially at short wavelengths. These artifacts of absorption flattening and light scattering can be attributed to an increasing degree of protein aggregation in these bilayers, as was observed by conventional CD (see above). Clearly, with decreasing bilayer thickness, the E5 protein tries to adapt by tilting, but at the same time an increasing amount of the protein becomes aggregated due to hydrophobic mismatch.

1D-NMR: estimation of the membrane alignment and stability of E5

After completing the qualitative OCD assessment, we performed 1D solid-state NMR measurements of uniformly ^{15}N-labeled protein samples to determine the orientation of the protein helix more accurately and to quantify the fraction of aggregated material. For this purpose, we reconstituted uniformly ^{15}N-labeled ΔE5 in macroscopically aligned lipid bilayers made of the same lipids used in the above SRCD and OCD experiments. For each lipid, we analyzed three individual samples to assess the reproducibility of the effects. Generally, in the solid-state ^{15}N-NMR spectra of transmembrane helices, two regions can be distinguished along the chemical shift axis: signals in the downfield region at ~200 ppm originate from well-aligned transmembrane segments, and the upfield region contains the broader peak of a powder pattern at ~75 ppm. These upfield signals represent not only the aggregated protein but also any well-reconstituted E5 that happens to be located in a misaligned part of the (nonideal) oriented sample. Within the series of NMR spectra, we see that the peak in the downfield region at 200 ppm shifts to lower ppm values with decreasing membrane thickness and shows a slight increase in linewidth (Figs. 2, S3, and S4). This observation suggests that the protein becomes more tilted within the series from DNPC to DErPC to DEiPC, in full support of the OCD data described above. At the same time, the signal intensity is found to decrease considerably in the downfield region, but increases in the upfield region at ~75 ppm. In the last lipid of the series, DOPC, essentially a pure powder spectrum is observed, corresponding to complete protein aggregation, as was also the case in POPC and DMPC lipid bilayers (Fig. S5). A similar behavior is seen in two further series of NMR experiments with freshly prepared samples using different batches of protein (Figs. S3 and S4), confirming that the observed effects are reproducible. These data clearly show that the unusually long E5 helix is most stable in very thick DNPC, and it responds to hydrophobic mismatch by adjusting its tilt angle to some extent in moderately thick bilayers. Given the dramatic increase in protein aggregation when approaching standard DOPC, POPC, and DMPC membranes, E5 is obviously unable to compensate for strong mismatch and instead becomes aggregated.

Analogous changes are seen in the corresponding ^{31}P-NMR spectra of phospholipids in the same samples. These spectra tend to show two components: well-aligned bilayers giving rise to a narrow signal in the downfield region at ~30 ppm, and misaligned bilayers producing a powder spectrum with a peak in the upfield region at ~−15 ppm. To assess their relative contributions, we deconvoluted the two fractions of well-aligned and misaligned membrane domains by lineshape simulations of the ^{31}P-NMR spectra. We then used this information to analyze the lineshapes of the more complex ^{15}N-NMR spectra. These spectra consist of contributions from 1) well-oriented protein in well-aligned membranes, 2) well-oriented protein in misaligned membrane regions, and 3) aggregated protein that was not properly reconstituted, as explained in more detail in Muhle-Goll et al. (12).

We fitted the ^{15}N-NMR data using a variable helix tilt angle τ (defined as the angle of the helix axis with respect

to the membrane normal), a variable helix order parameter S_{mol}, and a variable degree of protein aggregation, in a manner similar to that described in our previous NMR study of PDGFR-TMD (12). For each different lipid sample, we could thus obtain the tilt angle of ΔE5 and quantify the f_{agg}. This deconvolution showed that the helix tilt angle increased slightly, from ~12° ± 3° in thick DNPC bilayers to 14° ± 2° in DErPC, to 17° ± 1° in DEiPC, and finally to 21° ± 5° in thin DOPC (Tables 2 and S1; nominal errors were obtained from the averages of the three batches of protein). At the same time, the amount of aggregated protein increased dramatically, from roughly 13% ± 7% in DNPC and 10% ± 3% in DErPC to 35% ± 5% in DEiPC and up to severe aggregation (77% ± 8%) in DOPC. The order parameter S_{mol} was found to be close to 1.0 in all lipid systems, indicating that the helix did not undergo any wobbling motion.

In a previous study (12), we observed a similar dependence of helix behavior on bilayer thickness in PDGFR-TMD; however, we used a somewhat shorter set of lipids in that study. Here, to compare the tilting behavior of E5 directly with the membrane alignment of PDGFR, we repeated the analysis of its TMD in the same lipid bilayers used for ΔE5, extending our previously published series to longer acyl chains. We found that the corresponding 1D^{15}N-NMR spectra of PDGFR-TMD had the same type of signals at ~200 ppm originating from the well-aligned protein fraction (Fig. 3). The contributions of misaligned and aggregated PDGFR-TMD in the upfield region of the spectrum at ~75 ppm, however, were obscured by signals of the isotropically averaged termini and by side-chain nitrogens, which are much more abundant in PDGFR (one Arg, three Lys, and one Gln) than in E5 (one Gln). Therefore, the upfield part of the NMR spectra could not be included in this lineshape deconvolution. The tilt angle of PDGFR-TMD determined by this fitting procedure was ~3° in DNPC, 10° in DErPC, 14° in DEiPC, and 22° in DOPC. These values and especially their trend are remarkably similar to the behavior seen for ΔE5 (Table 2). Compared with ΔE5, however, the PDGFR-TMD suffers less from aggregation, as the upfield ^{15}N-NMR signals (Fig. 3) are far less intense than those of E5 (Fig. 2).

2D-NMR: determination of the E5 helix tilt angle in bilayers of different thickness

2D solid-state NMR spectra can provide further resolution and additional spatial information regarding the secondary structure and orientation of membrane proteins. In separated local field experiments, helical proteins give rise to distinctive circular resonance patterns (so-called PISA wheels) that resemble their helical wheel projections (35–37). Most importantly, the position, size, and shape of a PISA wheel convey information about the orientation (tilt angle τ and azimuthal rotation angle ρ) of the aligned helix. Fig. 4 shows the ^{1}H-^{15}N SAMMY spectra of uniformly ^{15}N-labeled ΔE5 (using the same samples employed for the 1D measurements in Fig. 2) reconstituted in macroscopically aligned membranes of different thickness. In the very thick DNPC bilayers, a group of resonances is seen at ~180–230 ppm ^{15}N-CSA and 6–8.5 kHz in the dipolar coupling dimension (Fig. 4 *A*). The small area in which all resonances overlap is indicative of an almost upright transmembrane orientation of the helix, in good agreement with the OCD and 1D-NMR data described above. In moderately thick DErPC bilayers, the resonances are spread into a proper wheel-like pattern at lower ppm and kHz values, revealing a more tilted orientation of the E5 helix (Fig. 4 *B*). In DEiPC, the coherence of the wheel is lost, and the signals are spread out over several overlapping wheel-like patterns to even lower ppm and kHz values. At the same time, a new group of resonances emerges at ~50–150 ppm ^{15}N-CSA and 2–5 kHz dipolar coupling, representing the increasing fraction of aggregated E5 (Fig. 4 *C*). The spectrum in DOPC consists essentially of a powder spectrum caused by severe protein aggregation due to pronounced hydrophobic mismatch in these conventional lipid bilayers (Fig. 4 *D*).

The tilt angle of ΔE5 in the different lipid systems can be deduced by fitting the experimental spectra with simulated PISA wheels (50,67). In Fig. 4, *A–C*, the best-fit PISA wheels (calculated for an ideal α-helix) are shown

TABLE 2 Orientation of ΔE5 and PDGFR-TMD in Lipid Bilayers

Protein	Measurement	Parameter	DNPC	DErPC	DEiPC	DOPC
ΔE5	1D	τ_{obs} (°)	12 ± 3	14 ± 2	17 ± 1	21 ± 5
		f_{agg} (%)	13 ± 7	10 ± 3	35 ± 5	77 ± 8
		S_{mol}	0.95 ± 0.04	0.97 ± 0.02	0.95 ± 0.03	0.96 ± 0.04
	2D	τ_{obs} (°)	0–10 + 2	12 ± 2	15 ± 5	n.d.
		S_{mol}	0.97	0.97	0.97	n.d.
PDGFR-TMD	1D	τ_{obs} (°)	3	10	14	22
		f_{agg} (%)	2[a]	0[a]	0[a]	0[a]
		S_{mol}	1	1	1	1

The helix tilt angle (τ_{obs}, defined with respect to the membrane normal), the fraction of aggregated protein (f_{agg}), and the order parameter (S_{mol}) were estimated from 1D- and 2D-NMR spectra. For ΔE5, the averaged results of three individual samples per lipid are shown (see Table S1 for detailed results). n.d., not determined.

[a]For PDGFR-TMD, the f_{agg} is less reliable (see text and Fig. 3).

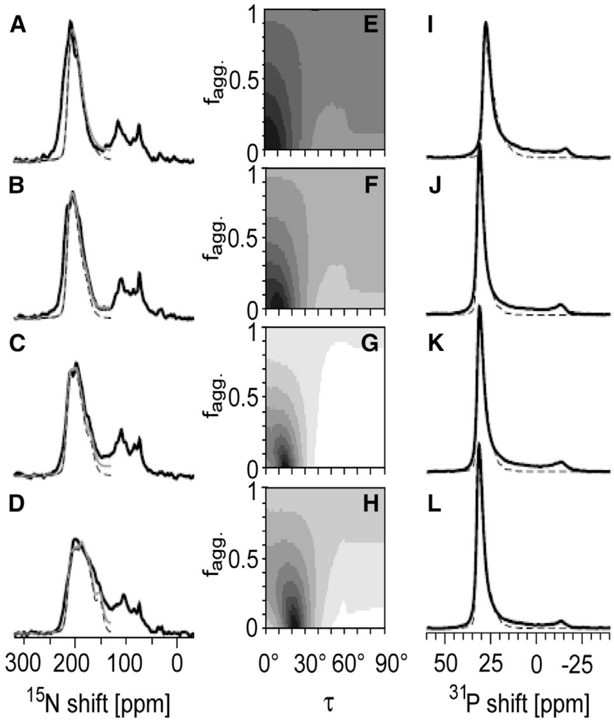

FIGURE 3 1D solid-state ^{15}N- and ^{31}P-NMR analyses of the membrane alignment and aggregation tendency of PDGFR-TMD, in a lipid series analogous to that used for ΔE5 (see Fig. 2). (*A–L*) ^{15}N-NMR (*A–D*) and ^{31}P-NMR (*I–L*) lineshape analyses were carried out in DNPC (*A*, *E*, and *I*), DErPC (*B*, *F*, and *J*), DEiPC (*C*, *G*, and *K*), and DOPC (*D*, *H*, and *L*). The tilt angle of PDGFR-TMD was found to behave very similarly to that of E5 in all membranes studied here, although the protein had a less pronounced tendency to aggregate (see Table 2).

superimposed on the experimental data. For ΔE5 in DNPC, any PISA wheel with a tilt angle τ between 0° and 10° + 2° matches the observed resonances well (Table 2). Due to signal overlap, it is not possible to determine the tilt angle more exactly. For DErPC, a simulated PISA wheel with a tilt angle of 12° ± 2° fits the observed NMR data very well, in full agreement with the 1D-NMR data. In DEiPC, the assignment of a single, uniform PISA wheel is hampered by the broad distribution of resonances. However, PISA wheels with tilt angles covering a range from 10° to 20° match the observed signals well, which is indicative of some helix distortion such as bending or a kink. In DOPC, a PISA wheel analysis does not make sense due to the lack of properly reconstituted protein. The order parameters obtained from fitting the series of SAMMY spectra support those determined from the 1D-NMR measurements. Overall, the 2D-NMR measurements contain more information and are more accurate, and fully confirm the results of the 1D-NMR analyses. The structural responses of E5 are summarized in Fig. 6, which illustrates how E5 adapts to an increasing hydrophobic mismatch: upon going from DNPC to DOPC, the helix adjusts its tilt angle, then suffers from some distortion, and finally loses its membrane alignment completely and forms helical aggregates.

1D-NMR: orientation of PDGFR and E5 in a heterooligomeric complex

Next, we determined the alignment of both E5 and PDGFR in a biologically relevant heterooligomeric complex to examine their influence on each other. We reconstituted both proteins together in lipid membranes using an equimolar ratio of labeled ^{15}N-PDGFR-TMD and unlabeled ^{14}N-ΔE5, and, in a reverse experiment, some labeled ^{15}N-ΔE5 and unlabeled ^{14}N-PDGFR-TMD. These ^{15}N/^{14}N mixtures were reconstituted in thick DErPC and thin DOPC bilayers, and the orientation of either protein was determined by solid-state 1D-NMR. When ^{15}N-PDGFR-TMD and ^{14}N-ΔE5 were reconstituted together in thick DErPC bilayers, the observed 1D-NMR spectrum perfectly matched the spectrum of pure PDGFR-TMD (Figs. 5 *A* and S6), indicating that the orientation of PDGFR does not change due to the presence of E5. Accordingly, spectral deconvolution yielded a tilt angle of 12° ± 2°, which is similar to the tilt found for PDGFR-TMD alone (Tables 3 and S2). In the reverse experiment, when ^{15}N-ΔE5 was reconstituted together with ^{14}N-PDGFR-TMD in DErPC, the lineshape was the same as that observed for the single protein alone (Figs. 5 *B* and S7), indicating that the transmembrane orientation of ΔE5 in thick membranes is not affected by the presence of PDGFR. Here, a tilt angle of 14° ± 0° was found, which is close to the tilt measured for the pure protein in the same lipid bilayer. When ^{15}N-PDGFR-TMD and ^{14}N-ΔE5 were reconstituted in thin DOPC membranes, the slightly tilted orientation of the protein was not affected by the presence of ΔE5 either (Figs. 5 *C* and S8), as the tilt angle of 23° ± 1° is the same as that observed for the PDGFR-TMD alone. Most interestingly, however, the 1D spectrum of the ^{15}N-ΔE5/^{14}N-PDGFR-TMD complex in DOPC reveals a complete change in the lineshape compared with the spectrum of pure ΔE5 (Figs. 5 *D* and S9). Now, a signal originating from well-reconstituted E5 in a proper transmembrane alignment is seen in the downfield part of the spectrum, whereas E5 alone in DOPC had been mostly aggregated (Fig. 2 *D*). Obviously, the instability of E5 due to hydrophobic mismatch in these comparatively thin bilayers was reversed by the presence of PDGFR. The receptor clearly promoted the successful reconstitution of E5 and stabilized its proper transmembrane alignment with a tilt angle of 7° ± 1°. These data suggest that PDGFR and E5 actively assemble into a heterooligomeric complex in DOPC membranes, allowing ΔE5 to overcome the pronounced hydrophobic mismatch by binding to PDGFR and adopting an upright orientation.

DISCUSSION

The E5 oncoprotein, with a length of only 44 amino acids, is an integral membrane protein with remarkable features. It is mostly hydrophobic, but is highly specific in that it can

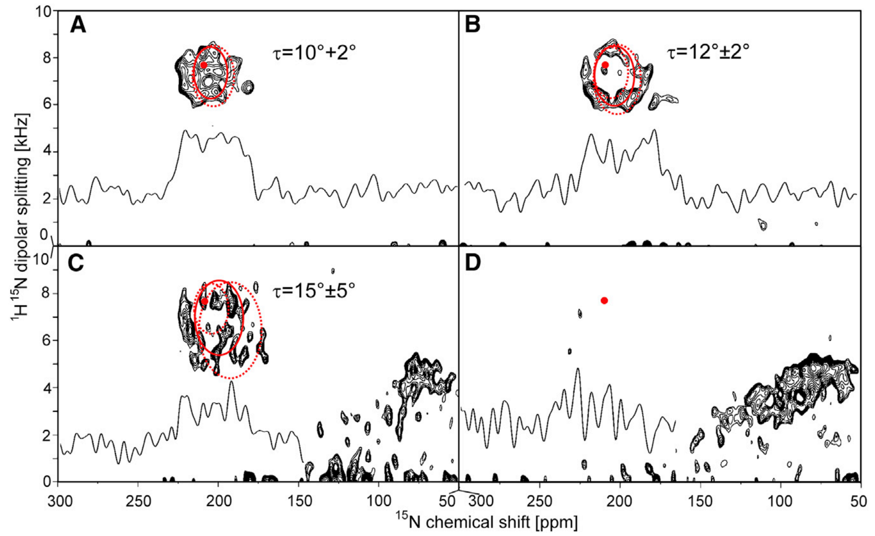

FIGURE 4 2D solid-state NMR analysis of uniformly ^{15}N-labeled ΔE5 in macroscopically aligned membranes of different thickness. Experimental ^{1}H-^{15}N SAMMY spectra were fitted with simulated PISA wheels (*full circles*) using the experimentally determined ^{15}N-CSA tensor, dipolar half splitting, and an order parameter S_{mol} = 0.97. In each spectrum, the 1D projection of the ^{15}N chemical shift (extracted at 7.5 kHz in the ^{1}H^{15}N dipolar coupling dimension) is shown as a black line to illustrate the signal/noise ratio. (*A–C*) To estimate errors in the tilt-angle determination, PISA wheels that just fit to the observed signals were added (*dotted circles*). The position of the 0° PISA wheel is shown for orientation as a red spot. (*A*) In DNPC, the observed resonance pattern is well reproduced by simulated PISA wheels with tilt angles τ between 0° and 10° + 2°. (*B*) In DErPC, the experimental data are fitted best with a simulated PISA wheel of τ = 12° ± 2°. (*C*) In DEiPC, PISA wheels with a tilt angle of τ = 15° ± 5° cover the observed signals, suggesting a slight kink or a bent helix. (*D*) In DOPC, the protein could not be reconstituted and gave a broad powder distribution of signals.

activate only PDGFR β, and no other closely related RTKs (17,18,68). This point is even more astonishing in the light of numerous reports that the specific sequence is not critical for the biological activity of E5, as most of the hydrophobic amino acids can be conservatively substituted without any loss of function (69–72). Hence, features other than the actual sequence must play a decisive role in the recognition of PDGFR, such as the presence and position of two polar key residues (Gln-17 in the TMD and Asp-33 in the juxtamembrane region), the hydrophobic dimer interface on the E5 helix that drives its self-assembly, and the actual length of the TMD and/or its orientation in the lipid membrane. The lipid bilayer itself may also have an influence on the E5 protein and its ability to interact with itself and with PDGFR. Here, we found that the conformation, orientation, and stability of the E5 protein are indeed affected by the lipid bilayer and by the presence of PDGFR.

Using solid-state NMR, SRCD, and OCD, we found that ΔE5 can be stably incorporated into very thick lipid bilayers (e.g., DNPC), but in conventional membranes (e.g., DOPC) it aggregates severely due to hydrophobic mismatch. When inserted into thick planar lipid membranes, the protein adopts the same α-helical conformation that has previously been observed in detergent micelles and DMPC bilayers (22,24,25,28). For ΔE5, the total helix content of 91% in DNPC and 89% in DErPC is slightly higher than that in detergent micelles (82%) (28), indicating that an appropriate lipid environment stabilizes the transmembrane helix by roughly one additional turn. Notably, ~73% of ΔE5 was found to be in an ideal helix conformation (α_R content) in DNPC. This percentage is equivalent to a stretch of ~26 amino acids, which fits remarkably well with the length of the hydrophobic sequence flanked by Trp-5 and Trp-32. Tryptophan and to a lesser extent other aromatic side chains are typically located in the amphiphilic interface regions of transmembrane segments, where they serve as anchors to hold the membrane proteins at a well-defined position within the lipid bilayer (67,73–77). In E5, there are additional anchoring residues (Phe-6 and Tyr-31) right next to the two prominent tryptophans at either end of the TMD, which contribute to a strong anchoring effect on each face of the bilayer. The E5 helix also extends beyond the membrane, though with a less regular conformation, as reflected by the distorted helix content αD, and the C- and N-termini are unstructured.

Clearly, the TMD in E5 with 28 residues (24 amino acids + 4 anchoring ones) is much longer than the typical stretch of ~20 hydrophobic amino acids that is commonly expected for single-span integral membrane proteins (78). PDGFR also has a remarkably long membrane-spanning domain of 27 residues (25 amino acids + 2 anchoring ones) (12), and our ^{15}N-NMR analyses yielded comparable helix tilt angles for both ΔE5 and the TMD of the receptor. Thus, it is tempting to speculate that E5 has evolved into a 3D structure that is perfectly matched to the length and orientation of the PDGFR transmembrane segment. A recent study showed that randomized TMDs can also activate PDGFR, although they do not contain any sequences derived from the E5 protein (79). Interestingly, the common feature that these peptides share with the E5 protein is an unusually long hydrophobic stretch of 25 amino acids, indicating that indeed the extreme length of the E5 TMD is critical for interaction with PDGFR. In this way, the key residue in the middle of the E5 helix, Gln-17, will always be positioned at the right depth to interact with the central threonine on PDGFR. The second key residue on E5, Asp-33,

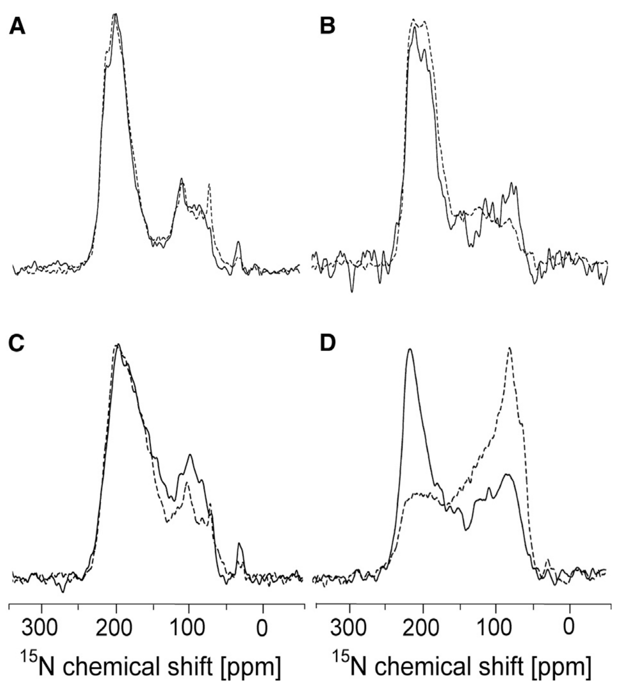

FIGURE 5 (*A–D*) 1D solid-state ^{15}N-NMR analysis of the membrane alignment of PDGFR-TMD and ΔE5 under the influence of each other in thick DErPC (*A* and *B*) and thin DOPC (*C* and *D*) lipid bilayers. The spectra of the hetero mixtures (*solid lines*) are superimposed with the corresponding spectra of the pure ^{15}N-labeled ΔE5 and PDGFR-TMD (*dashed lines*), taken from Figs. 2 and 3, respectively. (*A* and *C*) For the ^{15}N-PDGFR-TMD/^{14}N-ΔE5 complex, no changes compared with the 1D spectra of the pure ^{15}N-PDGFR-TMD were observed, indicating that the alignment of the protein is not affected by the presence of ΔE5. (*B* and *D*) For the ^{15}N-ΔE5/^{14}N-PDGFR-TMD complex, no changes were observed in DErPC; however, in DOPC, the presence of PDGFR-TMD promoted the reconstitution and induced a transmembrane orientation of ΔE5.

lies in the region where the helix starts to unwind; therefore, it is not necessarily located on the opposite face of the helix relative to Gln-17, as was previously proposed for the *cis* model of the E5/PDGFR complex (22). Here, we have demonstrated that the orientation of E5 is sensitive to the bilayer thickness, as was previously observed for PDGFR. In very thick DNPC membranes, both proteins are inserted almost upright, as the hydrophobic length of the transmembrane segments matches the hydrophobic thickness of the bilayer (Fig. 6). However, in thinner membranes, E5 is

TABLE 3 Orientation of ΔE5 and PDGFR-TMD in the Heterooligomeric Complex

Mixture	Parameter	DErPC	DOPC
^{15}N-ΔE5/^{14}N-PDGFR-TMD (1:1)	τ_{obs} (°)	14 ± 0	7 ± 1
	f_{agg} (%)	3 ± 3	3 ± 5
	S_{mol}	0.91 ± 0.01	0.97 ± 0.03
^{15}N-PDGFR-TMD/^{14}N-ΔE5 (1:1)	τ_{obs} (°)	12 ± 2	23 ± 1
	f_{agg} (%)	0[a]	0[a]
	S_{mol}	1	1

Averaged results per lipid are shown (see Tables 2 and S2 for details).
[a]For PDGFR-TMD, the f_{agg} is less reliable (see text).

under considerable conformational strain when it tries to adapt to the reduced bilayer thickness. Helix tilting in DErPC is seen from the shift of the PISA wheel in the SAMMY spectrum. Some additional helix bending or kink formation is observed in DEiPC, and in DOPC the PISA wheel has totally disintegrated. PISA wheel measurements are very sensitive to variations of the backbone Φ, Ψ torsion angles, and even minor changes have a strong impact on the pattern of the PISA wheel. For example, previous spectral simulations of an 18-residue helical peptide showed a distinct PISA wheel when standard torsion angles were used, but the pattern essentially disappeared when the torsion angles were varied (67). Interestingly, the helix tilt angles observed for ΔE5 in DErPC, DEiPC, and DOPC are significantly smaller than one would expect based on geometrical arguments. To completely immerse the E5 TMD within the membrane, one would expect to find a tilt angle of 28° in DErPC (instead of the observed 14°), 38° in DEiPC (observed 17°), and 47° in DOPC (observed 21°). Thus, E5 can tilt to a certain extent, but then the protein aggregates because it is unable to tilt any more. Therefore, the observed moderate tilting of E5 does not fully compensate for the hydrophobic mismatch experienced by the protein: there remains a nominal mismatch for the actual length of the TMD of 3.4 Å in DErPC, 6.8 Å in DEiPC, and 9.6 Å in DOPC. Interestingly, in a considerably thinner DMPC bilayer, a maximum helix tilt of only ~20° was previously reported by infrared measurements (22). On the other hand, an ideal helix of 26 amino acids for the TMD of E5 corresponds to a length of ~39 Å, which is well matched to the hydrophobic thickness of 38.2 Å of DNPC. Accordingly, the observed tilt angle of 12° in DNPC fits the expected value.

The finding that ΔE5 is not able to compensate for hydrophobic mismatch by tilting is a strong argument for the presence of homodimers (and higher oligomers), despite the fact that the truncated ΔE5 does not contain any cysteines. A single transmembrane segment would be much more sensitive to the bilayer thickness than a dimer or an oligomeric bundle, as has been documented for various monomeric transmembrane helices (39,80–83). Interestingly, even when we diluted the protein in the membrane to promote disassembly of the noncovalent dimer, we observed no changes in the 1D-NMR spectra for either thick or thin membranes (Fig. S10). We may thus conclude that helix-helix interactions lock ΔE5 together as a homodimer, thereby enforcing a relative upright orientation of the bundle. A similar orientational behavior was previously reported for the tetrameric M2 proton channel (84) and for the diacylglycerol kinase, a three-TM-helix membrane protein (67), in which specific electrostatic interactions lock the protein into a more rigid conformation so that it does not respond to changes in the bilayer thickness. Given that the E5 TMD is known to self-assemble per se in lipid membranes (28,85), the corresponding helix-helix interactions must be

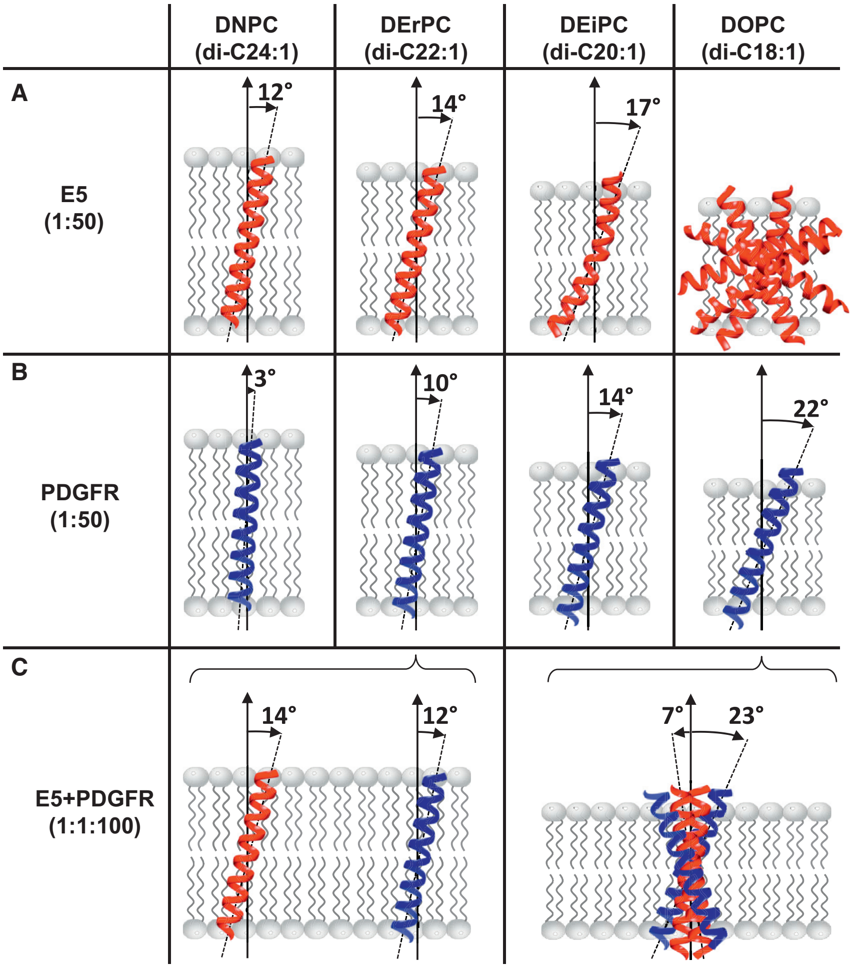

FIGURE 6 (*A–C*) Model of the orientational behavior of the E5 protein (*A*), the TMD of PDGFR (*B*), and the PDGFR/E5 heterooligomeric complex (*C*) in lipid bilayers of different thickness, reconstituted with peptide/lipid ratios as indicated. (*A*) Upon going from very thick DNPC membranes to common bilayers (such as DOPC, POPC, and DMPC), the unusually long E5 helix has to adapt to the increasing hydrophobic mismatch. While being oriented essentially upright (in DNPC), it first adjusts its tilt angle (in DErPC), then suffers from helix distortion (in DEiPC), and finally loses its membrane alignment and forms helical aggregates (in DOPC). (*B*) The TMD of PDGFR adjusts its tilt angle in response to the bilayer thickness. (*C*) The E5 protein can compensate for the hydrophobic mismatch in thin DOPC bilayers (such as the membranes of the Golgi compartment) by binding to the TMD of the monomeric receptor, thereby clustering the receptor in its active conformation. In thick DErPC bilayers (such as in the plasma membrane), both proteins remain largely on their own. Here, the hydrophobic mismatch is less critical, and thus E5 can reside stably in the bilayer without binding to the receptor. Molecular graphics were performed with the UCSF Chimera package (89).

quite strong and specific to hold the dimer together and force the protein into a more upright orientation. Besides the hydrophobic interface, in the full-length wild-type the two cysteine residues would contribute by forming a covalent link. The biological function of the E5 protein obviously relies on dimerization to maintain a specific conformation in the membrane, enabling it to interact with PDGFR and other cellular targets.

The solid-state ^{15}N-NMR analyses presented here clearly demonstrate that the structural behavior of E5 in membranes is sensitive not only to the bilayer thickness but also to the presence of PDGFR. When both proteins were reconstituted by themselves in comparatively thin DOPC bilayers, PDGFR-TMD was well reconstituted, whereas ΔE5 showed severe protein aggregation due to hydrophobic mismatch and its inability to tilt. On the other hand, when the two proteins were reconstituted together, the presence of PDGFR greatly stabilized ΔE5, resulting in a well-oriented alignment of the protein. A recent study found that PDGFR-TMD is already present as preformed dimers in thick lipid membranes, but in thinner membranes, where the protein has to be more tilted, these dimers tend to disassemble (12). We thus conclude that in thin bilayers, E5 binds to the tilted and monomeric TMD of PDGFR to counteract the hydrophobic mismatch (Fig. 6). The need to avoid hydrophobic mismatch may thus be the underlying driving force for the long-range recognition and binding of E5 to the receptor. Once the proteins have approached each other, specific polar and hydrogen (H)-bonded interactions will determine their precise docking in the short range.

In living cells, E5 is known to be localized in the thin membranes of the endoplasmic reticulum (ER) and *cis*-Golgi compartment, where it interacts with the immature and mature forms of PDGFR, whereas only low amounts are detected in the plasma membrane (29,30,86,87). Our data can now explain why and how the intimate interaction of E5 with PDGFR can lead to retention of the complex in the thinner membranes of the ER and Golgi, as the pronounced hydrophobic mismatch becomes less unfavorable for the heterooligomeric bundle compared with the single proteins per se. In this way, it is likely that the subunits of the receptor are clustered by E5 into a functional signal transduction complex in the thin membranes of the ER and Golgi, but when PDGFR is on its own, premature receptor dimerization and activation are prevented by the pronounced hydrophobic mismatch. On the other hand, in

thick bilayers (such as in the plasma membrane), no changes were observed in the NMR spectra of the complex compared with the pure proteins, indicating that both proteins remained largely on their own. Here, the hydrophobic mismatch is less critical, and E5 can thus reside stably in the bilayer without binding to the receptor.

In the current mechanistic models for signaling, RTKs are activated upon ligand binding, which results in receptor dimerization followed by activation of the tyrosine kinase domains. Over the last decade, however, this simple model has been challenged by reports that the TMDs of RTKs play a critical role in regulating dimerization. Many RTKs were found to be present as preformed dimers even before ligand binding, such as in the case of PDGFR (12), the EGF receptor (8), the Neu receptor (11), and the Erythropoietin receptor (9,10). However, these preformed dimers are supposed to represent an inactive state in which the subunits are oriented nonfunctionally with respect to one another (see previous studies (6,9,88) for a model of receptor activation). Ligand binding is then supposed to induce a rotation of the subunits toward their active conformation, which brings the catalytic domains into close proximity to allow transphosphorylation and receptor activation. A rotational repositioning of the receptor subunits has also been shown for PDGFR, suggesting that the receptor is active only when the monomers face each other in a specific conformation (11). Together, these findings highlight the role of the TMD as a molecular switch in the activation of PDGFR. It is tempting to speculate that the E5 protein provides a favorable scaffold within the otherwise unfavorable membrane environment of the Golgi compartment, which is beneficial for receptor clustering and for rotational repositioning of the transmembrane segments into their active dimer conformation. In this way, PDGFR can become activated already in the Golgi compartment and engage in signaling without ever reaching the cell surface or encountering a growth factor.

SUPPORTING MATERIAL

Supporting Materials and Methods, ten figures, and two tables are available at http://www.biophysj.org/biophysj/supplemental/S0006-3495(15)00728-6.

AUTHOR CONTRIBUTIONS

D.W. coordinated the project, performed SRCD/OCD measurements, and wrote the manuscript. C.Z. and M.Z. performed NMR measurements. S.L.G. analyzed the NMR data. J.B. analyzed the SRCD/OCD data. P.L.G. designed and developed the NMR equipment used in this study. A.S.U. served as the scientific coordinator and corrected the manuscript.

ACKNOWLEDGMENTS

We thank the Synchrotron Light Source ANKA for providing beam time at the UV-CD12 beamline, and Siegmar Roth and Bianca Posselt for technical assistance in using the beamline and the sample preparation lab. We also thank Markus Schmitt for his help in the NMR facility, and Christian Weber and Stefanie Vollmer for their support in producing the recombinant proteins.

Molecular graphics and analyses were performed with the UCSF Chimera package. Chimera was developed by the Resource for Biocomputing, Visualization, and Informatics at the University of California, San Francisco (supported by NIGMS P41-GM103311).

SUPPORTING CITATIONS

References (90–94) appear in the Supporting Material.

REFERENCES

1. Killian, J. A. 1998. Hydrophobic mismatch between proteins and lipids in membranes. *Biochim. Biophys. Acta.* 1376:401–415.
2. Jensen, M. Ø., and O. G. Mouritsen. 2004. Lipids do influence protein function—the hydrophobic matching hypothesis revisited. *Biochim. Biophys. Acta.* 1666:205–226.
3. Coskun, U., and K. Simons. 2011. Cell membranes: the lipid perspective. *Structure.* 19:1543–1548.
4. Sparr, E., W. L. Ash, …, J. A. Killian. 2005. Self-association of transmembrane alpha-helices in model membranes: importance of helix orientation and role of hydrophobic mismatch. *J. Biol. Chem.* 280:39324–39331.
5. Andrae, J., R. Gallini, and C. Betsholtz. 2008. Role of platelet-derived growth factors in physiology and medicine. *Genes Dev.* 22:1276–1312.
6. Lemmon, M. A., and J. Schlessinger. 2010. Cell signaling by receptor tyrosine kinases. *Cell.* 141:1117–1134.
7. Shim, A. H., H. Liu, …, X. He. 2010. Structures of a platelet-derived growth factor/propeptide complex and a platelet-derived growth factor/receptor complex. *Proc. Natl. Acad. Sci. USA.* 107:11307–11312.
8. Moriki, T., H. Maruyama, and I. N. Maruyama. 2001. Activation of preformed EGF receptor dimers by ligand-induced rotation of the transmembrane domain. *J. Mol. Biol.* 311:1011–1026.
9. Jiang, G., and T. Hunter. 1999. Receptor signaling: when dimerization is not enough. *Curr. Biol.* 9:R568–R571.
10. Livnah, O., E. A. Stura, …, I. A. Wilson. 1999. Crystallographic evidence for preformed dimers of erythropoietin receptor before ligand activation. *Science.* 283:987–990.
11. Bell, C. A., J. A. Tynan, …, D. J. Donoghue. 2000. Rotational coupling of the transmembrane and kinase domains of the Neu receptor tyrosine kinase. *Mol. Biol. Cell.* 11:3589–3599.
12. Muhle-Goll, C., S. Hoffmann, …, A. S. Ulrich. 2012. Hydrophobic matching controls the tilt and stability of the dimeric platelet-derived growth factor receptor (PDGFR) β transmembrane segment. *J. Biol. Chem.* 287:26178–26186.
13. Petti, L., L. A. Nilson, and D. DiMaio. 1991. Activation of the platelet-derived growth factor receptor by the bovine papillomavirus E5 transforming protein. *EMBO J.* 10:845–855.
14. Petti, L., and D. DiMaio. 1992. Stable association between the bovine papillomavirus E5 transforming protein and activated platelet-derived growth factor receptor in transformed mouse cells. *Proc. Natl. Acad. Sci. USA.* 89:6736–6740.
15. Goldstein, D. J., T. Andresson, …, R. Schlegel. 1992. The BPV-1 E5 protein, the 16 kDa membrane pore-forming protein and the PDGF receptor exist in a complex that is dependent on hydrophobic transmembrane interactions. *EMBO J.* 11:4851–4859.
16. Nilson, L. A., and D. DiMaio. 1993. Platelet-derived growth factor receptor can mediate tumorigenic transformation by the bovine papillomavirus E5 protein. *Mol. Cell. Biol.* 13:4137–4145.
17. Goldstein, D. J., W. Li, …, J. H. Pierce. 1994. The bovine papillomavirus type 1 E5 transforming protein specifically binds and activates the

beta-type receptor for the platelet-derived growth factor but not other related tyrosine kinase-containing receptors to induce cellular transformation. *J. Virol.* 68:4432–4441.

18. Petti, L., and D. DiMaio. 1994. Specific interaction between the bovine papillomavirus E5 transforming protein and the beta receptor for platelet-derived growth factor in stably transformed and acutely transfected cells. *J. Virol.* 68:3582–3592.
19. Lai, C. C., C. Henningson, and D. DiMaio. 1998. Bovine papillomavirus E5 protein induces oligomerization and trans-phosphorylation of the platelet-derived growth factor beta receptor. *Proc. Natl. Acad. Sci. USA.* 95:15241–15246.
20. Lai, C. C., C. Henningson, and D. DiMaio. 2000. Bovine papillomavirus E5 protein induces the formation of signal transduction complexes containing dimeric activated platelet-derived growth factor beta receptor and associated signaling proteins. *J. Biol. Chem.* 275:9832–9840.
21. Goldstein, D. J., R. Kulke, …, R. Schlegel. 1992. A glutamine residue in the membrane-associating domain of the bovine papillomavirus type 1 E5 oncoprotein mediates its binding to a transmembrane component of the vacuolar H(+)-ATPase. *J. Virol.* 66:405–413.
22. Surti, T., O. Klein, …, S. O. Smith. 1998. Structural models of the bovine papillomavirus E5 protein. *Proteins.* 33:601–612.
23. Klein, O., D. Kegler-Ebo, …, D. DiMaio. 1999. The bovine papillomavirus E5 protein requires a juxtamembrane negative charge for activation of the platelet-derived growth factor beta receptor and transformation of C127 cells. *J. Virol.* 73:3264–3272.
24. Oates, J., M. Hicks, …, A. M. Dixon. 2008. In vitro dimerization of the bovine papillomavirus E5 protein transmembrane domain. *Biochemistry.* 47:8985–8992.
25. King, G., J. Oates, …, A. M. Dixon. 2011. Towards a structural understanding of the smallest known oncoprotein: investigation of the bovine papillomavirus E5 protein using solution-state NMR. *Biochim. Biophys. Acta.* 1808:1493–1501.
26. Mattoon, D., K. Gupta, …, D. DiMaio. 2001. Identification of the transmembrane dimer interface of the bovine papillomavirus E5 protein. *Oncogene.* 20:3824–3834.
27. Talbert-Slagle, K., S. Marlatt, …, D. Dimaio. 2009. Artificial transmembrane oncoproteins smaller than the bovine papillomavirus E5 protein redefine sequence requirements for activation of the platelet-derived growth factor beta receptor. *J. Virol.* 83:9773–9785.
28. Windisch, D., C. Ziegler, …, A. S. Ulrich. 2014. Structural characterization of a C-terminally truncated E5 oncoprotein from papillomavirus in lipid bilayers. *Biol. Chem.* 395:1443–1452.
29. Burkhardt, A., M. Willingham, …, R. Schlegel. 1989. The E5 oncoprotein of bovine papillomavirus is oriented asymmetrically in Golgi and plasma membranes. *Virology.* 170:334–339.
30. Sparkowski, J., J. Anders, and R. Schlegel. 1995. E5 oncoprotein retained in the endoplasmic reticulum/cis Golgi still induces PDGF receptor autophosphorylation but does not transform cells. *EMBO J.* 14:3055–3063.
31. Windisch, D., S. Hoffmann, …, A. S. Ulrich. 2010. Structural role of the conserved cysteines in the dimerization of the viral transmembrane oncoprotein E5. *Biophys. J.* 99:1764–1772.
32. Waugh, J. S. 1976. Uncoupling of local field spectra in nuclear magnetic resonance: determination of atomic positions in solids. *Proc. Natl. Acad. Sci. USA.* 73:1394–1397.
33. Wang, J., J. Denny, …, T. A. Cross. 2000. Imaging membrane protein helical wheels. *J. Magn. Reson.* 144:162–167.
34. Nevzorov, A. A., and S. J. Opella. 2003. A “magic sandwich” pulse sequence with reduced offset dependence for high-resolution separated local field spectroscopy. *J. Magn. Reson.* 164:182–186.
35. Marassi, F. M., and S. J. Opella. 2003. Simultaneous assignment and structure determination of a membrane protein from NMR orientational restraints. *Protein Sci.* 12:403–411.
36. Ramamoorthy, A., Y. Wei, and D.-K. Lee. 2004. PISEMA solid-state NMR spectroscopy. *Annu. Rep. NMR Spectrosc.* 52:1–52.
37. Opella, S. J., and F. M. Marassi. 2004. Structure determination of membrane proteins by NMR spectroscopy. *Chem. Rev.* 104:3587–3606.
38. Marassi, F. M., and S. J. Opella. 2000. A solid-state NMR index of helical membrane protein structure and topology. *J. Magn. Reson.* 144:150–155.
39. Park, S. H., and S. J. Opella. 2005. Tilt angle of a trans-membrane helix is determined by hydrophobic mismatch. *J. Mol. Biol.* 350:310–318.
40. Wang, J., S. Kim, …, T. A. Cross. 2001. Structure of the transmembrane region of the M2 protein H(+) channel. *Protein Sci.* 10:2241–2250.
41. Bürck, J., S. Roth, …, A. S. Ulrich. 2008. Conformation and membrane orientation of amphiphilic helical peptides by oriented circular dichroism. *Biophys. J.* 95:3872–3881.
42. Clarke, D. T., and G. Jones. 2004. CD12: a new high-flux beamline for ultraviolet and vacuum-ultraviolet circular dichroism on the SRS, Daresbury. *J. Synchrotron Radiat.* 11:142–149.
43. Bürck, J., S. Roth, …, A. S. Ulrich. 2015. UV-CD12: synchrotron radiation circular dichroism beamline at ANKA. *J. Synchrotron Radiat.* 22:844–852.
44. Sreerama, N., and R. W. Woody. 2000. Estimation of protein secondary structure from circular dichroism spectra: comparison of CONTIN, SELCON, and CDSSTR methods with an expanded reference set. *Anal. Biochem.* 287:252–260.
45. van Stokkum, I. H., H. J. Spoelder, …, F. C. Groen. 1990. Estimation of protein secondary structure and error analysis from circular dichroism spectra. *Anal. Biochem.* 191:110–118.
46. Whitmore, L., and B. A. Wallace. 2004. DICHROWEB, an online server for protein secondary structure analyses from circular dichroism spectroscopic data. *Nucleic Acids Res.* 32:W668–W673.
47. Lobley, A., L. Whitmore, and B. A. Wallace. 2002. DICHROWEB: an interactive website for the analysis of protein secondary structure from circular dichroism spectra. *Bioinformatics.* 18:211–212.
48. Whitmore, L., B. Woollett, …, B. A. Wallace. 2011. PCDDB: the Protein Circular Dichroism Data Bank, a repository for circular dichroism spectral and metadata. *Nucleic Acids Res.* 39:D480–D486.
49. Gor’kov, P. L., E. Y. Chekmenev, …, W. W. Brey. 2007. Using low-E resonators to reduce RF heating in biological samples for static solid-state NMR up to 900 MHz. *J. Magn. Reson.* 185:77–93.
50. Walther, T. H., S. L. Grage, …, A. S. Ulrich. 2010. Membrane alignment of the pore-forming component TatA(d) of the twin-arginine translocase from Bacillus subtilis resolved by solid-state NMR spectroscopy. *J. Am. Chem. Soc.* 132:15945–15956.
51. Marsh, D. 2008. Energetics of hydrophobic matching in lipid-protein interactions. *Biophys. J.* 94:3996–4013.
52. Wallace, B. A., and D. Mao. 1984. Circular dichroism analyses of membrane proteins: an examination of differential light scattering and absorption flattening effects in large membrane vesicles and membrane sheets. *Anal. Biochem.* 142:317–328.
53. Teeters, C. L., J. Eccles, and B. A. Wallace. 1987. A theoretical analysis of the effects of sonication on differential absorption flattening in suspensions of membrane sheets. *Biophys. J.* 51:527–532.
54. Nolandt, O. V., T. H. Walther, …, A. S. Ulrich. 2009. Structure analysis of the membrane protein TatC(d) from the Tat system of B. subtilis by circular dichroism. *Biochim. Biophys. Acta.* 1788:2238–2244.
55. Wadhwani, P., J. Bürck, …, A. S. Ulrich. 2008. Using a sterically restrictive amino acid as a 19F NMR label to monitor and to control peptide aggregation in membranes. *J. Am. Chem. Soc.* 130:16515–16517.
56. Strandberg, E., N. Kanithasen, …, A. S. Ulrich. 2008. Solid-state NMR analysis comparing the designer-made antibiotic MSI-103 with its parent peptide PGLa in lipid bilayers. *Biochemistry.* 47:2601–2616.
57. Lange, C., S. D. Müller, …, A. S. Ulrich. 2007. Structure analysis of the protein translocating channel TatA in membranes using a multi-construct approach. *Biochim. Biophys. Acta.* 1768:2627–2634.

58. Steinbrecher, T., S. Prock, …, A. S. Ulrich. 2012. Peptide-lipid interactions of the stress-response peptide TisB that induces bacterial persistence. *Biophys. J.* 103:1460–1469.
59. Klein, M. J., S. L. Grage, …, A. S. Ulrich. 2012. Structure analysis of the membrane-bound PhoD signal peptide of the Tat translocase shows an N-terminal amphiphilic helix. *Biochim. Biophys. Acta.* 1818:3025–3031.
60. Wadhwani, P., E. Strandberg, …, A. S. Ulrich. 2012. Self-assembly of flexible β-strands into immobile amyloid-like β-sheets in membranes as revealed by solid-state 19F NMR. *J. Am. Chem. Soc.* 134:6512–6515.
61. Heinzmann, R., S. L. Grage, …, A. S. Ulrich. 2011. A kinked antimicrobial peptide from Bombina maxima. II. Behavior in phospholipid bilayers. *Eur. Biophys. J.* 40:463–470.
62. Wadhwani, P., E. Strandberg, …, A. S. Ulrich. 2014. Dynamical structure of the short multifunctional peptide BP100 in membranes. *Biochim. Biophys. Acta.* 1838:940–949.
63. Aberle, D., C. Muhle-Goll, …, G. Meyers. 2014. Structure of the membrane anchor of pestivirus glycoprotein E(rns), a long tilted amphipathic helix. *PLoS Pathog.* 10:e1003973.
64. Fanghänel, S., P. Wadhwani, …, A. S. Ulrich. 2014. Structure analysis and conformational transitions of the cell penetrating peptide transportan 10 in the membrane-bound state. *PLoS One.* 9:e99653.
65. Olah, G. A., and H. W. Huang. 1988. Circular dichroism of oriented α-helices. II. Electric field oriented polypeptides. *J. Chem. Phys.* 89:6956–6962.
66. Wu, Y., H. W. Huang, and G. A. Olah. 1990. Method of oriented circular dichroism. *Biophys. J.* 57:797–806.
67. Page, R. C., C. Li, …, T. A. Cross. 2007. Lipid bilayers: an essential environment for the understanding of membrane proteins. *Magn. Reson. Chem.* 45 (*Suppl 1*):S2–S11.
68. Staebler, A., J. H. Pierce, …, D. J. Goldstein. 1995. Mutational analysis of the beta-type platelet-derived growth factor receptor defines the site of interaction with the bovine papillomavirus type 1 E5 transforming protein. *J. Virol.* 69:6507–6517.
69. Horwitz, B. H., A. L. Burkhardt, …, D. DiMaio. 1988. 44-Amino-acid E5 transforming protein of bovine papillomavirus requires a hydrophobic core and specific carboxyl-terminal amino acids. *Mol. Cell. Biol.* 8:4071–4078.
70. Settleman, J., A. Fazeli, …, D. DiMaio. 1989. Genetic evidence that acute morphologic transformation, induction of cellular DNA synthesis, and focus formation are mediated by a single activity of the bovine papillomavirus E5 protein. *Mol. Cell. Biol.* 9:5563–5572.
71. Kulke, R., B. H. Horwitz, …, D. DiMaio. 1992. The central hydrophobic domain of the bovine papillomavirus E5 transforming protein can be functionally replaced by many hydrophobic amino acid sequences containing a glutamine. *J. Virol.* 66:505–511.
72. Meyer, A. N., Y. F. Xu, …, D. J. Donoghue. 1994. Cellular transformation by a transmembrane peptide: structural requirements for the bovine papillomavirus E5 oncoprotein. *Proc. Natl. Acad. Sci. USA.* 91:4634–4638.
73. de Planque, M. R., J. A. Kruijtzer, …, J. A. Killian. 1999. Different membrane anchoring positions of tryptophan and lysine in synthetic transmembrane alpha-helical peptides. *J. Biol. Chem.* 274:20839–20846.
74. de Planque, M. R. R., J.-W. P. Boots, …, J. A. Killian. 2002. The effects of hydrophobic mismatch between phosphatidylcholine bilayers and transmembrane alpha-helical peptides depend on the nature of interfacially exposed aromatic and charged residues. *Biochemistry.* 41:8396–8404.
75. de Planque, M. R. R., B. B. Bonev, …, J. A. Killian. 2003. Interfacial anchor properties of tryptophan residues in transmembrane peptides can dominate over hydrophobic matching effects in peptide-lipid interactions. *Biochemistry.* 42:5341–5348.
76. Ramadurai, S., A. Holt, …, B. Poolman. 2010. Influence of hydrophobic mismatch and amino acid composition on the lateral diffusion of transmembrane peptides. *Biophys. J.* 99:1447–1454.
77. de Jesus, A. J., and T. W. Allen. 2013. The role of tryptophan side chains in membrane protein anchoring and hydrophobic mismatch. *Biochim. Biophys. Acta.* 1828:864–876.
78. Sharpe, H. J., T. J. Stevens, and S. Munro. 2010. A comprehensive comparison of transmembrane domains reveals organelle-specific properties. *Cell.* 142:158–169.
79. Chacón, K. M., L. M. Petti, …, D. DiMaio. 2014. De novo selection of oncogenes. *Proc. Natl. Acad. Sci. USA.* 111:E6–E14.
80. Duong-Ly, K. C., V. Nanda, …, K. P. Howard. 2005. The conformation of the pore region of the M2 proton channel depends on lipid bilayer environment. *Protein Sci.* 14:856–861.
81. Koehorst, R. B. M., R. B. Spruijt, …, M. A. Hemminga. 2004. Lipid bilayer topology of the transmembrane alpha-helix of M13 Major coat protein and bilayer polarity profile by site-directed fluorescence spectroscopy. *Biophys. J.* 87:1445–1455.
82. Strandberg, E., S. Esteban-Martín, …, A. S. Ulrich. 2009. Orientation and dynamics of peptides in membranes calculated from 2H-NMR data. *Biophys. J.* 96:3223–3232.
83. Strandberg, E., S. Esteban-Martín, …, J. Salgado. 2012. Hydrophobic mismatch of mobile transmembrane helices: Merging theory and experiments. *Biochim. Biophys. Acta.* 1818:1242–1249.
84. Kovacs, F. A., J. K. Denny, …, T. A. Cross. 2000. Helix tilt of the M2 transmembrane peptide from influenza A virus: an intrinsic property. *J. Mol. Biol.* 295:117–125.
85. Freeman-Cook, L. L., A. M. Dixon, …, D. DiMaio. 2004. Selection and characterization of small random transmembrane proteins that bind and activate the platelet-derived growth factor beta receptor. *J. Mol. Biol.* 338:907–920.
86. Goldstein, D. J., and R. Schlegel. 1990. The E5 oncoprotein of bovine papillomavirus binds to a 16 kd cellular protein. *EMBO J.* 9:137–145.
87. Schapiro, F., J. Sparkowski, …, S. Grinstein. 2000. Golgi alkalinization by the papillomavirus E5 oncoprotein. *J. Cell Biol.* 148:305–315.
88. Li, E., and K. Hristova. 2006. Role of receptor tyrosine kinase transmembrane domains in cell signaling and human pathologies. *Biochemistry.* 45:6241–6251.
89. Pettersen, E. F., T. D. Goddard, …, T. E. Ferrin. 2004. UCSF Chimera–a visualization system for exploratory research and analysis. *J. Comput. Chem.* 25:1605–1612.
90. Wien, F., and B. A. Wallace. 2005. Calcium fluoride micro cells for synchrotron radiation circular dichroism spectroscopy. *Appl. Spectrosc.* 59:1109–1113.
91. Wallace, B. A., and R. W. Janes. 2009. Modern techniques for circular dichroism and synchrotron radiation circular dichroism spectroscopy. *In* Advances in Biomedical Spectroscopy, *Vol. 1* B. A. Wallace, and R. W. Jones, editors. IOS Press, Amsterdam.
92. Pace, C. N., F. Vajdos, …, T. Gray. 1995. How to measure and predict the molar absorption coefficient of a protein. *Protein Sci.* 4:2411–2423.
93. Levitt, M. H., D. Suter, and R. R. Ernst. 1986. Spin dynamics and thermodynamics in solid-state NMR cross polarization. *J. Chem. Phys.* 84:4243.
94. Nevzorov, A. A., and S. J. Opella. 2007. Selective averaging for high-resolution solid-state NMR spectroscopy of aligned samples. *J. Magn. Reson.* 185:59–70.

SUPPORTING MATERIAL

Hydrophobic mismatch drives the interaction of E5 with the transmembrane segment of PDGF receptor

Dirk Windisch,[1] Colin Ziegler,[2] Stephan L. Grage,[1] Jochen Bürck,[1] Marcel Zeitler,[2] Peter L. Gor'kov,[3] and Anne S. Ulrich[1,2,*]

[1]Institute of Biological Interfaces (IBG-2) and [2]Institute of Organic Chemistry, Karlsruhe Institute of Technology, Karlsruhe, Germany; and [3]National High Magnetic Field Laboratory, Tallahassee, Florida

* correspondence to: anne.ulrich@kit.edu

SUPPORTING MATERIAL AND METHODS

SRCD and OCD sample preparation and measurements

To reconstitute the truncated ΔE5 in liposomes for circular dichroism analysis, the lyophilized protein powder and the lipids were each dissolved in dichloromethane/methanol (50/50, v/v), and insoluble parts were removed by centrifugation. An aliquot of the lipid stock solution was mixed with an aliquot of the ΔE5 stock solution to reach the desired concentrations for a nominal protein-to-lipid ratio of 1:50 (mol/mol). The solvents were evaporated by a stream of nitrogen, followed by subsequent lyophilization to remove residual organic solvent. The protein/lipid mixtures were rehydrated with water to obtain a protein concentration of ~1 mg ml, then thoroughly sonicated and additionally subjected to several freeze-thaw cycles. Afterwards, small unilamellar vesicles were formed by sonication of the lipidic suspension for 4 min in a strong ultrasonic bath. The sonication procedure was repeated 3 times. In between, the water of the ultrasonic bath was cooled down to room temperature with ice, to avoid overheating of the samples. Vesicles samples were always kept above the lipid phase transition before measurement. The synchrotron radiation circular dichroism (SRCD) spectra were collected on the UV-CD12 beamline at the ANKA storage ring (KIT, Germany). The beamline components and its experimental end-station have been previously described in detail (1,2).

For an SRCD measurement, a 3.5 µl aliquot (containing 1 mg ml^{-1} protein) of the sample was filled into a "Birkbeck-type" demountable CaF_2 cell (3). The optical path length of this cell was determined by interferometry to be 13.1 µm. Spectra between 260 and 175 nm were recorded with a scan-rate of 15 nm min^{-1} at 0.5 nm intervals, using a 1 nm spectral bandwidth, a 0.3 s lock-in time constant, a 1.5 s dwell time and three scans were averaged. For baseline correction the protein-free lipidic matrix was measured under the same conditions and subtracted from the protein spectrum to get the final spectrum. Secondary structure analysis was performed using the CONTIN algorithm provided by the DichroWeb server (4–7). Set 7 containing 48 reference spectra of proteins with well-established structures was used as a reference set for the secondary structure deconvolution (8). The quality of the fit between the experimental and back-calculated spectra corresponding to the derived secondary structure was assessed from the normalized root mean-square deviation (NRMSD), with a value of

<0.1 considered a good fit. To calculate the mean residue ellipticities that are used for secondary structure estimation, the concentration of the ΔE5 protein was determined based on the absorbance of the protein at 280 nm, using a molar extinction coefficient of 12490 L mol^{-1} cm^{-1} (9).

For oriented circular dichroism (OCD) measurements, macroscopically aligned membrane samples were prepared from the E5 vesicle suspensions above, by depositing an aliquot of the lipid suspension (containing 3 µg protein and 40-50 µg lipid) onto a quartz glass plate with a 20 mm diameter. The suspensions were allowed to dry, and samples were subsequently equilibrated under 96% relative humidity for 15 h above the lipid phase transition temperature (20°C for DOPC and DEiPC, 30°C for DErPC, 35°C for DNPC) in a sample cell for OCD measurements. This in-house built OCD cell can be integrated in a J-810 spectropolarimeter (Jasco, Groß-Umstadt, Germany) as an accessory. To reduce possible spectral artifacts caused by a variable quality of protein reconstitution, at least three independent samples were prepared, measured and finally averaged to produce the final OCD spectrum.

Solid-state NMR sample preparation and measurements:

For solid-state NMR measurements, uniformly ^{15}N-labeled ΔE5 and PDGFR-TMD were reconstituted in macroscopically aligned membranes made of DNPC, DErPC, DEiPC, DOPC, POPC and DMPC (protein-to-lipid ratio of 1:50 (mol/mol), about 2 mg of protein per sample). As for SRCD, the lyophilized protein powder and lipids were dissolved in dichloromethane/methanol (50/50, v/v) and insoluble parts were removed by centrifugation. The protein/lipid solution was spotted onto 20 glass plates (7.5 x 12 x 0.06 mm, Marienfeld GmbH & Co.KG, Germany), and the samples were allowed to dry followed by drying under vacuum overnight to remove residual organic solvent. The glass plates were then stacked and placed under a humid atmosphere (saturated K_2SO_4) for rehydration at 48°C overnight. Finally, the glass plate stacks were wrapped into parafilm and polyethylene foil to prevent drying during the NMR measurement. Solid-state ^{15}N-NMR measurements were carried out on a Bruker Avance 600 MHz spectrometer (Bruker-Biospin, Karlsruhe, Germany), using a custom-built Low-E ^{1}HX probe equipped with cross-coil resonators of rectangular cross section (KIT Karlsruhe, Germany and NHMFL Tallahassee, USA) (10). For the NMR measurements the temperature was set to 20°C for DOPC and DEiPC, 30°C for DErPC, and 35°C for DNPC samples in order to stay above the lipid phase transition temperature. The quality of the lipid alignment was checked by measuring ^{31}P-NMR spectra using a Hahn echo sequence. One-dimensional (1D) ^{15}N-NMR spectra were obtained using a CP-MOIST pulse sequence with an additional echo pulse (20 µs echo delay), with 10 ms acquisition time, 0.5 ms contact time and 50 kHz ^{1}H-decoupling (11). 25600 scans were acquired with a recycle delay time of 3 s.

For the two-dimensional separated local field measurements, the SAMMY experiment was used, with the optimized SAMPI4 pulse sequence and a 90° pulse of 4.9 µs in the SAMPI4 multi-pulse train, 10 ms acquisition time, 0.5 ms contact time, 50 kHz ^{1}H-decoupling and a recycle delay of 3 s (12,13). 1780 scans with 72 t_1 increments were acquired. A ^{1}H carrier frequency of 9 ppm and a ^{15}N carrier frequency of 180 ppm were used, which are optimal for transmembrane helices. The ^{15}N chemical shifts were referenced to an external sample of solid $(^{15}NH_4)_2SO_4$, which was set to 26.8 ppm corresponding to 0 ppm for liquid ammonia. The dipolar dimension was scaled as previously described (13).

SUPPORTING FIGURES

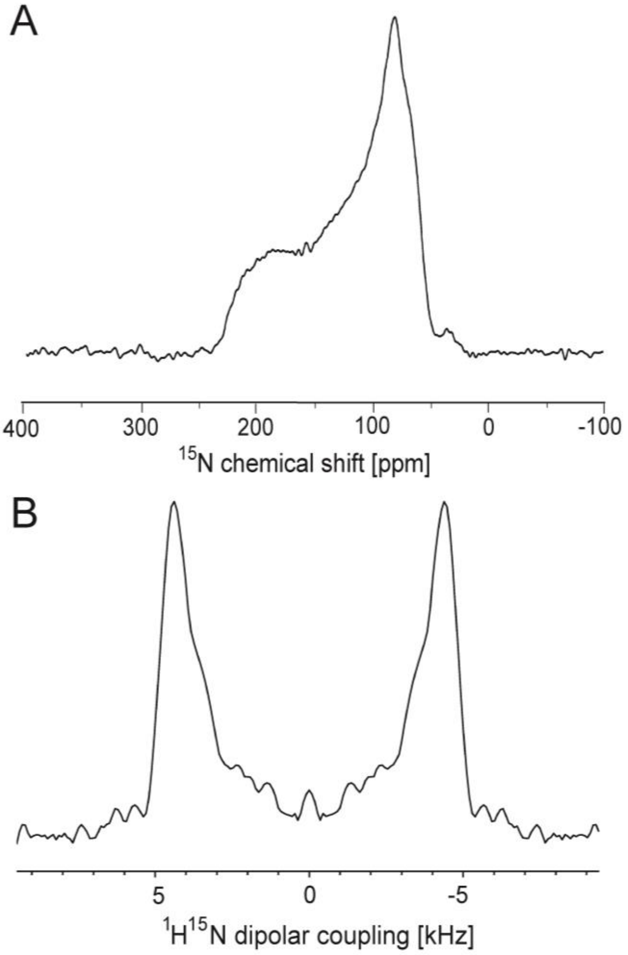

Figure S1: (*A*) A one-dimensional solid-state ^{15}N-NMR powder spectrum of ΔE5 was used to determine the principal values of the ^{15}N-CSA tensor. (*B*) A maximum ^{1}H-^{15}N dipolar coupling corresponding to a peak position (half splitting) of 8.8 kHz was determined from a SAMMY spectrum (only the projection of ^{1}H-^{15}N dipolar coupling dimension is shown) of the protein powder using the same experimental parameters as for the reconstituted protein.

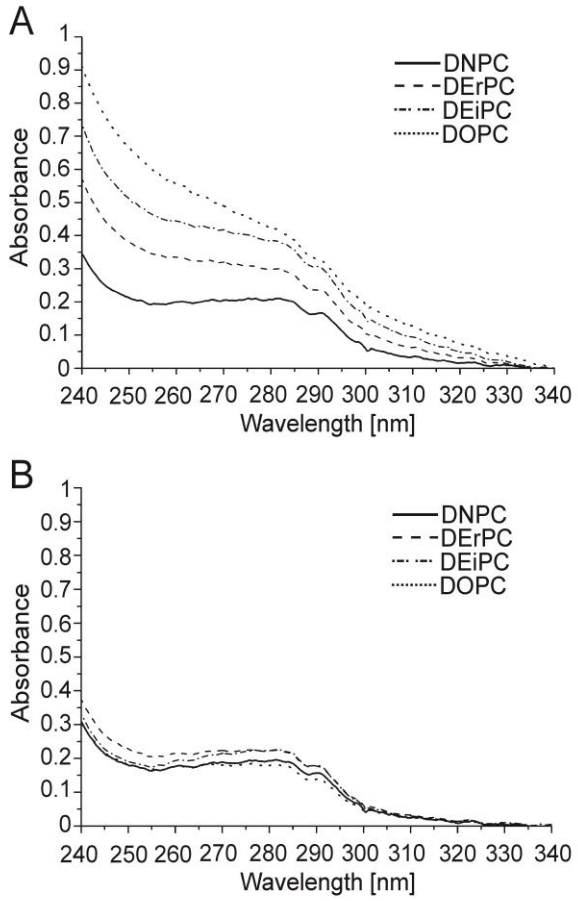

Figure S2: (*A*) UV spectra of SRCD samples of ΔE5 in different lipid vesicles showing increasing scattering contributions for decreasing bilayer thickness. (*B*) After dilution (1:20) in 10 mM SDS and ultrasound waterbath treatment (for 10 min) the aggregates are dissolved by the detergent, and now the absorption spectra match each other due to the absence of scattering contributions.

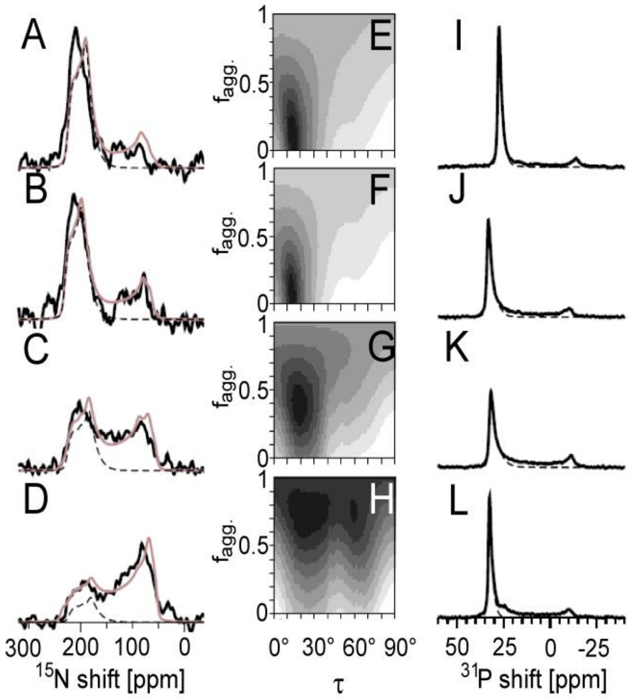

Figure S3: One-dimensional solid-state ^{15}N- and ^{31}P-NMR analysis was used to assess the membrane alignment and aggregation tendency of ΔE5 (here protein batch 2). The uniformly ^{15}N-labeled protein was reconstituted in bilayers of different thickness, namely in DNPC (*A*/*E*/*I*), DErPC (*B*/*F*/*J*), DEiPC (*C*/*G*/*K*) and DOPC (*D*/*H*/*L*). To deconvolute the experimental ^{15}N-NMR spectra (*A-D*, *solid black lines*), they were fitted with simulated lineshapes representing three fractions: well-oriented peptide, properly reconstituted peptide in mis-aligned membrane regions, and aggregated peptide. This way, the helix tilt angle of the well-oriented peptide population (*dashed lines*) could be estimated, and the sum of all three contributions is also shown (*solid grey lines*). The agreement between the calculated and experimental spectra as a function of tilt angle and aggregated fraction was judged from RMSD plots (*E-H*, black indicating the lowest RMSD value). In parallel, solid-state ^{31}P-NMR of the phospholipids (*I-L*) was used to assess the quality of alignment of the lipid matrix. For each samples the proportion of well-oriented lipid (*dashed grey line*) compared to mis-aligned membranes was obtained and used to fit the corresponding ^{15}N-spectra. In this iterative analysis, an increasing tilt angle of the E5 helix (away from the membrane normal) and an increasing amount of aggregated peptide was found with decreasing bilayer thickness upon going from DNPC to DOPC, see also Tables 2 and S1.

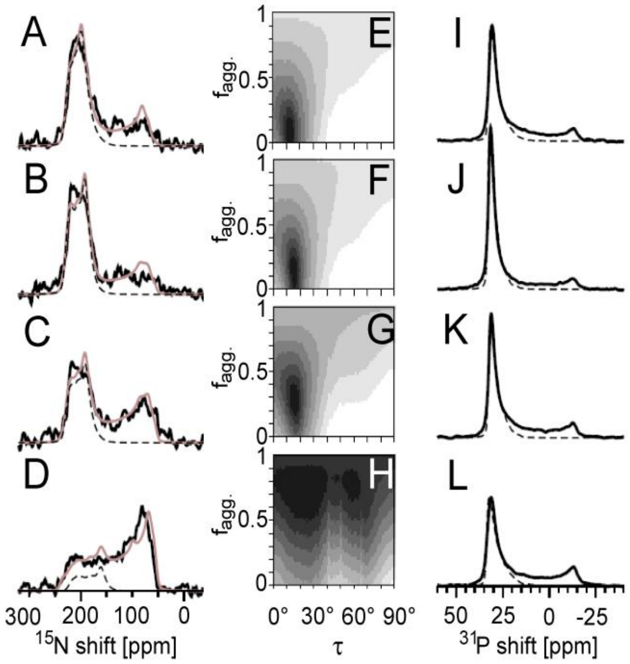

Figure S4: One-dimensional solid-state ^{15}N- and ^{31}P-NMR analysis was used to assess the membrane alignment and aggregation tendency of ΔE5 (here protein batch 3). See Figs. 2 and S3 for details.

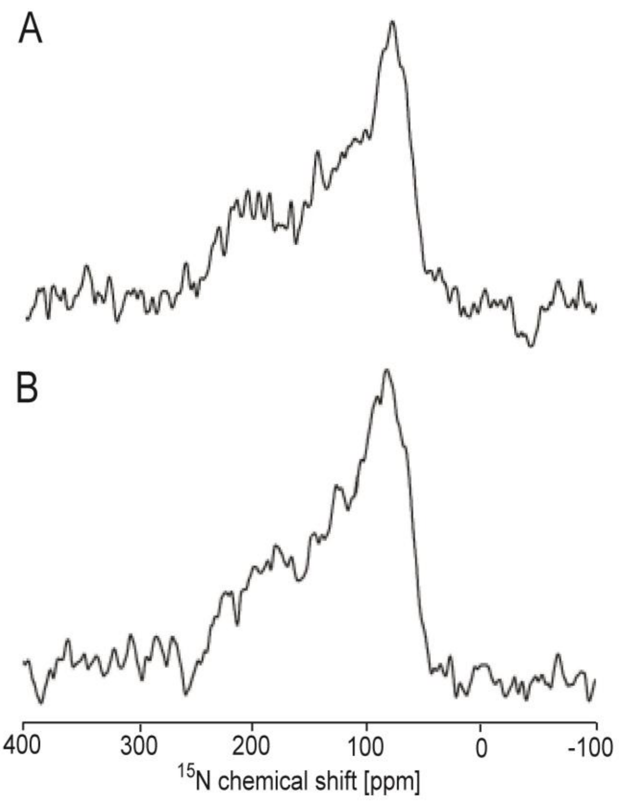

Figure S5: One-dimensional solid-state ^{15}N-NMR spectra of ΔE5 in macroscopically oriented samples of (*A*) DMPC and (*B*) POPC, acquired at 30°C and 20°C, respectively. Essentially pure powder spectra are observed due to protein aggregation in these conventional lipid bilayers.

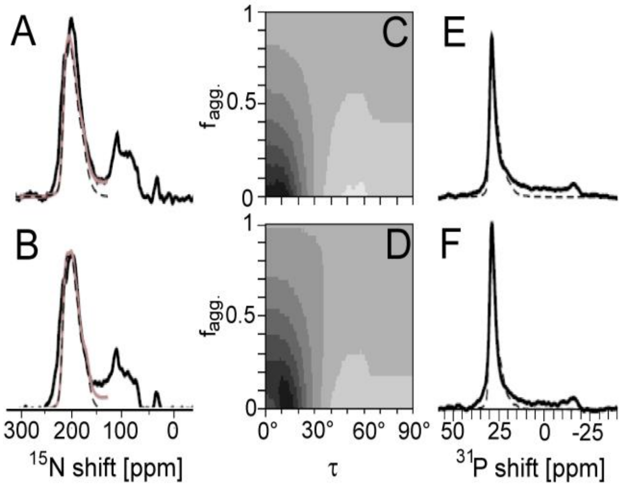

Figure S6: One-dimensional solid-state ^{15}N- and ^{31}P-NMR analysis was used to assess the membrane alignment and aggregation tendency of ^{15}N-PDGFR-TMD in presence of the ^{14}N-ΔE5 in DErPC. Two different samples were measured and analyzed. To deconvolute the experimental ^{15}N-NMR spectra (*A*/*B*), the same procedure as used for the pure PDGFR-TMD was applied. The ^{15}N-NMR spectrum shown in (*A*) corresponds to the spectrum shown in Fig. 5 *A*. The agreement between the calculated and experimental spectra as a function of tilt angle and aggregated fraction was judged from RMSD plots (*C*/*D*, black indicating the lowest RMSD value). In parallel, solid-state ^{31}P-NMR of the phospholipids (*E*/*F*) was used to assess the quality of alignment of the lipid matrix. No change of the lineshape was found compared to the spectrum of the pure protein in these bilayers (compare to Fig. 3 *B*), indicating that the tilt angle of the PDGFR-TMD helix has not changed due to the presence of the ΔE5. See Figs. 2 and S3 for details, and also Tables 3 and S2.

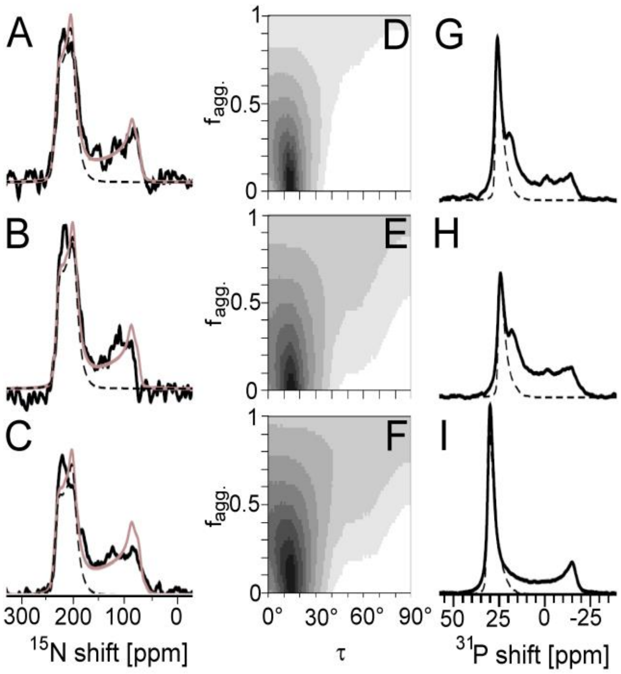

Figure S7: One-dimensional solid-state ^{15}N- and ^{31}P-NMR analysis was used to assess the membrane alignment and aggregation tendency of ^{15}N-ΔE5 in presence of the ^{14}N-PDGFR-TMD in DErPC. Three different samples were measured and analyzed. To deconvolute the experimental ^{15}N-NMR spectra (*A*-*C*), the same procedure as used for the pure ΔE5 protein was applied. The ^{15}N-NMR spectrum shown in (*A*) corresponds to the spectrum shown in Fig. 5 *B*. The agreement between the calculated and experimental spectra as a function of tilt angle and aggregated fraction was judged from RMSD plots (*D*-*F*, black indicating the lowest RMSD value). In parallel, solid-state ^{31}P-NMR of the phospholipids (*G*-*I*) was used to assess the quality of alignment of the lipid matrix. No change of the lineshape was found compared to the spectrum of the pure protein in these bilayers (compare to Fig. 2 *B*), indicating that the tilt angle of the E5 helix has not changed due to the presence of the PDGFR-TMD. See Figs. 2 and S3 for details, and also Tables 3 and S2.

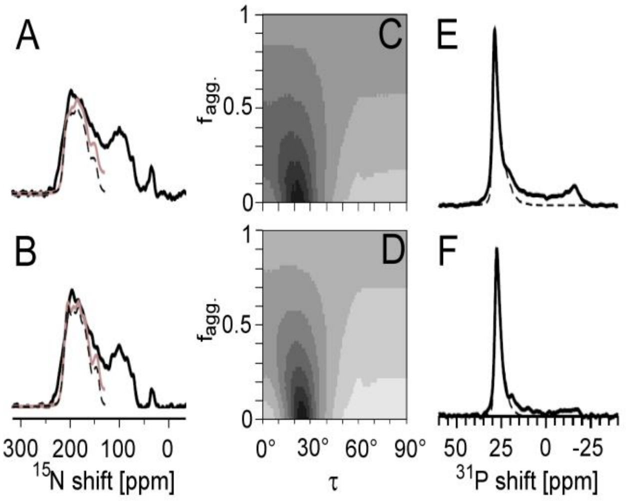

Figure S8: One-dimensional solid-state ^{15}N- and ^{31}P-NMR analysis was used to assess the membrane alignment and aggregation tendency of ^{15}N-PDGFR-TMD in presence of the ^{14}N-ΔE5 in DOPC. Two different samples were measured and analyzed. To deconvolute the experimental ^{15}N-NMR spectra (*A*/*B*), the same procedure as used for the pure PDGFR-TMD was applied. The ^{15}N-NMR spectrum shown in (*B*) corresponds to the spectrum shown in Fig. 5 *C*. The agreement between the calculated and experimental spectra as a function of tilt angle and aggregated fraction was judged from RMSD plots (*C*/*D*, black indicating the lowest RMSD value). In parallel, solid-state ^{31}P-NMR of the phospholipids (*E*/*F*) was used to assess the quality of alignment of the lipid matrix. No change of the lineshape was found compared to the spectrum of the pure protein in these bilayers (compare to Fig. 3 *D*), indicating that the tilt angle of the PDGFR-TMD helix has not changed due to the presence of the ΔE5. See Figs. 2 and S3 for details, and also Tables 3 and S2.

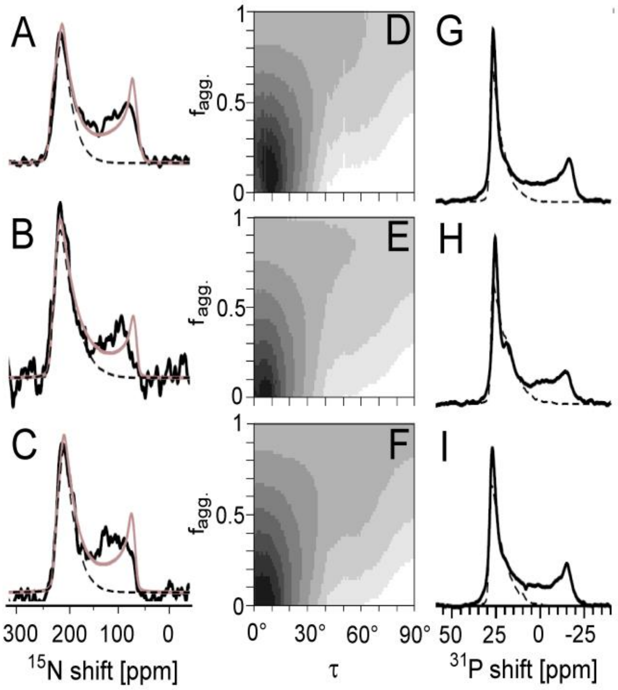

Figure S9: One-dimensional solid-state ^{15}N- and ^{31}P-NMR analysis was used to assess the membrane alignment and aggregation tendency of ^{15}N-ΔE5 in presence of the ^{14}N-PDGFR-TMD in DOPC. Three different samples were measured and analyzed. To deconvolute the experimental ^{15}N-NMR spectra (*A*-*C*), the same procedure as used for the pure ΔE5 protein was applied. The ^{15}N-NMR spectrum shown in (*A*) corresponds to the spectrum shown in Fig. 5 *D*. The agreement between the calculated and experimental spectra as a function of tilt angle and aggregated fraction was judged from RMSD plots (*D*-*F*, black indicating the lowest RMSD value). In parallel, solid-state ^{31}P-NMR of the phospholipids (*G*-*I*) was used to assess the quality of alignment of the lipid matrix. A large change of the lineshape was found compared to the spectrum of the pure protein in these bilayers (compare to Fig. 2 *D*), indicating a well-reconstituted and transmembrane aligned E5 helix caused by the presence of the PDGFR-TMD. See Figs. 2 and S3 for details, and also Tables 3 and S2.

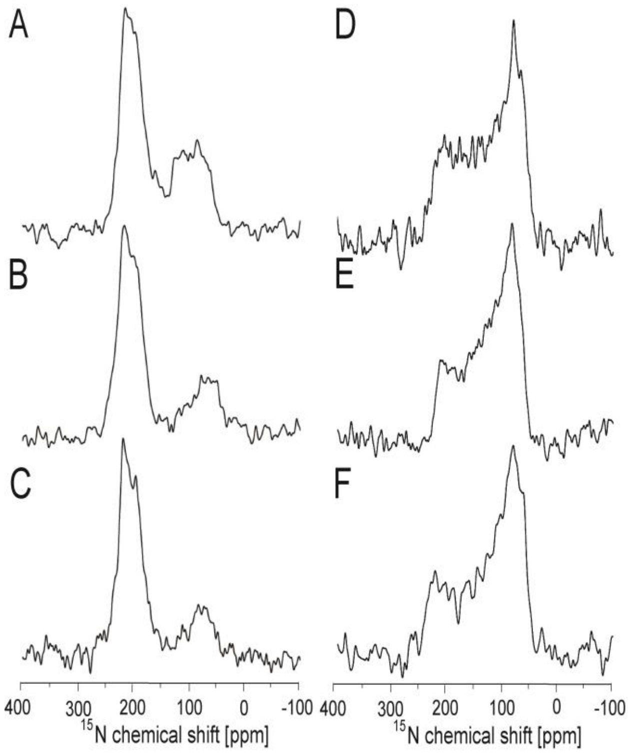

Figure S10: One-dimensional solid-state ^{15}N-NMR spectra of ΔE5 in macroscopically oriented lipid bilayers of DErPC (*A*-*C*) and DOPC (*D*-*F*) at different protein-to-lipid ratios of (*A*/*D*) 1:200, (*B*/*E*) 1:100 and (*C*/*F*) 1:50 (mol/mol). In DErPC, no changes of the transmembrane orientation were observed, indicating a protein and lipid concentration independent alignment of ΔE5. In DOPC, a powder lineshape was found at all protein-to-lipid ratios indicating that protein aggregation cannot be prevent by an increase of the lipid concentration.

SUPPORTING TABLES

Table S1: Detailed results of 1D solid-state NMR analysis of ΔE5 and PDGFR-TMD in lipid bilayers with different thickness.

ΔE5 protein batch 1 (Fig. 2)

Lipid	τ	S_{mol}	$f_{agg.}$	RMSD
**DNPC**	8°	1	22%	0.00098
**DErPC**	12°	1	10%	0.00127
**DEiPC**	16°	1	38%	0.00095
**DOPC**	16°	1	88%	0.00062

ΔE5 protein batch 2 (Fig. S3)

Lipid	τ	S_{mol}	$f_{agg.}$	RMSD
**DNPC**	15°	0.90	10%	0.00222
**DErPC**	13°	0.96	6%	0.00176
**DEiPC**	18°	0.92	38%	0.00152
**DOPC**	19°	0.90	72%	0.00158

ΔE5 protein batch 3 (Fig. S4)

Lipid	τ	S_{mol}	$f_{agg.}$	RMSD
**DNPC**	13°	0.94	6%	0.00122
**DErPC**	16°	0.94	14%	0.00132
**DEiPC**	16°	0.94	28%	0.00110
**DOPC**	28°	0.98	72%	0.00147

PDGFR-TMD (Fig. 3)

Lipid	τ	S_{mol}	$f_{agg.}$	RMSD
**DNPC**	3°	1	2%*	0.00398
**DErPC**	10°	1	0%*	0.00278
**DEiPC**	14°	1	0%*	0.00150
**DOPC**	22°	1	0%*	0.00166

1D solid-state ^{15}N-NMR analysis of the ΔE5 protein and PDGFR-TMD concerning their membrane orientation (helix tilt angle τ with respect to the bilayer normal), their molecular order parameter S_{mol} and their aggregation tendency (percentage $f_{agg.}$) in different lipid membranes. The uniformly ^{15}N-labeled ΔE5 and PDGFR-TMD were reconstituted in lipid bilayers of DNPC, DErPC, DEiPC and DOPC. For ΔE5 three individual samples (derived from three different protein batches) per lipid were analysed as described in the text. *For PDGFR-TMD the fraction of aggregated protein is less reliable, as the up-field part of the 1D-NMR spectrum (<130 ppm) was not included in the simulations (see text and Fig. 3). The agreement of the calculated and experimental spectra as a function of tilt angle and aggregated fraction was judged by the root of mean-square deviation (RMSD).

Table S2: Detailed results of 1D solid-state NMR analysis of the ΔE5/PDGFR-TMD mixtures in lipid bilayers with different thickness.

Mixture	Lipid	τ	S_{mol}	$f_{agg.}$	RMSD
**^{15}N-PDGFR-TMD/^{14}N-ΔE5 (1:1)**	**DErPC**	13°	1	0%*	0.00051
		10°	1	0%*	0.00258
	**DOPC**	24°	1	0%*	0.00037
		22°	1	0%*	0.00221
**^{15}N-ΔE5/^{14}N-PDGFR-TMD (1:1)**	**DErPC**	14°	0.9	0%	0.00030
		13°	0.92	2%	0.00108
		14°	0.92	8%	0.00025
	**DOPC**	9°	1	10%	0.00115
		6°	0.98	0	0.00031
		7°	0.92	0	0.00030

1D solid-state ^{15}N-NMR analysis of the ΔE5 protein and PDGFR-TMD concerning their membrane orientation (helix tilt angle τ with respect to the bilayer normal), their molecular order parameter S_{mol} and their aggregation tendency ($f_{agg.}$) in different lipid membranes, each protein in presence of the respective other one. For ΔE5 three individual samples per lipid and for the PDGFR-TMD two samples were analysed. *For PDGFR-TMD the fraction of aggregated protein is less reliable, as the up-field part of the 1D-NMR spectrum (<130 ppm) was not included in the simulations (see text and Fig. 3). The agreement of the calculated and experimental spectra as a function of tilt angle and aggregated fraction was judged by the root of mean-square deviation (RMSD).

SUPPORTING REFERENCES

1. Clarke, D. T., and G. Jones. 2004. CD12: a new high-flux beamline for ultraviolet and vacuum-ultraviolet circular dichroism on the SRS, Daresbury. *J Synchrotron Radiat,* 11:142–149.
2. Bürck, J., S. Roth, D. Windisch, P. Wadhwani, D. Moss, and A. S. Ulrich. 2015. UV-CD12: synchrotron radiation circular dichroism beamline at ANKA. *J Synchrotron Radiat,* 22:844–852.
3. Wien, F., and B. A. Wallace. 2005. Calcium fluoride micro cells for synchrotron radiation circular dichroism spectroscopy. *Appl Spectrosc,* 59:1109–1113.
4. Sreerama, N., and R. W. Woody. 2000. Estimation of protein secondary structure from circular dichroism spectra: comparison of CONTIN, SELCON, and CDSSTR methods with an expanded reference set. *Anal. Biochem.,* 287:252–260.
5. van Stokkum, I. H., H. J. Spoelder, M. Bloemendal, R. van Grondelle, and F. C. Groen. 1990. Estimation of protein secondary structure and error analysis from circular dichroism spectra. *Anal. Biochem.,* 191:110–118.
6. Whitmore, L., and B. A. Wallace. 2004. DICHROWEB, an online server for protein secondary structure analyses from circular dichroism spectroscopic data. *Nucleic Acids Res.,* 32:W668-73.
7. Lobley, A., L. Whitmore, and B. A. Wallace. 2002. DICHROWEB: an interactive website for the analysis of protein secondary structure from circular dichroism spectra. *Bioinformatics,* 18:211–212.
8. Wallace, B. A., and R. W. Janes. 2009. Modern techniques for circular dichroism and synchrotron radiation circular dichroism spectroscopy. *In* Volume 1 Advances in Biomedical Spectroscopy, B. A. Wallace and R. W. Jones, editors, IOS Press, Amsterdam.
9. Pace, C. N., F. Vajdos, L. Fee, G. Grimsley, and T. Gray. 1995. How to measure and predict the molar absorption coefficient of a protein. *Protein Sci.,* 4:2411–2423.
10. Gor'kov, P. L., E. Y. Chekmenev, C. Li, M. Cotten, J. J. Buffy, N. J. Traaseth, G. Veglia, and W. W. Brey. 2007. Using low-E resonators to reduce RF heating in biological samples for static solid-state NMR up to 900 MHz. *J. Magn. Reson.,* 185:77–93.
11. M.H. Levitt, D. S. a. R. E. 1986. Spin dynamics and thermodynamics in solid-state NMR cross polarization. *J. Chem. Phys.,* 1986:4243–4255.
12. Nevzorov, A. A., and S. J. Opella. 2003. A "magic sandwich" pulse sequence with reduced offset dependence for high-resolution separated local field spectroscopy. *J. Magn. Reson.,* 164:182–186.
13. Nevzorov, A. A., and S. J. Opella. 2007. Selective averaging for high-resolution solid-state NMR spectroscopy of aligned samples. *J. Magn. Reson.,* 185:59–70.
